# Interventions for reducing blood pressure in prehypertension: A meta-analysis

**DOI:** 10.3389/fpubh.2023.1139617

**Published:** 2023-03-23

**Authors:** Wenjing Li, Hao Liu, Xinai Wang, Jingying Liu, Hongling Xiao, Chenqi Wang, Yaxuan Wu

**Affiliations:** ^1^The School of Graduate, Tianjin University of Traditional Chinese Medicine, Tianjin, China; ^2^The School of Nursing, Tianjin University of Traditional Chinese Medicine, Tianjin, China

**Keywords:** prehypertension, hypertension, meta-analysis, aerobic exercise, dietary approaches to stop hypertension (DASH)

## Abstract

**Background:**

We aimed to address which interventions best control blood pressure (BP) and delay disease progression in prehypertension and to give recommendations for the best option following a quality rating.

**Methods:**

A Bayesian network meta-analysis was used to assess the effect of the intervention on BP reduction, delaying hypertension progression and final outcome, with subgroup analyses for time and ethnicity. Recommendations for interventions were finally based on cumulative ranking probabilities and CINeMA.

**Results:**

From 22,559 relevant articles, 101 eligible randomized controlled trial articles (20,176 prehypertensive subjects) were included and 30 pharmacological and non-pharmacological interventions were evaluated. Moderate-quality evidence demonstrated that angiotensin II receptor blockers, aerobic exercise (AE), and dietary approaches to stop hypertension (DASH) lowered systolic blood pressure (SBP). For lowering diastolic blood pressure (DBP), AE combined with resistance exercise (RE) or AE alone provided high quality evidence, with calcium channel blockers, lifestyle modification (LSM) combined with drug providing moderate quality evidence. LSM produced the best BP lowering effect at 12 months and beyond of intervention. In Asians, TCD bubble was moderate quality evidence for lowering SBP and RE may have had a BP lowering effect in Caucasians. No recommendation can be given for delaying the progression of hypertension and reducing mortality outcomes because of low to very low quality of evidence.

**Conclusion:**

AE combined RE are preferentially recommended for BP control in prehypertension, followed by DASH. Long-term BP control is preferred to LSM. Asians and Caucasians add TCD bubble and RE to this list as potentially effective interventions.

**Systematic review registration:**

https://www.crd.york.ac.uk/prospero/display_record.php?ID=CRD42022356302, identifier: CRD42022356302.

## 1. Introduction

Hypertension is one of the strongest risk factors for cardiovascular disease (CVD) and stroke affecting the global population (1). Prehypertension (PHT), as a transitional stage from ideal blood pressure (BP) to hypertension, with typical threshold values of 120–139 mmHg systolic or 80–89 mmHg diastolic (2, 3), provides clinicians with criteria for the need for BP management and treatment to control its progression to hypertension (HT) and to prevent subclinical damage to cardiovascular target organs (4).

Non-pharmacological treatment is mostly recommended for PHT compared to the first-line pharmacological management recommended for HT. Secondary prevention with a single BP-lowering medication is only recommended when the patient has diabetes, is at an increased risk of CVD (5) or is approaching a threshold (140/90 mmHg) (3). Recent trials (6), meta-analyses (7), and additions to the guidelines (8) have added important information for these questions on the intervention thresholds and protocols for PHT, with people across different levels of BP being able to take medication for BP-lowering management. The risk of major adverse cardiovascular events is reduced by ~10% when SBP is reduced by 5 mmHg, benefitting people with PHT with or without CVD risk.

Overall, the results of the 2015 SPRINT study (9) have led to the development of a more enhanced concept of BP. With this in mind, BP control needs to be tailored to the characteristics of the PHT population. A large number of studies have provided ample evidence to support the effectiveness of lifestyle modification (LSM) (10, 11) such as increasing physical activity and modifying dietary habits, in reducing BP in people with PHT. However, it is the high calorie food consumption and sedentary lifestyle habits that conflict with LSM and affect adherence to implementation in people with PHT. A number of studies are now emerging that provide evidence for pharmacological treatment, but long-term drug use places a financial burden on the healthcare system and families (12). Previous studies have included BP across both PHT and HT populations, and fewer studies have discussed pharmacological and non-pharmacological interventions together. Therefore, a reasonable measure of the effectiveness of different interventions in lowering BP in the PHT population warrants detailed consideration to arrive at the most reasonable BP-lowering regimen.

This study is the first network meta-analysis (NMA) to include PHT as a study population and this paper will systematically review all randomized controlled trials (RCTs) of pharmacological and non-pharmacological interventions in the PHT population. In addition to examining the effect of BP lowering, this article specifically examines HT progression rates and cardiac, cerebral, renal and mortality outcomes. Exploring the optimal intervention options as an important part of evidence synthesis and decision making in healthcare provides clinicians with recommendations for the best interventions.

## 2. Methods

This article is registered on the Prospero website (CRD42022356302, https://www.crd.york.ac.uk/PROSPERO/display_record.php?RecordID=356302) and follows the PRISMA checklist (Supplementary Table 1).

### 2.1. Search strategy

A search formula was developed based on the research strategy (Supplementary Table 2). Seven electronic databases—PubMed, The Cochrane Library, SCOPUS, Web of Science, CNKI, Wanfang Data, VIP were searched from the inception of the databases to 5 October 2022, a process that did not restrict the language of the original articles. In addition, references to the included literature are reviewed to avoid article omissions.

The screening process was carried out independently by two researchers (Wj L, Xa W) and a third independent reviewer (Hao L) was consulted in case of disagreement.

### 2.2. Inclusion and exclusion criteria

Inclusion criteria: (1) The article is a randomized controlled trial; (2) participants with prehypertension between 120 and 139 mmHg systolic and/or 80–89 mmHg diastolic were included; (3) the intervention group was given at least 4 week and more of intervention; (4) outcomes included BP values before and after the intervention.

Exclusion criteria: (1) Participants with co-morbid diabetes or cardiovascular disease; (2) participants were children/adolescents or pregnant women; (3) incomplete data from the trial were not available to extract the required data; (4) articles in which participants were taking anti-hypertensive medication other than the RCT in which the pharmacological intervention was performed; (5) duplicate articles, systematic reviews, conference papers, and animal studies.

### 2.3. Data extraction

A standardized extraction form was used to extract article information, study population information, intervention protocol, and outcomes (increased clinical incidence of hypertension progression and adverse outcomes compared to the original protocol). Data were extracted and cross-checked against the records to verify the consistency of the data.

When the results of a study were unclear or incomplete, we contacted the author by email to obtain relevant information. If data were still not available, the study was excluded.

### 2.4. Quality assessment

Two reviewers (Wj L and Cq W) independently identified the risk of bias using the Cochrane Risk of Bias tool. Articles were considered low risk of bias when the number assessed as low except for the “Blinding of participants and personnel” section (13) was ≤ 3, high when there was a high bias rating, and uncertain risk of bias in the remaining cases. In case of disagreement, the decision was made after discussion with a third investigator (Yx W).

Funnel plot analysis was used when the outcome indicator contained 10 or more trials and quantitative estimates were made using egger tests to determine whether there was potential publication bias and small sample effects in the articles.

### 2.5. Data analysis

The results were analyzed using Stata 17.0 software and R 4.2.2 software. The code used is publicly available. For continuous variables outcome indicators, the mean deviation (MD) and standard deviation (SD) before and after the intervention were used as effect sizes, and when mean and interquartile data were available, they were converted in accordance with guidelines (14). For the count data the odds ratio (OR) and its 95% confidence interval (CI) were used as the effect analysis statistic.

Heterogeneity was assessed using the Cochran Q test and the *I*^2^ heterogeneity test, and data with *I*^2^ > 50% heterogeneity were subjected to subgroup or sensitivity analysis. A random effects model and a fixed effects model were fitted separately for statistical analysis, and the degree of fit of the models was judged according to the deviance information criterion (DIC) values to select an appropriate model.

Forest plots of outcome indicators were drawn and two-by-two comparisons of the efficacy of each intervention were made. To ensure consistency of evidence for direct and indirect comparisons, inconsistency tests were performed using nodal splitting. The area under the cumulative probability ranking curve under the Bayesian model was calculated in R language to visually estimate the treatment rank of each intervention (15), with SUCRA expressed as a percentage between 0 (when the treatment was determined to be the worst) and 100% (16) (when the treatment was determined to be the best), presenting the likelihood of each intervention being the best.

Subgroup analyses were also conducted to differentiate between interventions, for interventions longer than 12 months, and for populations from different ethnic and cultural backgrounds, to address the heterogeneity of the study and to make targeted recommendations for the population.

### 2.6. Certainty of evidence

The quality of evidence for the NMA analysis was graded using the CINeMA program, an online mesh Meta-analysis based on the GRADE method developed by Salanti et al. (17). The quality rating was assessed by the “netmeta” package of the R software and the calculation of the contribution matrix of the NMA (18). The results of the NMA were assessed overall and the quality of evidence was rated as high, moderate, low, and very low.

## 3. Data synthesis and analysis

**Figure 1:**
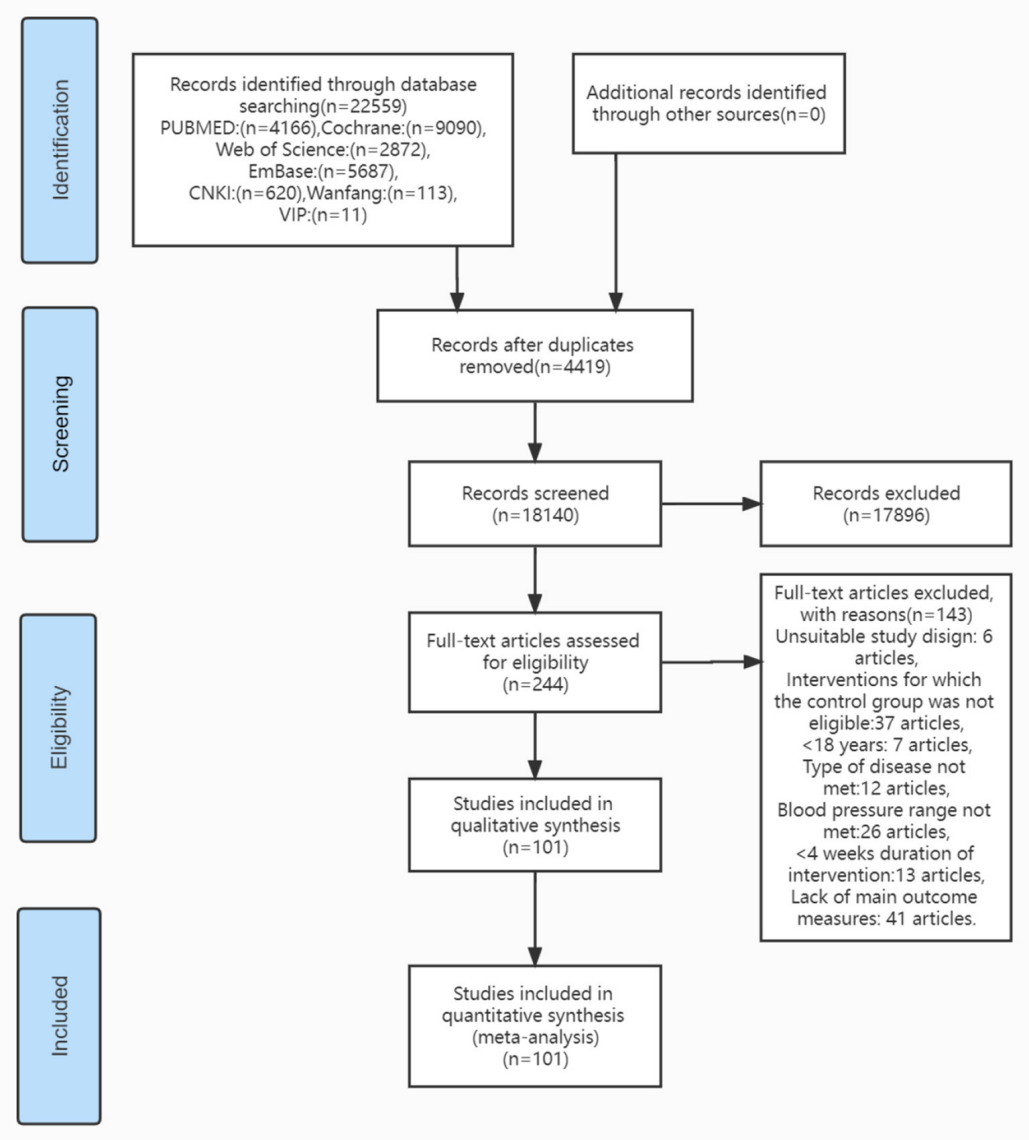
Database search and selection process.

A total of 22,559 articles were generated by searching the database, 4,419 duplicate studies were first removed, 17,896 studies that were not relevant to the article were excluded after reading the title and abstract, and 101 studies were included for meta-analysis after assessing full-text article eligibility (Figure 1), reporting on SBP (99 articles), DBP (97 articles), progression of hypertension (22 articles), and cardiovascular outcomes (five articles). This included 49 English articles, 51 Chinese articles, and one Spanish article.

### 3.1. Characteristics of the included literature

The data from the included studies, summarized in this paper (Supplementary Table 3), show the main characteristics relevant to the purpose of this review. The 101 articles included 108 studies from Asia (*n* = 75), North America (*n* = 15), Europe (*n* = 6), South America (*n* = 3), and Oceania (*n* = 2). A total of 20,176 participants were included whose mean age was 47.21 years, of which 54.70% were male. The majority of RCTs used the JNC7 definition of prehypertension (*n* = 83), including SBP of 120–139 mmHg and/or DBP of 80–89 mmHg. Of the included RCTs, all were two-arm studies, except for 17 three-arm trials, 2 four-arm trials, and 1 five-arm trial. The median duration of intervention was 12 weeks (range 4 weeks to 6 years) and 32 studies were ≥12 months in length.

### 3.2. Model examination

Under the fixed effects model, DIC = 430.6918, *I*^2^ = 0% for SBP, and DIC = 420.5391, *I*^2^ = 0.3% for DBP. Under the random effects model, DIC for SBP = 2,980.4307, DIC for DBP = 1,795.0285. The fixed effects model with smaller DIC values and better fit was used as the model for data analysis (Supplementary Table 4).

### 3.3. Risk of bias

The risk of bias was assessed using the Cochrane assessment tool (Supplementary Table 5) and 81 articles were classified as “low bias,” 4 as “moderate bias,” and 16 as “high bias.” The risk of bias was mainly due to the lack of random sequence generation during randomization (*n* = 51) and the inability to achieve complete double-blindness (*n* = 74) due to the majority of LSM in this paper, and the inevitable degree of dislocation and progression of patients from prehypertension to hypertension (*n* = 26) as the duration of interventions was extended in the included studies.

Studies containing both BP and hypertension progression outcome indicators were above 10 and publication bias was assessed using funnel plots. The results showed more symmetry (Supplementary Figure 1) and further quantitative analysis using the Egger test showed better results without significant publication bias (Supplementary Table 6).

### 3.4. Results

**Figure 2:**
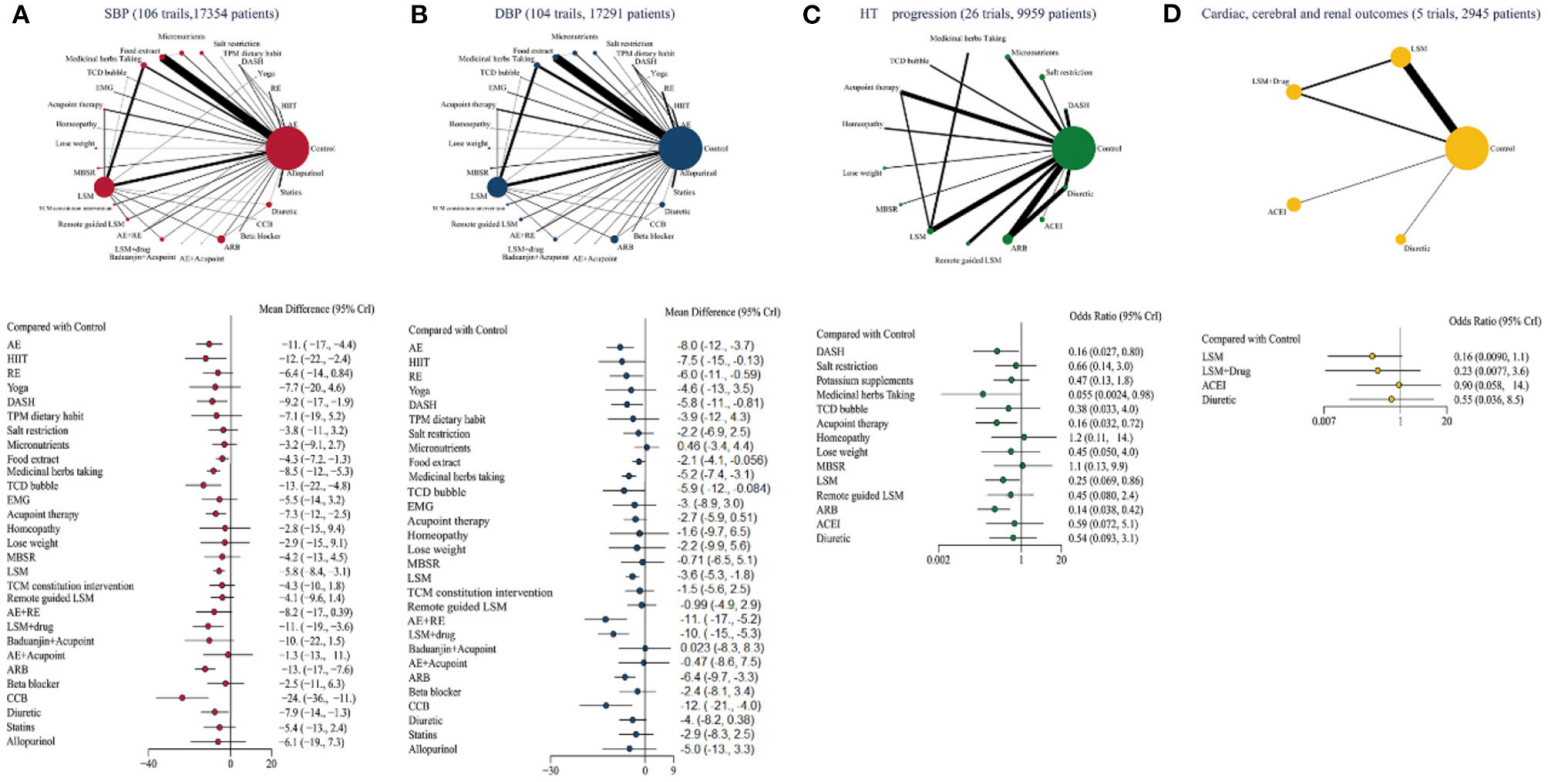
Network geometry and forest plot in PHT with four outcomes. **(A)** SBP (106 trails, 17,354 patients), **(B)** DBP (104 trails, 17,291 patients), **(C)** HT progression (26 trails, 9,959 patients), **(D)** Cardiac, cerebral, renal, and mortality outcomes (five trails, 2,945 patients). The difference among each comparison is visualized with forest plot, and the effect size is labeled on the right-hand side. AE, aerobic exercise; HIIT, high-intensity interval training; RE, resistance exercise; DASH, dietary approaches to stop hypertension; TPM, Traditional Persian Medicine; TCD, traditional Chinese drug; EMG, electromyographic; MBSR, mindfulness-based stress reduction; LSM, lifestyle modification; TCM, Traditional Chinese Medicine; ARB, angiotensin II receptor blockers; ACE, angiotensin-converting enzyme inhibitors; CCB, calcium channel blockers.

There were 106, 104, 26, and five trials respectively that provided available studies that included SBP, DBP, hypertension progression and cardiac, cerebral, renal, and mortality outcomes (Figure 2), forming a triangular closed loop indicating both direct, and indirect evidence in the comparison of efficacy. Figure 2, Supplementary Figure 2, and Supplementary Tables 7–13 show the antihypertensive efficacy, priority and quality of evidence for each intervention, respectively.

Twelve measures with moderate to very low quality evidence were likely to reduce SBP compared with controls (Table 1). In direct vs. indirect comparisons, the best efficacy was obtained with calcium channel blockers (CCB), followed by the angiotensin II receptor antagonist (ARB; WMD −12.5, 95% CrI −17.59, −7.54; moderate quality). TCD bubble, high-intensity intermittent exercise (HIIT), lifestyle modification (LSM) combined with medication, aerobic exercise (AE; WMD −10.65, 95% CrI −17.12, −4.32; moderate quality) also have some hypotensive effect.

**Table 1:** Summary of findings on the efficacy of various interventions to reduce SBP.

**Intervention**	**Direct comparisons/ participant**	**Relative effect (95% CI)**	**Certainty of evidence (CINEMA)**	**SUCRA**
CCB	1 RCTs; 68 participants	**−23.67 (−36.34**, **−11)**	⊕⊕ OO	97.76%
ARB	6 RCTs; 3,044 participants	**−12.5 (−17.59**, **−7.54)**	⊕⊕⊕ O	82.88%
TCD bubble	2 RCTs; 220 participants	**−13.44 (−22**, **−4.64)**	⊕⊕ OO	82.37%
HIIT	2 RCTs; 44 participants	**−12.46 (−22.42**, **−2.39)**	⊕⊕ OO	77.58%
LSM + drug	2 RCTs; 924 participants	**−11.07 (−18.5**, **−3.49)**	⊕⊕ OO	74.03%
AE	2 RCTs; 138 participants	**−10.65 (−17.12**, **−4.32)**	⊕⊕⊕ O	73.01%
Baduanjin + Acupoint	1 RCTs; 118 participants	−10.48 (−22.33, 1.6)	⊕ OOO	67.10%
DASH	2 RCTs; 332 participants	**−9.21 (−16.47**, **−1.67)**	⊕⊕⊕ O	64.40%
Medicinal herbs taking	17 RCTs; 1,890 participants	**−8.51 (−11.71**, **−5.33)**	⊕⊕ OO	63.17%
AE + RE	2 RCTs; 70 participants	−8.23 (−16.72, 0.38)	⊕⊕⊕ O	58.25%
Diuretic	1 RCTs; 730 participants	**−7.92 (−14.44**, **−1.24)**	⊕⊕ OO	57.27%
Yoga	1 RCTs; 100 participants	−7.73 (−20.29, 4.44)	⊕ OOO	54.43%
Acupoint therapy	7 RCTs; 513 participants	**−7.3 (−12.1**, **−2.57)**	⊕ OOO	54.16%
TPM dietary habit	1 RCTs; 84 participants	−7.13(−19.32, 5.19)	⊕ OOO	51.52%
RE	3 RCTs; 70 participants	−6.54 (−13.87, 0.71)	⊕ OOO	48.80%
Allopurinol	1 RCTs; 72 participants	−5.95 (−19.53, 7.46)	⊕ OOO	45.96%
EMG	2 RCTs; 71 participants	−5.6 (−14.46, 3.32)	⊕ OOO	43.29%
LSM	9 RCTs; 2,856 participants	**−5.77 (−8.44**, **−3.12)**	⊕ OOO	43.18%
Statins	3 RCTs; 203 participants	−5.32 (−13.06, 2.41)	⊕ OOO	41.44%
MBSR	2 RCTs; 592 participants	−4.23 (−12.9, 4.44)	⊕ OOO	35.54%
TCM constitution intervention	4 RCTs; 617 participants	−4.27 (−10.44, 1.8)	⊕ OOO	34.39%
Remote guided LSM	5 RCTs; 1,035 participants	−4.1 (−9.67, 1.35)	⊕ OOO	33.11%
Food extract	19 RCTs; 1,561 participants	**−4.27 (−7.24**, **−1.31)**	⊕⊕⊕ O	32.79%
Salt restriction	3 RCTs; 1,177 participants	−3.86 (−10.95, 3.15)	⊕ OOO	32.63%
Lose weight	1 RCTs; 564 participants	−2.82 (−14.79, 9.11)	⊕ OOO	31.11%
Homeopathy	1 RCTs; 84 participants	−2.72 (−14.95, 9.27)	⊕ OOO	30.77%
Micronutrients	3 RCTs; 734 participants	−3.27 (−8.99, 2.61)	⊕⊕ OO	28.08%
Beta blocker	2 RCTs; 115 participants	−2.56 (−11.32, 6.31)	⊕ OOO	26.76%
AE + Acupoint	1 RCTs; 100 participants	−1.42 (−13.45, 10.76)	⊕ OOO	24.93%
Control				9.31%

CI, confidence interval; RR, risk ratio; SUCRA, surface under the cumulative ranking; AE, aerobic exercise; HIIT, high-intensity interval training; RE, resistance exercise; DASH, dietary approaches to stop hypertension; TPM, Traditional Persian Medicine; TCD, traditional Chinese drug; EMG, electromyographic; MBSR, mindfulness-based stress reduction; LSM, lifestyle modification; TCM, Traditional Chinese Medicine; ARB, angiotensin II receptor blockers; ACE, angiotensin-converting enzyme inhibitors; CCB, calcium channel blockers.

⊕ ⊕ ⊕ O, moderate quality.

⊕ ⊕ OO, low quality.

⊕ OOO, very low quality.

Interventions with high quality evidence for DBP reduction emerged in the comparison (Table 2), such as AE combined with resistance exercise (RE; WMD −11.02, 95% CrI −16.73, −5.21; high quality) and AE (WMD −8.03, 95% CrI −12.25, −3.69; high quality) achieving good BP reduction with credible results. The remaining interventions with moderate to low quality evidence were CCB (WMD −12.44, 95% CrI −20.81, −4.04; moderate quality), LSM+drug (WMD −10.08, 95% CrI −14.93, −5.28; moderate quality), HIIT (WMD −7.46, 95% CrI −14.89, −0.13; moderate quality), ARB, RE, DASH, TCD bubble, and Food extract.

**Table 2:** Summary of findings on the efficacy of various interventions to reduce DBP.

**Intervention**	**Direct comparisons/ participant**	**Relative effect (95% CI)**	**Certainty of evidence (CINEMA)**	**SUCRA**
CCB	1 RCTs; 68 participants	**−12.44 (−20.81**, **−4.04)**	⊕⊕⊕ O	92.78%
AE + RE	2 RCTs; 70 participants	**−11.02 (−16.73**, **−5.21)**	⊕⊕⊕⊕	91.89%
LSM + drug	2 RCTs; 924 participants	**−10.08 (−14.93**, **−5.28)**	⊕⊕⊕ O	89.70%
AE	2 RCTs; 138 participants	**−8.03 (−12.25**, **−3.69)**	⊕⊕⊕⊕	82.95%
HIIT	2 RCTs; 44 participants	**−7.46 (−14.89**, **−0.13)**	⊕⊕⊕ O	74.14%
ARB	6 RCTs; 3,044 participants	**−6.42 (−9.69**, **−3.26)**	⊕⊕ OO	72.83%
RE	3 RCTs; 70 participants	**−6 (−11.24**, **−0.59)**	⊕⊕⊕ O	67.47%
DASH	2 RCTs; 332 participants	**−5.81 (−10.8**, **−0.81)**	⊕⊕⊕ O	66.09%
TCD bubble	2 RCTs; 220 participants	**−5.87 (−11.7**, **−0.08)**	⊕⊕⊕ O	66.01%
Medicinal herbs taking	17 RCTs; 1,890 participants	**−5.24 (−7.42**, **−3.09)**	⊕⊕ OO	64.51%
Allopurinol	1 RCTs; 72 participants	−5.03 (−13.31, 3.32)	⊕ OOO	57.75%
Yoga	1 RCTs; 100 participants	−4.6 (−12.63, 3.52)	⊕⊕ OO	54.91%
Diuretic	1 RCTs; 730 participants	−3.95 (−8.24, 0.38)	⊕⊕ OO	51.59%
TPM dietary habit	1 RCTs; 84 participants	−3.9 (−12.01, 4.29)	⊕ OOO	49.91%
LSM	9 RCTs; 2,856 participants	−3.56 (−5.33, −1.79)	⊕ OOO	48.42%
EMG	2 RCTs; 71 participants	−2.98 (−8.93, 3)	⊕ OOO	43.29%
Statins	3 RCTs; 203 participants	−2.85 (−8.27, 2.53)	⊕⊕ OO	42.25%
Acupoint therapy	7 RCTs; 513 participants	−2.69 (−5.86, 0.51)	⊕ OOO	40.09%
Beta blocker	2 RCTs; 115 participants	−2.42 (−8.09, 3.44)	⊕ OOO	38.80%
Lose weight	1 RCTs; 564 participants	−2.2 (−9.9, 5.64)	⊕⊕ OO	38.15%
Salt restriction	3 RCTs; 1,177 participants	−2.18 (−6.91, 2.46)	⊕⊕ OO	36.34%
Food extract	18 RCTs; 1,521 participants	**−2.06 (−4.08**, **−0.06)**	⊕⊕⊕ O	34.16%
Homeopathy	1 RCTs; 84 participants	−1.58 (−9.68, 6.49)	⊕⊕ OO	34.11%
TCM constitution intervention	4 RCTs; 617 participants	−1.54 (−5.56, 2.46)	⊕ OOO	30.40%
AE + Acupoint	1 RCTs; 100 participants	−0.47 (−8.58, 7.51)	⊕⊕ OO	26.97%
Remote guided LSM	4 RCTs; 1,012 participants	−0.99 (−4.92, 2.91)	⊕⊕ OO	25.77%
MBSR	2 RCTs; 592 participants	−0.71 (−6.49, 5.07)	⊕⊕ OO	25.74%
Baduanjin + Acupoint	1 RCTs; 118 participants	0.02 (−8.28, 8.35)	⊕ OOO	24.22%
Micronutrients	3 RCTs; 734 participants	0.46 (−3.41, 4.37)	⊕⊕ OO	15.21%
Control				15.18%

CI, confidence interval; RR, risk ratio; SUCRA, surface under the cumulative ranking; AE, aerobic exercise; HIIT, high-intensity interval training; RE, resistance exercise; DASH, dietary approaches to stop hypertension; TPM, Traditional Persian Medicine; TCD, traditional Chinese drug; EMG, electromyographic; MBSR, mindfulness-based stress reduction; LSM, lifestyle modification; TCM, Traditional Chinese Medicine; ARB, angiotensin II receptor blockers; ACE, angiotensin-converting enzyme inhibitors; CCB, calcium channel blockers.

⊕ ⊕ ⊕ ⊕, high quality.

⊕ ⊕ ⊕ O, moderate quality.

⊕ ⊕ OO, low quality.

⊕ OOO, very low quality.

Analysis of the 27 trials that included HT progression (Supplementary Table 14) yielded ARB (OR 0.13, 95% CrI 0.03, 0.45; low quality), Acupoint therapy (OR 0.15, 95% CrI 0.03, 0.78; low quality), LSM (OR 0.25, 95% CrI 0.06, 0.94; low quality), and DASH (OR 0.5, 95% CrI 0.04, 5.83; low quality) were effective interventions but were not recommended due to the low quality of evidence.

Only five studies included data on cardiorenal and mortality outcomes (Supplementary Table 15) and statistical analysis failed to produce meaningful data and no recommendation was made in this area.

### 3.5. Sensitivity analysis and subgroup analysis

The large variety of studies included in this paper produced a more pronounced heterogeneity. The heterogeneity of EMG, AE combined with RE decreased when subgroup analyses were performed according to interventions (Supplementary Table 16); excluding studies of low to moderate quality on this basis did not change the heterogeneity significantly and the results were more stable.

A subgroup analysis was performed on 32 studies over 12 months (Table 3). Of a total of 15 interventions, only LSM achieved reductions in SBP and DBP control; the quality of evidence for the remaining interventions was too low. In the absence of original literature on prolonged interventions, only long-term BP lowering is currently recommended for LSM.

**Table 3:** Subgroups analysis of SBP and DBP based on prolonged intervention and different ethnic and cultural backgrounds (Asian, Caucasian).

	**SBP**				**DBP**			
**Comparison**	**No. of study**	**Weighted mean difference (95% CrI)**	**Certainty of evidence (CINEMA)**	**SUCRA (efficacy ranking)**	**No. of study**	**Weighted mean difference (95% CrI)**	**Certainty of evidence (CINEMA)**	**SUCRA (efficacy ranking)**
1**AE**								
Overall analysis	3	−10.65 (−17.12, −4.32)	⊕⊕⊕ O	6	3	−8.03 (−12.25, −3.69)	⊕⊕⊕⊕	4
12 months and above	0				0			
Asians	3	−10.62 (−17.39, −3.85)	⊕⊕ OO	6	3	−7.84 (−12.53, −3.1)	⊕⊕ OO	5
CaucAsianss	0				0			
**HIIT**								
Overall analysis	0	−12.46 (−22.42, −2.39)	⊕⊕ OO	4	0	−7.46 (−14.89, −0.13)	⊕⊕⊕ O	5
12 months and above	0				0			
Asians	0	−13.19 (−26.05, −0.39)	⊕ OOO	4	0	−9.03 (−18.16, 0.33)	⊕⊕ OO	4
CaucAsianss	0				0			
**RE**								
Overall analysis	3	−6.54 (−13.87, 0.71)	⊕ OOO	15	3	−6 (−11.24, −0.59)	⊕⊕⊕ O	7
12 months and above	1	−8.58 (−26.54, 9.56)	⊕ OOO	6	1	−7.84 (−19.66, 3.92)	⊕ OOO	3
Asians	1	−1.49 (−15.31, 12.57)	⊕ OOO	21	1	0.73 (−10.94, 12.52)	⊕ OOO	22
CaucAsianss	2	−8.74 (−14.66, −2.83)		2	2	−7.92 (−12.43, −3.47)		2
**Yoga**								
Overall analysis	1	−7.73 (−20.29, 4.44)	⊕ OOO	12	1	−4.6 (−12.63, 3.52)	⊕⊕ OO	12
12 months and above	0				0			
Asians	0	−7.73 (−20.92, 5.64)	⊕ OOO	13	0	−4.21 (−13.13, 4.58)	⊕ OOO	11
CaucAsianss	0				0			
**DASH**								
Overall analysis	2	−9.21 (−16.47, −1.67)	⊕⊕⊕ O	8	2	−5.81 (−10.8, −0.81)	⊕⊕⊕ O	8
12 months and above	1	−17.38 (−35.14, 0.23)	⊕⊕⊕ O	1	1	−8.8 (−19.6, 1.98)	⊕⊕ OO	2
Asians	2	−9.36 (−17.28, −1.32)	⊕⊕ OO	9	2	−6.06 (−11.51, −0.6)	⊕⊕ OO	7
CaucAsianss	0				0			
**TPM dietary habit**								
Overall analysis	1	−7.13(−19.32, 5.19)	⊕ OOO	14	1	−3.9 (−12.01, 4.29)	⊕ OOO	14
12 months and above	0				0			
Asians	0				0			
CaucAsianss	1	−7.06 (−15.18, 1.05)		3	1	−3.85 (−9.8, 2.07)		3
**Salt restriction**								
Overall analysis	3	−3.86 (−10.95, 3.15)	⊕ OOO	24	3	−2.18 (−6.91, 2.46)	⊕⊕ OO	21
12 months and above	1	0.3 (−17.18, 17.57)	⊕⊕ OO	13	1	0.07 (−10.11, 10.43)	⊕⊕ OO	13
Asians	0				0			
CaucAsianss	3	−3.16 (−8.01, 1.15)		6	3	−1.66 (−5.33, 1.52)		9
**Micronutrients**								
Overall analysis	3	−3.27 (−8.99, 2.61)	⊕⊕ OO	27	3	0.46 (−3.41, 4.37)	⊕⊕ OO	29
12 months and above	2	−0.32 (−12.67, 12.14)	⊕ OOO	14	2	0.01 (−7.15, 7.36)	⊕ OOO	14
Asians	0				0			
CaucAsianss	1	−0.31 (−5.68, 5.03)		11	1	0.07 (−3.82, 3.95)		12
**Food extract**								
Overall analysis	19	−4.27 (−7.24, −1.31)	⊕⊕⊕ O	23	18	−2.06 (−4.08, −0.06)	⊕⊕⊕ O	22
12 months and above	0				0			
Asians	7	−5.12 (−10.58, 0.22)	⊕⊕ OO	18	7	−3.31 (−6.99, 0.39)	⊕⊕ OO	12
CaucAsianss	10	−3.96 (−6.47, −1.51)		4	9	−1.88 (−3.78, 0.05)		8
**Medicinal herbs Taking**								
Overall analysis	17	−8.51 (−11.71, −5.33)	⊕⊕ OO	9	17	−5.24 (−7.42, −3.09)	⊕⊕ OO	10
12 months and above	3	−6.62 (−16.75, 3.45)	⊕ OOO	9	3	−3.35 (−9.18, 2.8)	⊕ OOO	7
Asians	8	−7.74 (−11.37, −4.12)	⊕ OOO	12	8	−4.69 (−7.13, −2.23)	⊕ OOO	10
CaucAsianss	1	−21.71 (−29.82, −13.61)		1	1	−10.68 (−16.55, −4.88)		1
**TCD bubble**								
Overall analysis	2	−13.44 (−22, −4.64)	⊕⊕ OO	3	2	−5.87 (−11.7, −0.08)	⊕⊕⊕ O	9
12 months and above	0				0			
Asians	1	−13.52 (−22.63, −4.11)	⊕⊕⊕ O	3	1	−5.65 (−11.89, 0.81)	⊕⊕ OO	8
CaucAsianss	0				0			
**EMG**								
Overall analysis	2	−5.6 (−14.46, 3.32)	⊕ OOO	17	2	−2.98 (−8.93, 3)	⊕ OOO	16
12 months and above	0				0			
Asians	2	−5.59 (−14.94, 3.83)	⊕ OOO	15	2	−2.96 (−9.57, 3.51)	⊕ OOO	14
CaucAsianss	0				0			
**Acupoint therapy**								
Overall analysis	7	−7.3 (−12.1, −2.57)	⊕ OOO	13	7	−2.69 (−5.86, 0.51)	⊕ OOO	18
12 months and above	1	−13.45 (−32.2, 5.09)	⊕ OOO	2	1	−6.74 (−17.42, 4.26)	⊕ OOO	4
Asians	4	−7.29 (−12.48, −2.21)	⊕⊕ OO	14	4	−2.5 (−5.98, 0.96)	⊕ OOO	16
CaucAsianss	0				0			
**Homeopathy**								
Overall analysis	1	−2.72 (−14.95, 9.27)	⊕ OOO	26	1	−1.58 (−9.68, 6.49)	⊕⊕ OO	23
12 months and above	0				0			
Asians	1	−2.73 (−16.08, 10.5)	⊕ OOO	20	1	−1.58 (−10.46, 7.31)	⊕ OOO	17
CaucAsianss	0				0			
**Lose weight**								
Overall analysis	1	−2.82 (−14.79, 9.11)	⊕ OOO	25	1	−2.2 (−9.9, 5.64)	⊕⊕ OO	20
12 months and above	1	−2.76 (−20.19, 14.68)	⊕ OOO	11	1	−2.27 (−12.65, 8.08)	⊕ OOO	9
Asians	0				0			
CaucAsianss	1	−2.96 (−10.46, 4.59)		8	1	−2.24 (−7.7, 3.22)		7
**MBSR**								
Overall analysis	2	−4.23 (−12.9, 4.44)	⊕ OOO	20	2	−0.71 (−6.49, 5.07)	⊕⊕ OO	27
12 months and above	0				0			
Asians	1	−8.46 (−22.21, 5.27)	⊕ OOO	10	1	−0.63 (−10.25, 8.96)	⊕ OOO	19
CaucAsianss	1	−0.51 (−8.1, 6.95)		10	1	−0.82 (−6.33, 4.72)		10
**LSM**								
Overall analysis	9	−5.77 (−8.44, −3.12)	⊕ OOO	18	9	−3.56 (−5.33, −1.79)	⊕ OOO	15
12 months and above	7	−7.36 (−12.59, −2.12)	⊕⊕⊕ O	7	7	−4.18 (−7.27, −1.07)	⊕⊕⊕ O	6
Asians	8	−5.79 (−8.81, −2.75)	⊕ OOO	17	8	−3.18 (−5.2, −1.14)	⊕ OOO	13
CaucAsianss	0	7.45 (−2.98, 17.93)		12	0	−2.7 (−12.1, 6.83)		6
**TCM constitution intervention**								
Overall analysis	4	−4.27 (−10.44, 1.8)	⊕ OOO	21	4	−1.54 (−5.56, 2.46)	⊕ OOO	24
12 months and above	3	−3.88 (−13.91, 5.98)	⊕ OOO	10	3	−0.8 (−6.71, 5.01)	⊕ OOO	11
Asians	4	−4.33 (−10.77, 2.23)	⊕ OOO	19	4	−1.54 (−5.91, 2.76)	⊕ OOO	18
CaucAsianss	0				0			
**Remote guided LSM**								
Overall analysis	5	−4.1 (−9.67, 1.35)	⊕ OOO	22	4	−0.99 (−4.92, 2.91)	⊕⊕ OO	26
12 months and above	3	−0.32 (−10.25, 10.03)	⊕ OOO	15	3	0.46 (−6.81, 7.76)	⊕ OOO	15
Asians	1	1.37 (−11.69, 14.3)	⊕ OOO	23	1	1.73 (−6.7, 10.45)	⊕ OOO	23
CaucAsianss	3	−2.72 (−7.17, 1.81)		9	3	−2.86 (−6.76, 1.23)		4
**AE** + **RE**								
Overall analysis	2	−8.23 (−16.72, 0.38)	⊕⊕⊕ O	10	2	−11.02 (−16.73, −5.21)	⊕⊕⊕⊕	2
12 months and above	0				0			
Asians	2	−8.22 (−17.43, 0.88)	⊕⊕ OO	11	2	−10.81 (−17.11, −4.58)	⊕⊕⊕⊕	2
CaucAsianss	0				0			
**LSM** + **drug**								
Overall analysis	2	−11.07 (−18.5, −3.49)	⊕⊕ OO	5	2	−10.08 (−14.93, −5.28)	⊕⊕⊕ O	3
12 months and above	2	−11.92 (−22.79, −0.81)	⊕⊕ OO	3	2	−10.44 (−16.77, −3.92)	⊕⊕ OO	1
Asians	2	−11.09 (−19.17, −2.9)	⊕⊕ OO	5	2	−9.95 (−15.32, −4.68)	⊕⊕⊕ O	3
CaucAsianss	0				0			
**Baduanjin** + **Acupoint**								
Overall analysis	1	−10.48 (−22.33, 1.6)	⊕ OOO	7	1	0.02 (−8.28, 8.35)	⊕ OOO	28
12 months and above	0	−10.55 (−28.24, 6.87)	⊕ OOO	5	0	−0.02 (−10.56, 10.67)	⊕ OOO	12
Asians	1	−10.58 (−23.65, 2.35)	⊕ OOO	7	1	−0.02 (−8.94, 8.96)	⊕ OOO	21
CaucAsianss	0				0			
**AE** + **Acupoint**								
Overall analysis	1	−1.42 (−13.45, 10.76)	⊕ OOO	29	1	−0.47 (−8.58, 7.51)	⊕⊕ OO	25
12 months and above	1	−1.29 (−18.71, 15.65)	⊕⊕ OO	12	1	−0.44 (−11.14, 10.12)	⊕⊕ OO	10
Asians	1	−1.37 (−14.46, 11.64)	⊕⊕ OO	22	1	−0.47 (−9.41, 8.3)	⊕⊕ OO	20
CaucAsianss	0				0			
**ARB**								
Overall analysis	6	−12.5 (−17.59, −7.54)	⊕⊕⊕ O	2	6	−6.42 (−9.69, −3.26)	⊕⊕ OO	6
12 months and above	5	−10.13 (−17.65, −2.47)	⊕⊕ OO	4	5	−4.45 (−8.95, 0.05)	⊕⊕ OO	5
Asians	3	−12.86 (−18.45, −7.48)	⊕⊕ OO	2	3	−6.56 (−10.24, −2.92)	⊕⊕ OO	6
CaucAsianss	0				0			
**Beta blocker**								
Overall analysis	2	−2.56 (−11.32, 6.31)	⊕ OOO	28	2	−2.42 (−8.09, 3.44)	⊕ OOO	19
12 months and above	0				0			
Asians	0				0			
CaucAsianss	2	−3.01 (−8.66, 3.07)		7	2	−2.89 (−6.86, 1.48)		5
**CCB**								
Overall analysis	1	−23.67 (−36.34, −11)	⊕⊕ OO	1	1	−12.44 (−20.81, −4.04)	⊕⊕⊕ O	1
12 months and above	0				0			
Asians	0	−23.63 (−37.4, −10.02)	⊕⊕ OO	1	0	−12.06 (−21.5, −2.95)	⊕⊕ OO	1
CaucAsianss	0				0			
**Diuretic**								
Overall analysis	2	−7.92 (−14.44, −1.24)	⊕⊕ OO	11	2	−3.95 (−8.24, 0.38)	⊕⊕ OO	13
12 months and above	1	−7.02 (−18.91, 4.82)	⊕ OOO	8	1	−3.11 (−10.22, 3.84)	⊕ OOO	8
Asians	1	−9.81 (−18.31, −1.41)	⊕ OOO	8	1	−5.13 (−10.68, 0.44)	⊕⊕ OO	9
CaucAsianss	1	−3.4 (−11.12, 4.28)		5	1	−0.59 (−6.01, 4.84)		11
**Statins**								
Overall analysis	3	−5.32 (−13.06, 2.41)	⊕ OOO	19	3	−2.85 (−8.27, 2.53)	⊕⊕ OO	17
12 months and above	0	−7.02 (−18.91, 4.82)	⊕ OOO	8	0	−3.11 (−10.22, 3.84)	⊕ OOO	8
Asians	3	−5.35 (−13.64, 2.97)	⊕⊕ OO	16	3	−2.87 (−8.66, 2.93)	⊕ OOO	15
CaucAsianss	0				0			
**Allopurinol**								
Overall analysis	1	−5.95 (−19.53, 7.46)	⊕ OOO	16	1	−5.03 (−13.31, 3.32)	⊕ OOO	11
12 months and above	0				0			
Asians	0				0			
CaucAsianss	0				0			

CI, confidence interval; RR, risk ratio; SUCRA, surface under the cumulative ranking; AE, aerobic exercise; HIIT, high-intensity interval training; RE, resistance exercise; DASH, dietary approaches to stop hypertension; TPM, Traditional Persian Medicine; TCD, traditional Chinese drug; EMG, electromyographic; MBSR, mindfulness-based stress reduction; LSM, lifestyle modification; TCM, Traditional Chinese Medicine; ARB, angiotensin II receptor blockers; ACE, angiotensin-converting enzyme inhibitors; CCB, calcium channel blockers.

⊕ ⊕ ⊕ O, moderate quality.

⊕ ⊕ OO, low quality.

⊕ OOO, very low quality.

Based on differences in the ethnic and cultural backgrounds of the included populations, 72 studies from yellow and 29 studies from white populations were analyzed by subgroup (Table 3) and sensitivity analysis (Supplementary Table 17), including BP indicators (the three studies based on black populations could not be subjected to NMA construction). Analysis of 23 studies from Asian-based data showed that the TCD bubble was significant in reducing SBP; AE combined with RE, and LSM combined with drug were significant in reducing DBP. A total of 12 interventions in Caucasians, Medicinal herbs taking and RE had reduced SBP and DBP significantly. Food extract was effective in reducing DBP.

### 3.6. Inconsistency tests

There is a closed loop of outcome indicators for BP and HT progression in this paper, and inconsistency analysis was performed using the node-splitting method (Supplementary Figure 3). As the control group included in the literature included LSM, usual care and no intervention, some inconsistency (p<0.05) emerged between direct and indirect comparisons of medicinal herbs taking, LSM and emote-guided LSM in the analysis of SBP; and medicinal herbs taking and LSM in the analysis of DBP. This result will be explained in CINeMA and in the discussion. Otherwise, the difference between the direct and indirect evidence results for the intervention programmes was not statistically significant, indicating good agreement between the direct and indirect comparisons.

### 3.7. CINeMA quality of evidence grading results

The quality of evidence for the primary outcomes measured by CINeMA ranged from high to very low (Supplementary Tables 8–11), with the quality of evidence obtained for intervention programmes with SBP as an outcome ranging from moderate to very low, for intervention programmes involving a reduction in DBP ranging from high to very low, and for all HT progression and cardio-renal death outcomes being low or very low. The lower overall quality of the body of evidence is mainly due to problems with the Imprecision and Heterogeneity of the studies. To ensure the credibility of the results, only high or moderate quality evidence is recommended for analysis in this paper.

## 4. Discussion

This study used the NMA to summarize the effectiveness of 30 pharmacological and non-pharmacological interventions in reducing BP and delaying progression to adverse outcomes such as HT and cardiac, cerebral, renal and mortality outcomes in people with PHT to make recommendations for interventions, with a combination of SUCRA ranking and CINeMA evidence quality.

In terms of BP reduction, ARB, AE, DASH, and food extracts reduce SBP with moderate quality evidence support; in terms of DBP reduction, AE combined with RE and exercise measures for AE are preferentially recommended with high quality evidence levels. When the length of intervention was extended beyond 12 months, only LSM retained a moderate quality of evidence for BP reduction in terms of SBP and DBP reduction. At the same time, there were significant differences in priority interventions based on ethnicity. For Asians, TCD bubble, AE combined with RE and LSM combined with drug are first recommended to reduce SBP and DBP respectively; for Caucasians, RE, Medicinal herbs taking to reduce BP, or Food extract to reduce SBP are recommended. No measures are recommended for slowing the progression of HT, prevention of all organ lesions and mortality outcomes, because of the low to very low quality level of evidence.

ARBs (candesartan, irbesartan, and telmisartan), one of the first-line antihypertensive drugs for initial use (19), have been shown to be less effective in the HT population (20), but have shown better efficacy in the PHT population. ARB also has a lower incidence of adverse events after discontinuation than all antihypertensive drugs and has the advantage of maintaining stable BP (21), preventing cardiovascular events caused by rapid changes in BP. Experimental data suggest that ARBs are better at improving arterial stiffness (22) and that administration early in the course of the disease results in a stronger effect of this vascular change-mediated hypotension (23). CCB (amlodipine) also achieved good results due to its sodium-independent antihypertensive effect in response to the high salt intake dietary preferences of East Asian populations (24). In the overall analysis, drugs achieved better efficacy and preferred treatment ranking, but their antihypertensive effect became unclear when treatment was prolonged or when analyzed across ethnic groups. Drug regimens are not recommended for people with no other co-morbidities and whose BP is not close to the threshold, and when PHT requires drug treatment, treatment with ARBs may be preferred or, in Asian populations, with CCBs.

LSM is a comprehensive non-pharmacological intervention to change physically and mentally unhealthy behaviors and habits (25) and is mostly considered in this paper in terms of dietary modification (DASH, salt restriction), management of tobacco, alcohol and increased activity. It was not recommended in the mixed comparison because of the low quality of the evidence, but as the duration of the intervention increased and the number of interventions with LSM as a control group decreased, LSM became the only intervention effective in reducing BP. And it may also have a positive effect in slowing progression to HT. This is consistent with the results of the current guidelines.

Recently the use of remote based tools (26) (telephone, SMS, and web) for LSM interventions has been gaining ground, improving patient compliance while being less time and location dependent. The remote LSM interventions included in this paper also need to be of sufficient duration (12 months) and intensity (27) to increase their appeal and thus produce meaningful outcomes.

Guidelines and studies in a range of regions have demonstrated the antihypertensive effect of physical activity (28, 29). The same positive efficacy was obtained in the PHT patients in this paper. AE, AE combined with RE have moderate to high quality levels of evidence in reducing SBP and DBP, making them the best interventions recommended here. Meanwhile, RE and HIIT have good evidence for reducing DBP. However, due to the lack of support from trials >12 months, this paper cannot explore the long-term pressure control effects of exercise therapy, which is an important part of the next step that needs to be urgently achieved. Moreover, AE combined with RE had moderate quality evidence of DBP reduction in Asian populations, and RE may also have an effect in reducing BP in Caucasians. The cumulative effects of physical activity, as perceived through its effects on sympathetic activity, enhancement of endothelial function and reduction of oxidative stress, contribute to the prevention and treatment of hypertension (30).

The benefits of AE in modulating cardiovascular risk factors are widely recognized, and the moderate to high intensity AE (over 20 min three times a week to achieve 50–85% HRmax) included in this paper resulted in a −10.65/−8.03 mmHg reduction in BP over the course of 5–24 weeks of exercise. The remaining exercise modalities yielded more definite gains in DBP reduction, with RE as a complementary therapy to AE having the best BP reduction in combination with AE and, to a lesser extent, alone. HIIT has the advantage of being more time efficient in lowering BP and is suitable for young people who are short of time (31). In addition to its effectiveness in controlling BP, another potential benefit of physical activity is weight loss (29), which is often the other non-pharmacological intervention recommended for PHT (32). With a weight loss of 10 kg, BP can be reduced by 5–20 mmHg. The combined benefits of lowering BP and weight loss after physical activity may help to further reduce or prevent elevated levels of pressure (33).

Data from the Global Burden of Disease Group (34) suggests that an unhealthy diet is a major risk factor for premature death and disability. To control BP, the guidelines suggest dietary recommendations that should be adopted by people with hypertension: increase the intake of fresh fruit and vegetables, low-fat dairy products and reduce the intake of sodium. The DASH intervention fits the above components and has shown promising BP-lowering effects in the analysis and is supported by evidence in Asians. The lack of efficacy of salt restriction as a stand-alone intervention is consistent with previous studies in the PHT population (35, 36) and may also be related to the different criteria in this paper, whereas increases in other micronutrients (potassium and magnesium) may reduce SBP. Foods containing flavanols, polyphenols, and anthocyanins promote vasodilation by increasing nitric oxide utilization (37, 38) (grape seed, and cocoa) and reducing oxidative stress (39, 40) (green tea and roselle). However, the short duration of intervention (4–24 weeks) and the small sample size of each individual extract, combined with access and economic costs can only be used as a complementary programme.

Traditional medicine in various countries has been shown to be effective in reducing BP. TCD bubble produces moderate quality evidence recommendations in reducing DBP through a decoction of one or more herbal formulations that allow the medicine to be absorbed through the skin of the foot and stimulate acupuncture points on the foot through a warming effect.

## 5. Strengths and limitations

This is the first NMA analysis of a PHT population that uses direct vs. indirect comparisons to provide reliable estimates of outcome indicators. This paper provides an extensive search of the database and does not restrict interventions or language to include more RCTs for comparison. Moreover, intervention studies of more than 4 weeks were included, with an overall considerable sample size to interpret on the four outcome indicators. At the same time, a subgroup analysis of BP indicators in people from different ethnic and cultural backgrounds, over 12 months of intervention, provides more targeted advice.

The main limitation comes from the protocols and number of studies included in the literature. Studies in subgroup analyses need to be conducted over a longer period of time and in different ethnic contexts to produce more relevant results.

## 6. Conclusions

The main findings of this study suggest that AE, an exercise regimen of AE combined with RE and DASH are preferentially recommended for the PHT population as moderate to high quality evidence for BP lowering, and LSM is recommended as a long-term BP control regimen for intervention; on top of this, the addition of TCD bubble for SBP lowering in yellow populations and RE as a possible means of BP lowering in the Caucasian population. Long-term interventions in different cultural contexts will also need to be added in the future, with attention to the impact of interventions on final outcome indicators.

## Data availability statement

The original contributions presented in the study are included in the article/Supplementary material, further inquiries can be directed to the corresponding author.

## Author contributions

WL contributed to the conception of the article, searched and analyzed the data, and wrote the original article. XW contributed in analyzing and checking the data and revising the original article. HL contributed to conceiving the article, analyzing the data, and revising the original text. CW and YW provide suggestions for screening the literature and extracting data. HX and JL made key suggestions and gave important input in revising the original article. All authors contributed to the critical revision of important intellectual content of the article and read and approved the final version of this article.
