# Supplementary material for: Interventions for reducing blood pressure in prehypertension: A meta-analysis
Source: Front Public Health. 2023 Mar 23;11:1139617. doi: 10.3389/fpubh.2023.1139617 (PMC10078829; doi:10.3389/fpubh.2023.1139617)
Supplement: Supplementary file 1 [file Data_Sheet_1.docx]

**Supplementary materials**

Supplementary Table 1. PRISMA checklist

Supplementary Table 2. Full Search Strategy

Supplementary Table 3. Basic characteristics of the included studies

Supplementary Table 4. Model DIC values (SBP, DBP)

Supplementary Table 5. Risk of bias assessment for inclusion in studies

Supplementary Table 6. Egger test for BP

Supplementary Table 7. Network meta-analysis of the antihypertensive effect of SBP and DBP

Supplementary Table 8. Network meta-analysis of HT progression and cardiac, cerebral, renal and mortality outcomes

Supplementary Table 9. SCURA scores for interventions

Supplementary Table 10. CINeMA results for SBP

Supplementary Table 11. CINeMA results for DBP

Supplementary Table 12. CINeMA results for HT progression

Supplementary Table 13. CINeMA results for cardiac, cerebral, renal and mortality outcomes

Supplementary Table 14. Summary of findings on the efficacy of various interventions for the progression rate of HT

Supplementary Table 15. Summary of findings on the efficacy of various interventions for the progression rate of cardiac, cerebral and renal outcomes

Supplementary Table 16. Sensitivity analysis based on omitted medium and low quality studies

Supplementary Table 17. Subgroups and sensitivity analysis of SBP and DBP based on prolonged intervention and different ethnic and cultural backgrounds (Asian, Caucasian)

Supplementary Figure 1. Publication bias for (a)SBP, (b)DBP and (c)HT progression rates

Supplementary Figure 2. SUCRA plots for (a)SBP, (b)DBP, (c)HT progression rates (d)cardiac, renal and mortality outcome incidence

Supplementary Figure 3. Inconsistency plots for (a)SBP, (b)DBP and (c)HT progression rates

Supplementary Table 1. PRISMA checklist

| **Section and Topic** | **Item #** | **Checklist item** | **Location where item is reported** |
| --- | --- | --- | --- |
| **TITLE** | | |  |
| Title | 1 | Identify the report as a systematic review. | 1 |
| **ABSTRACT** | | |  |
| Abstract | 2 | See the PRISMA 2020 for Abstracts checklist. | 1-2 |
| **INTRODUCTION** | | |  |
| Rationale | 3 | Describe the rationale for the review in the context of existing knowledge. | 3-4 |
| Objectives | 4 | Provide an explicit statement of the objective(s) or question(s) the review addresses. | 4 |
| **METHODS** | | |  |
| Eligibility criteria | 5 | Specify the inclusion and exclusion criteria for the review and how studies were grouped for the syntheses. | 5 |
| Information sources | 6 | Specify all databases, registers, websites, organisations, reference lists and other sources searched or consulted to identify studies. Specify the date when each source was last searched or consulted. | 5 |
| Search strategy | 7 | Present the full search strategies for all databases, registers and websites, including any filters and limits used. | 5 |
| Selection process | 8 | Specify the methods used to decide whether a study met the inclusion criteria of the review, including how many reviewers screened each record and each report retrieved, whether they worked independently, and if applicable, details of automation tools used in the process. | 5-6 |
| Data collection process | 9 | Specify the methods used to collect data from reports, including how many reviewers collected data from each report, whether they worked independently, any processes for obtaining or confirming data from study investigators, and if applicable, details of automation tools used in the process. | 5-6 |
| Data items | 10a | List and define all outcomes for which data were sought. Specify whether all results that were compatible with each outcome domain in each study were sought (e.g. for all measures, time points, analyses), and if not, the methods used to decide which results to collect. | 5-6 |
|  | 10b | List and define all other variables for which data were sought (e.g. participant and intervention characteristics, funding sources). Describe any assumptions made about any missing or unclear information. | 5-6 |
| Study risk of bias assessment | 11 | Specify the methods used to assess risk of bias in the included studies, including details of the tool(s) used, how many reviewers assessed each study and whether they worked independently, and if applicable, details of automation tools used in the process. | 6 |
| Effect measures | 12 | Specify for each outcome the effect measure(s) (e.g. risk ratio, mean difference) used in the synthesis or presentation of results. | 6-7 |
| Synthesis methods | 13a | Describe the processes used to decide which studies were eligible for each synthesis (e.g. tabulating the study intervention characteristics and comparing against the planned groups for each synthesis (item #5)). | 6-8 |
|  | 13b | Describe any methods required to prepare the data for presentation or synthesis, such as handling of missing summary statistics, or data conversions. | 6 |
|  | 13c | Describe any methods used to tabulate or visually display results of individual studies and syntheses. | 6-7 |
|  | 13d | Describe any methods used to synthesize results and provide a rationale for the choice(s). If meta-analysis was performed, describe the model(s), method(s) to identify the presence and extent of statistical heterogeneity, and software package(s) used. | 7-8 |
|  | 13e | Describe any methods used to explore possible causes of heterogeneity among study results (e.g. subgroup analysis, meta-regression). | 7-8 |
|  | 13f | Describe any sensitivity analyses conducted to assess robustness of the synthesized results. | 7 |
| Reporting bias assessment | 14 | Describe any methods used to assess risk of bias due to missing results in a synthesis (arising from reporting biases). | 6 |
| Certainty assessment | 15 | Describe any methods used to assess certainty (or confidence) in the body of evidence for an outcome. | 7 |
| **RESULTS** | | |  |
| Study selection | 16a | Describe the results of the search and selection process, from the number of records identified in the search to the number of studies included in the review, ideally using a flow diagram. | 8 |
|  | 16b | Cite studies that might appear to meet the inclusion criteria, but which were excluded, and explain why they were excluded. | 8 |
| Study characteristics | 17 | Cite each included study and present its characteristics. | 9-10 |
| Risk of bias in studies | 18 | Present assessments of risk of bias for each included study. | 10 |
| Results of individual studies | 19 | For all outcomes, present, for each study: (a) summary statistics for each group (where appropriate) and (b) an effect estimate and its precision (e.g. confidence/credible interval), ideally using structured tables or plots. | 10-12 |
| Results of syntheses | 20a | For each synthesis, briefly summarise the characteristics and risk of bias among contributing studies. | 10-12 |
|  | 20b | Present results of all statistical syntheses conducted. If meta-analysis was done, present for each the summary estimate and its precision (e.g. confidence/credible interval) and measures of statistical heterogeneity. If comparing groups, describe the direction of the effect. | 10-14 |
|  | 20c | Present results of all investigations of possible causes of heterogeneity among study results. | 13-14 |
|  | 20d | Present results of all sensitivity analyses conducted to assess the robustness of the synthesized results. | 13-14 |
| Reporting biases | 21 | Present assessments of risk of bias due to missing results (arising from reporting biases) for each synthesis assessed. | 11-14 |
| Certainty of evidence | 22 | Present assessments of certainty (or confidence) in the body of evidence for each outcome assessed. | 11-14 |
| **DISCUSSION** | | |  |
| Discussion | 23a | Provide a general interpretation of the results in the context of other evidence. | 14-19 |
|  | 23b | Discuss any limitations of the evidence included in the review. | 18-19 |
|  | 23c | Discuss any limitations of the review processes used. | 18-19 |
|  | 23d | Discuss implications of the results for practice, policy, and future research. | 18-19 |
| **OTHER INFORMATION** | | |  |
| Registration and protocol | 24a | Provide registration information for the review, including register name and registration number, or state that the review was not registered. | 4 |
|  | 24b | Indicate where the review protocol can be accessed, or state that a protocol was not prepared. | 4 |
|  | 24c | Describe and explain any amendments to information provided at registration or in the protocol. | 5-6 |
| Support | 25 | Describe sources of financial or non-financial support for the review, and the role of the funders or sponsors in the review. | 1 |
| Competing interests | 26 | Declare any competing interests of review authors. | 1 |
| Availability of data, code and other materials | 27 | Report which of the following are publicly available and where they can be found: template data collection forms; data extracted from included studies; data used for all analyses; analytic code; any other materials used in the review. | 7 |

*From:*  Page MJ, McKenzie JE, Bossuyt PM, Boutron I, Hoffmann TC, Mulrow CD, et al. The PRISMA 2020 statement: an updated guideline for reporting systematic reviews. BMJ 2021;372:n71. doi: 10.1136/bmj.n71

For more information, visit: <http://www.prisma-statement.org/>

Supplementary Table 2. Full Search Strategy

| **Database** | **Search formula** |
| --- | --- |
| **PubMed** | (((((prehypertensi*[Title/Abstract]) OR (pre hypertension[Title/Abstract])) OR (pre-hypertension[Title/Abstract])) OR (pre-HTN[Title/Abstract])) OR (high normal blood pressure[Title/Abstract])) OR (high normal BP[Title/Abstract]) |
| **Embase** | (prehypertensi*:ti,ab,kw OR 'pre hypertension':ti,ab,kw OR prehypertension:ti,ab,kw OR 'pre htn':ti,ab,kw OR 'high normal blood pressure':ti,ab,kw OR 'high normal bp':ti,ab,kw) AND ('randomized controlled trial'/exp OR 'clinical article'/de OR 'clinical trial'/de OR 'cohort analysis'/de OR 'comparative study'/de OR 'controlled clinical trial'/de OR 'controlled study'/de OR 'double blind procedure'/de OR 'human'/de OR 'major clinical study'/de OR 'prospective study'/de OR 'randomized controlled trial topic'/de OR 'retrospective study'/de) |
| **Web of Science** | #1 (((((TS=(prehypertensi*)) OR TS=(pre hypertension)) OR TS=(pre-hypertension)) OR TS=(pre-HTN)) OR TS=(high normal blood pressure)) OR TS=(high normal BP) #2 TS=(Randomized Controlled Trial*) OR TS=(Pragmatic Clinical Trial*) OR TS=(Randomized) #1 AND #2 |
| **Cochrane Library** | prehypertensi* OR pre hypertension OR pre-hypertension OR pre-HTN OR high normal blood pressure OR TS high normal BP |
| **China National Knowledge Infrastructure** | TKA='正常高值血压' + '高血压前期' + '血压正常高值' + '临界高血压' AND TKA='随机' |
| **Wanfang Data** | 题名或关键词:("正常高值血压" OR "高血压前期" OR "血压正常高值" OR "临界高血压") and 题名或关键词:( "随机" ) |
| **China Science and Technology Journal Database (VIP)** | M=(正常高值血压OR高血压前期OR血压正常高值OR临界高血压) AND M=(随机) |

Supplementary Table 3. Basic characteristics of the included studies

| First author,  year | Country | Types of pre-HT | BP inclusion range | N (I,C) | Gender (I,C) | Mean age (year) | Interventions | Specific Intervention Methods | Duration Weeks | BP measurement method,  frequency |
| --- | --- | --- | --- | --- | --- | --- | --- | --- | --- | --- |
| Xuan Xiao, 2014^1^ | China | Adults | SBP:120-139 DBP:80-89 | 120 (40,40,40) | 48,72 | 45 | AE ＋RE AE No intervention | AE: brisk walking or jogging 5 times/week (30 min each), reaching 50%-65% HRmax. RE: elastic band training 5 times/week, 20 min each time. | 24 | NA |
| Piyapong Prasertsri, 2018^2^ | Thailand | Age 60 - 80 years | SBP:120-139 DBP:80-89 | 50 (25,25) | 11,39 | 66.9 | Arm swing exercise No intervention | 3 times a week, perform an arm swing exercise training program (30 min/day) or no intervention. | 12 | Electronic, 3 |
| Qin Yang, 2020^3^ | China | Middle aged women | SBP:120-139 DBP:80-89 | 18 (9,9) | 0,18 | 57.44 NR | Square dance No intervention | 4 times a week, perform square dance (40 min each), reaching 60%-80% HRmax or no intervention. | 8 | Mercury, NA |
| Anil T John, 2022^4^ | Malaysia | Young Adults | SBP:120-139 DBP:80-89 | 32 (12,10,10) | 22,10 | 20 | HIIT CMT or DASH | 3 times a week, running (20 min each) including a 1:4 minute rest ratio to achieve 80%-85% HRmax or continuous running (20 min each) at 40%-60% HR max or DASH. | 5 | Electronic, 3 |
| Benjamin C, 2016^5^ | America | Adults | SBP≥120 DBP≥80 | 12 (7,5) | 3,12 | 33.3 | HIIT AE or DASH | HIIT:cycling (20 min each) including a 1:1 minute rest ratio ,4 days/week to 60% HRmax. AE:continuous cycling (30 min each) , 4 days/week to 40% HR max. | 8 | Electronic, 3 |
| Darren T Beck, 2013^6^ | America | Youth | SBP:120-139 DBP:80-89 | 43 (15,13,15) | 39,19 | 21.13 | Resistance training  Endurance training  Usual care | Three days a week, perform resistance training (1 hour)  or endurance training (1 hour)  or avoid exercise. | 8 | Electronic, 3 |
| Zhonghao Liu, 2021^7^ | China | Male students | SBP:120-139 DBP:80-89 | 32 (8,8,8,8) | 32,0 | 19-21 | RE Low pressure BFR-training + RT Medium pressure BFR-training + RT No intervention | Four days a week, perform upper and lower body RT (20 min/repetition) or RT + BFR-training (upper and lower extremities: 60/160 mmHg) or RT + BFR-training (upper and lower extremities: 90/ 220 mmHg)  or no intervention. | 4 | Electronic, NA |
| Jamie M. O’Driscoll, 2022^8^ | UK | Male | SBP:120-139 DBP:80-89 | 24 (12,12) | 24,0 | 45 | IRT No intervention | During the 12 months, performed 3 weekly wall squat IRT sessions at 95% HR max or no intervention. | 48 | Electronic, 3 |
| Thiyagarajan,  2015^9^ | India | Adults | SBP:120-139 DBP:80-89 | 100 (51,49) | 62,38 | 43.3 | Yoga+LSM LSM | 3 days weekly,  perform 45 min yoga practise | 12 | NA, 2 |
| Cuihong Zheng, 2013^10^ | China | Adults | SBP:120-139 DBP:80-89 | 60 (30,30) | 30,30 | 30-85 | DASH Usual care | Everyday, implementing the DASH diet; or usual care | 52 | NA |
| Jiawei Wang, 2020^11^ | China | Herdsmen | SBP:120-139 DBP:80-89 | 272 (132,140) | 146,126 | 48.89 | DASH Usual care | Everyday, implementing the DASH diet; or usual care | 36 | NA |
| Hossein abolhosseini, 2021^12^ | Iran | Adults | SBP:120-139 DBP:80-89 | 84 (42,42) | 50,34 | 44.38 | Persian medical dietary habits No intervention | Adjust the diet according to Persian medical eating habits and questionnaires or no intervention. | 4 | Electronic, 3 |
| HPT Research Group, 1990^13^ | America | Adults | DBP:78-89 | 841(125,196, 129,195,196) | 549,292 | 38.6 | Reduced calories Reduced sodium Reduced sodium and calories Reduced sodium and increased potassium No intervention | Provide some frequency of counselling aimed at achieving and sustaining the desired dietary changes bring body weight to desirable body weight or urine sodium excretion ≤70 mmol/d  or urine potassium excretion ≤100 mmol/d. | 156 | Electronic, 2 |
| The TOHP Research Group,1992a ^14^ | America | Adults | DBP:80-89 | 744 (327,417) | 531,213 | 30-54 | Salt restriction Usual care | Everyday, intake sodium (80 mmol)  or LSM. | 24 | Hawksleyrandom-zerosphygmomanometer, 3 |
| François-André Allaert, 2017^15^ | France | Adults | SBP:130-139 DBP:85-89 | 41 (22,19) | 21,20 | 51 | Symbiosal salt Table salt | Everyday, symbiosal salt (NaCl + Chitosan 3%)  or table salt NaCl (< 3g) . | 8 | Electronic, 3 |
| The TOHP Research Group, 1992 d^14^ | America | Adults | DBP:80-89 | 698 (227,237,234) | 479,219 | 25-49 | Magnesium  Calcium  Placebo | Everyday, intake magnesium (15 mmol or 360 mg)  or calcium (25 mmol or 1.0 g)  or placebo. | 72 | Hawksleyrandom-zerosphygmomanometer, 3 |
| Mariana Rodríguez-Ramírez, 2017^16^ | Mexico | Adults | SBP:120-139 DBP:80-89 | 36 (18,18) | 15,21 | 51.75 | Magnesium Placebo | Everyday, elemental magnesium intake 360 mg or placebo. | 16 | Mercury, 3 |
| The TOHP Research Group, 1992 e^14^ | America | Adults | DBP:80-89 | 528 (178,175,175) | 379,149 | 25-49 | Potassium  Fish oil  Placebo | Everyday,  intake potassium (60 mmol or 4.5 g)  or fish oil (6 g)  or placebo. | 24 | Hawksleyrandom-zerosphygmomanometer, 3 |
| Jenny A Cadée, 2007^17^ | The Netherlands | Adults | SBP:120-139 DBP:80-89 | 48 (24,24) | 10,38 | 55.85 | C12 Peptide Placebo | Everyday, intake C12 Peptide 3.8 g or placebo. | 4 | Mercury, 3 |
| Karin Ried, 2009^18^ | Australia | Adults | SBP:120-139 DBP:80-89 | 36 (11,15,10) | 19,17 | 52.33 | Chocolate Tomato Placebo | Everyday, intake polyphenol 750 mg  or lycopene 15 mg or placebo. | 8 | Electronic, 4 |
| Arturo Figueroa, 2011^19^ | America | Adults | SBP<140 DBP<90 | 9 (4,5) | 4,5 | 54 | Watermelon Placebo | Twice a day, intake watermelon supplementation (L-citrulline/L-arginine: 1.35 g/0.65 g)  or placebo. | 6 | high-fidelitytonometer, 2 |
| Jatuporn Wichitsranoi, 2011^20^ | Thailand | Adults | SBP:120-139 DBP:80-89 | 30 (15,15) | 22,8 | 49.8 | Black sesame meal  Placebo | Everyday, intake black sesame meal capsules (2.52 g)  or placebo. | 4 | Electronic, 3 |
| Biswas OS, 2015^21^ | America | Adults | SBP:120-139 DBP:80-89 | 29 (17,12) | 15,14 | 40.66 | NO lozenge Placebo | Everyday,Twice a day. intake NO lozenge  or placebo.twice a day daily | 30 days | RivaRocci, 2 |
| Stacey Lockyer, 2015^22^ | New Zealand | Adults | SBP:121-140 DBP:81-90 | 61 (31,30) | 60,0 | 45 | Olive leaf extract Placebo | Every day, intake olive leaf extract(136 mg oleuropein; 6 mg hydroxytyrosol)  or placebo. | 6 | Electronic, 3 |
| Han Saem Jeong, 2016^23^ | South Korea | Adults | SBP:130-139 DBP:85-89 | 45 (15,15,15) | 24,21 | 57.2 | High-dose black raspberry Moderate-dose black raspberry Placebo | Everyday, intake high-dose black raspberry (2500 mg/d)  or moderate-dose black raspberry (1500 mg/d)  or placebo. | 8 | Electronic, NA |
| Magdalena Madero, 2015 a^24^ | Mexico | Overweight or obese Adults with a history of high fructose consumption | SBP:120-139 DBP:80-89 | 72 (34,38) | 44,38 | 46 | Low fructose diet  Baseline diet | Everyday, perform low fructose (< 20g/d) + low sodium diet or low sodium diet. | 4 | Mercury, 3 |
| Eunyoung Park, 2016^25^ | America | Adults | SBP:120-139 DBP:80-89 | 29 (12,17) | 15,14 | 42.86 | GSE Placebo | Everyday, intake GSE juice (300mg)  or 0 mg. | 6 | Dynamic |
| Qianqian Sun, 2016^26^ | China | Adults | SBP:120-139 DBP:80-89 | 111 (56,55) | 46,65 | 56.49 | Taurine Placebo | Everyday, intake taurine supplementation (1.6 g)  or placebo | 12 | Mercury, 3 |
| F Pelliccia, 2017^27^ | Italy | Adults | SBP:130-139 DBP:85-89 | 176 (88,88) | 103,73 | 52 | Dietary supplement Placebo | Once or twice daily, dietary supplement capsule (250 mg Allium sativum , 250 mg Crataegus , 150 mg Ortosiphon and 125 mg Hibiscus) or placebo. | 12 | Dynamic |
| Lívia de P. Nogueira MD, 2017^28^ | Brazil | Obesity female | SBP:120-139 DBP:80-89 | 20 (20,20) | 0,20 | 41.1 | GTE Placebo | Two groups were given 500 mg/d GTE (260 mg polyphenols) or placebo for 4 weeks, and 2 weeks of washout period was used between the two groups. | 4 | Dynamic |
| Hye-Jeong Park, 2019^29^ | South Korea | Adults | SBP:130-139 DBP:80-89 | 76 (29,24,23) | 51,25 | 30.35 | Policosanol 20 mg Policosanol 10 mg Placebo | Everyday, intake policosanol (10 mg or 20 mg)  or placebo. | 12 | Mercury, Electronic, SphygmoCor, 3 |
| Tamami Odai, 2019^30^ | Japan | Adults | SBP:130-139 DBP:85-89 | 30 (10,10,10) | 6,24 | 53.7 | High-dose GSE Low-dose GSE Placebo | Everyday, intake high or low dose (400 mg/day or 200 mg/day) of GSE  or placebo. | 12 | vascularscreeningsystem |
| Haidar Alatas, 2020^31^ | Indonesia | Adults | SBP:120-139 DBP:80-89 | 143 (71,72) | 107,36 | 45.4 | Soursop  No intervention | Everyday, intake ripe seedless soursop fruit juice (200g)  or no intervention. | 12 | Electronic, 3 |
| Cindy MT van der Avoort, 2021^32^ | The Netherlands | Adults | SBP:130-139 DBP:85-89 | 77 (25,26,26) | 51,26 | 65 | Nitrate-rich vegetable consumption  Beetroot juice supplementation No intervention | Everyday, intake nitrate-rich vegetables (350-400 mg nitrate)  or beetroot juice supplementation (400 mg nitrate)  or no intervention. | 12 | Dynamic |
| Cécile Vors, 2021^33^ | Canada | Adults | SBP:120-139 DBP<90 | 73 (36,37) | 31,42 | 49 | Polyphenols+L-citrulline  Placebo | Everyday, intake polyphenol (548 mg) + L- citrulline supplement (2 g)  or placebo. | 6 | Dynamic |
| Fenlin Niu, 2012^34^ | China | Adults | SBP:120-139 DBP:80-89 | 38(18,20) | 30,8 | 49.9 | Watermelon Placebo | Twice a day, intake L-citrulline (1.35 g/d) + usual care or placebo + usual care. | 8 | Mercury, 3 |
| Tae Woong Cha, 2016^35^ | South Korea | Adults | SBP:120-139 DBP:80-89 | 62 (31,31) | 50,12 | 42.05 | Red ginseng Placebo | Everyday, intake red ginseng (5g)  or placebo. | 12 | Electronic, 3 |
| You Kyoung Shin, 2019^36^ | South Korea | Adults | SBP:120-139 DBP:80-89 | 76 (39,37) | 60,16 | 29.45 | CL extract Placebo | Everyday, intake CL extract (1000 mg)  or placebo. | 6 | NA |
| Lei Shen, 2021^37^ | South Korea | Adults | SBP:120-139 DBP:80-89 | 88 (44,44) | 50,38 | 42.63 | Bokbunja Placebo | Everyday, intake Bokbunja (900 mg/day)  or placebo. | 8 | Electronic, 2 |
| Mansoor Ahmad Siddiqui, 2021^38^ | India | Adults | SBP:120-139 DBP:80-89 | 40 (20,20) | 27,13 | 41.48 | Tribulus terrestris Placebo | Everyday, intake Tribulus terrestris (6g)  or placebo. | 8 | NA |
| Hossein Beyranvand, 2022^39^ | Iran | Adults | SBP:120-139 DBP:80-89 | 75 (37,38) | 37,45 | 49.56 | Pinger Placebo | Everyday, intake gingerol (26 mg)  or placebo. | 8 | Mercury, NA |
| Shuangping Wu, 2014^40^ | China | Adults | SBP:120-139 DBP:80-89 | 100 (50,50) | 61,39 | 45.65 | Compound Zhenzhu Jiangya capsule + LSM LSM | Every day, intake 12 capsules of compounded Zhenzhu Jiangya capsule and LSM or LSM. | 30 days | Mercury, 3 |
| Hui Wu, 2011^41^ | China | Adults | SBP:130-139 DBP:80-89 | 68 (35,33) | 41,27 | 45.42 | Tiao Ping Kang tablets + LSM LSM | Every day, intake 12 tablets of Tiao Ping Kang and LSM or LSM. | 8 | Mercury, 3 |
| Zhengli Tang, 2014^42^ | China | Adults | SBP:120-139 DBP:80-89 | 66 (34,32) | 38,28 | 44.94 | Self-developed TCM prescription + LSM LSM | Every day, intake 400ml of self-developed TCM formula + LSM or LSM. | 26 | NA |
| Hua Yan, 2010^43^ | China | Adults | SBP:120-139 DBP:80-89 | 85 (40,41) | 65,20 | 49.04 | Tongxinluo LSM | Every day,  intake 8 capsules of Tongxinluo or placebo. | 12 | NA, 3 |
| Jiangyun Liu, 2019^44^ | China | Adults | SBP:120-139 DBP:80-89 | 104 (35,36,33) | 36,68 | 43.91 | Medication and edible intervention LSM No intervention | Every day, intake 250 mL of the dialectic medicine and food formula for 6 weeks* 2 courses + LSM or LSM or no intervention. | 12 | Mercury, 3 |
| Hongbo Li, 2011^45^ | China | Adults | SBP:120-139 DBP:80-89 | 123 (68,55) | 78,45 | 54.55 | Self-developed TCM prescription No intervention | Every day, intake TCM formula (Yiqijianpi decoction) or no intervention. |  | Mercury, 3 |
| Xiuwen Du, 2017^46^ | China | Adults | SBP:130-139 DBP:85-89 | 120 (60,60) | 74,46 | 41.5 | Self-developed TCM prescription + LSM LSM | Every day, intake TCM formula, based on TCM syndrome type + LSM or LSM. | 52 | NA |
| Gengzhong Xie, 2017^47^ | China | Adults | SBP:120-139 DBP:80-89 | 60 (30,30) | 31,29 | 45.6 | Self-developed TCM prescription + LSM LSM | Every day, intake TCM formula (Dachaihu decoction+Guizhi fuling wan) + LSM or LSM. | 4 | Mercury, 3 |
| Rui Zhang, 2014^48^ | China | Adults | SBP:120-139 DBP:80-89 | 228 (114,114) | 105,123 | 55.89 | Self-developed TCM prescription + LSM LSM | Every day, intake TCM formula (Qutan Huoluo decoction) + LSM or LSM. | 48 | Mercury, 3 |
| Haiping Sun, 2019^49^ | China | Adults | SBP:120-139 DBP:80-89 | 50 (25,25) | 28,22 | 58.6 | Self-developed TCM prescriptio No intervention | Every day, intake TCM formula (Tianxiangma decoction) or no intervention. | 6 | Electronic, NA |
| Lilin Zhao, 2014^50^ | China | Adults | SBP:120-139 DBP:80-89 | 230 (122,108) | 121,109 | 55.77 | Self-developed TCM prescription + LSM LSM | Every day, intake TCM formula (Tianma Shuangxi granules) + LSM or LSM. | 24 | NA |
| Jinxiu Wang, 2015^51^ | China | Adults | SBP:130-139 DBP:85-89 | 336 (224,112) | 146,190 | 55.77 | Self-developed TCM prescription + LSM LSM | Every day, intake TCM formula, based on TCM syndrome type + LSM or LSM. | 96 | Mercury, 3 |
| Chunying Huang, 2017^52^ | China | Adults | SBP:120-139 DBP:80-89 | 100 (50,50) | 57,43 | 45.5 | TCD bubble Usual care | Each day, use a self-developed TCM formula to foot soak for 30 min or usual care. | 4 | Mercury, 3 |
| Jiali Zeng, 2016^53^ | China | Adults | SBP:120-139 DBP:80-89 | 120 (60,60) | 76,44 | 55.5 | TCD bubble + LSM LSM | Each day, use a self-developed TCM formula to foot soak for 20-30 min or LSM. | 4 | NA |
| X Y Xu, 2007^54^ | China | Youth | SBP:120-139 DBP:80-89 | 49 (27,22) | NR | 19-23 | EMG biofeedback + relaxation techniques Relaxation techniques | Every three days, receive 25min EMG training + relaxation techniques or relaxation techniques. | 30 days | Mercury, NA |
| ShuZhen Wang, 2010^55^ | China | Postmenopausal women | SBP:120-139 DBP:80-89 | 22 (12,10) | 0,22 | 52.55 | EMG+ abdominal respiratory Abdominal breathing | Every three days, receive 25 min EMG + abdominal respiratory or simple abdominal breathing. | 30 days | Mercury, 3 |
| YuWang, 2022^56^ | China | Adults | SBP:120-139 DBP:80-89 | 60 (30,30) | 22,38 | 54.32 | TEAS + LSM LSM | Everyday, proform TEAS for 30min + LSM or LSM. | 12 | Electronic, 3 |
| Sue Xv, 2020^57^ | China | Adults | SBP:120-139 DBP:80-89 | 45 (15,15,15) | 29,16 | 34-62 | Moxibustion 3 times a week Moxibustion 2 times a week No intervention | Weekly, moxibustion at 5 acupuncture points 2 or 3 times, 40 min each or no intervention | 4 | Mercury, NA |
| Xin Zhao, 2020^58^ | China | Adults | SBP:120-139 DBP:80-89 | 70 (35,35) | 33,37 | 43.56 | Ear acupressure + LSM LSM | Every day, press at 8 acupuncture points for 24 min + LSM or LSM. | 4 | NA |
| Ting Li, 2020^59^ | China | Adults | SBP:120-139 DBP:80-89 | 88 (45,43) | 39,49 | 45.27 | Pressure acupoints LSM | Every day,  press at 4 acupoints (100 times each) +LSM or LSM. | 52 | NA |
| Xiaobin Chang, 2016^60^ | China | Adults | SBP:120-139 DBP:80-89 | 60 (30,30) | 32,28 | 20.58 | Pressure acupoints LSM | Every day,  press at 2 acupoints +LSM or LSM. | 6 | Mercury, 3 |
| Meixuan Sun, 2017^61^ | China | Freshman | SBP:120-139 DBP:80-89 | 97 (48,49) | NA | 19.38 | Ear acupressure Usual care | Every day, press at 3-5 acupoints 10-20 times, 4-5 times a day or usual care. | 6 | NA |
| Jiahui Yao, 2019^62^ | China | Adults | SBP:120-139 DBP:80-89 | 108 (53,55) | 54,54 | 46.35 | Pressure acupoints Usual care | Every day,  press at specific body acupoints 1 to 2 times (≥5 times/week) or usual care. | 24 | Electronic, 2 |
| Souvik Dutta, 2022^63^ | India | Adults | SBP:120-139 DBP:80-89 | 84 (42,42) | 30,62 | 46.25 | Verum LSM | Once daily for 1-2 days,proform homeopathic medicines + LSM  or LSM. | 12 | Electronic, 2 |
| The TOHP Research Group, 1992 c^14^ | America | Adults | DBP:80-89 | 564 (308,256) | 385,179 | 30-54 | Weight loss  Usual care | Everyday, reduce calorie intake + feed back adjustments or LSM. | 72 | Hawksleyrandom-zerosphygmomanometer, 3 |
| The TOHP Research Group, 1992 b^14^ | America | Adults | DBP:80-89 | 562 (242,320) | 398,164 | 30-54 | MBSR Usual care | Everyday, practice MBSR techniques 45 min or LSM. | 24 | Hawksleyrandom-zerosphygmomanometer, 3 |
| Imtiyaz Ali Mir, 2021^64^ | Malaysia | Young men | SBP:120-139 DBP:80-89 | 30 (15,15) | 30,0 | 21.05 | Music + dietary intervention Dietary intervention | 5 days/week musical intervention (30 minutes) + dietary intervention (DASH diet + sodium intake < 100 mmol/day)  or dietary intervention. | 4 | Electronic, 3 |
| Marquez-Celedonio, 2009^65^ | Mexico | Adults | SBP:120-139 DBP:80-89 | 81 (38,43) | NR | 43.2 | LSM Usual care | Low sodium, DASH diet daily + 3-5 aerobic exercises (45 min/week/time) + health education or usual recommendations. | 24 | NA |
| Zujian Huang, 2015^66^ | China | Adults | SBP:120-139 DBP:80-89 | 1120 (542,578) | 487,519 | 44.96 | LSM No intervention | Control salt intake control, reduce alcohol consumption or no alcohol consumption, reduce smoking or quit smoking, exercise consistently, weight control or no intervention. | 260 | NA |
| Yuhua He, 2005^67^ | China | Adults | SBP:120-139 DBP:80-89 | 120 (60,60) | NR | 30-50 | LSM No intervention | Health education, exercise intervention, nutrition intervention, intensive education, psychological interventions or no intervention. | 52 | NA |
| Xiaoyan Liu, 2014^68^ | China | Adults | SBP:120-139 DBP:80-89 | 240 (120,120) | 125,115 | 45.6 | LSM No intervention | Control salt intake control, reduce alcohol consumption or no alcohol consumption, reduce smoking or quit smoking, exercise consistently, weight control or no intervention. | 24 | Mercury, 2 |
| Yan Chen, 2008^69^ | China | Adults | SBP:120-139 DBP:80-89 | 253 (128,125) | 178,88 | 58.2 | LSM Usual care | Control salt intake control, reduce alcohol consumption or no alcohol consumption, reduce smoking or quit smoking, exercise consistently, weight control or usual care. | 52 | NA |
| Yang Li, 2016^70^ | China | Adults | SBP:120-139 DBP:80-89 | 422 (188,234) | 188,234 | 46.9 | LSM No intervention | Control salt intake control, reduce alcohol consumption or no alcohol consumption, reduce smoking or quit smoking, exercise consistently, weight control or no intervention. | 72 | NA |
| Zhifang Zheng, 2016^71^ | China | Adults | SBP:120-139 DBP:80-89 | 100 (50,50) | 56,44 | 63.15 | LSM No intervention | Provide guidance on diet, emotional and mental health, living and exercise or no intervention. | 52 | NA |
| Guangxiu Chen, 2011^72^ | China | Adults | SBP:120-139 DBP:80-89 | 120 (60,60) | 68,52 | 35-45 | LSM Usual care | Control salt intake control, reduce alcohol consumption or no alcohol consumption, reduce smoking or quit smoking, exercise consistently, weight control or usual care. | 52 | NA |
| Ye Yang, 2013^73^ | China | Adults | SBP:120-139 DBP:80-89 | 400 (200,200) | 200,200 | 40-75 | LSM No intervention | Control salt intake control, reduce alcohol consumption or no alcohol consumption, reduce smoking or quit smoking, exercise consistently, weight control or no intervention. | 104 | NA |
| Xia Peng, 2018^74^ | China | Adults | SBP:120-139 DBP:80-89 | 80 (40,40) | 48,32 | 49 | TCM constitution intervention Usual care | Based on the constitution of tcm, diet, mood and lifestyle guidance based on the constitution of TCM or usual care. | 52 | Mercury, 3 |
| Shaojuan Qiu, 2014^75^ | China | Adults | SBP:120-139 DBP:80-89 | 172 (86,86) | 88,84 | 51.1 | TCM constitution intervention Usual care | Based on the constitution of tcm, diet, mood and lifestyle guidance based on the constitution of TCM or usual care. | 104 | NA |
| Xiaowen Wang, 2022^76^ | China | Adults | SBP:120-139 DBP:80-89 | 196 (97,99) | NR | 40-70 | TCM constitution intervention LSM | Based on the constitution of tcm, diet, mood and lifestyle guidance based on the constitution of TCM or LSM. | 12 | NA |
| Rundi Song, 2018^77^ | China | Adults | SBP:120-139 DBP:80-89 | 169 (85,84) | 97,72 | 51.9 | TCM constitution intervention Usual care | Based on the constitution of tcm, diet, mood and lifestyle guidance based on the constitution of TCM or usual care. | 52 | NA |
| Adolfo Rubinstein, 2016^78^ | Argentina | Low-resource people in Latin America | SBP:120-139 DBP:80-89 | 637 (316,321) | 295,342 | 43.4 | Web-based LSM Usual care | Receive monthly counselling calls and weekly text messages to adjust diet and PA or usual care. | 52 | Electronic, 3 |
| Patricia A Hageman, 2014^79^ | America | Rural women | SBP:120-139 DBP:80-89 | 289 (116,115,58) | 0,289 | 56.4 | Print-based LSM Web-based LSM Usual care | Health advice + goal setting + customized communications (web-based or printed mail) or receive usual care. | 52 | Electronic, 2 |
| Xia Peng, 2021^80^ | China | Adults | SBP:130-139 DBP:85-89 | 120 (60,60) | 65,55 | 45.92 | Web-based LSM LSM | Based on the constitution of tcm, diet, mood and lifestyle guidance based on the constitution of TCM or LSM based on TCM. | 52 | NA |
| Habib Sadeghi-Gandomani, 2021^81^ | Iran | Adults | SBP:120-139 DBP:80-89 | 81 (41,40) | 39,42 | 39.96 | Web-based LSM No intervention | Receive monthly counselling calls and weekly text messages to adjust diet and PA or no intervention. | 8 | NA, 3 |
| Jatuporn Phoemsapthawee, 2021^82^ | Thailand | Obesity youth | SBP:120-139 DBP:80-89 | 20 (10,10) | 20,0 | 20 | AE+RE No intervention | 4 times a week, perform 30 minutes of AE(50-60% progression to 60-70% HR max), 20 minutes of RE, and 5 minutes of stretch exercise or no intervention. | 12 | Electronic, 3 |
| X.S. Zhao, 2014^83^ | China | Adults | SBP:120-139 DBP:80-89 | 1112 (278,278, 278,278) | 543,569 | 43 | LSM + drug LSM Compound reserpine tablets Uaual care | Everyday, proform LSM or intake a Compound reserpine tablets or usual care. | 52 | NA |
| Zhi Zhang, 2014^84^ | China | Adults | SBP:120-139 DBP:80-89 | 90 (30,30,30) | 44,46 | 58.1 | Irbesartan +LSM LSM No intervention | Every day, intake irbesartan (150 m g)+LSM or LSM or no intervention. | 52 | NA, 3 |
| Jun Gu, 2021^85^ | China | Adults | SBP:120-139 DBP:80-89 | 118 (58,60) | 51,67 | 68.67 | Ear acupressure + Baduanjin Usual care | Every day, press at 8 acupuncture points for 24-32 min and perform Badaanjin for 15 min or usual care. | 52 | NA |
| Mengmeng Hu, 2016^86^ | China | Adults | SBP:120-139 DBP:80-89 | 100 (50,50) | 56,44 | 68.67 | Brisk walking + acupressure Usual care | Brisk walking 5 times/week (30-40 min each) to reach 60% of the maximum HR + every night acupressure (8-10 hours) 10 times * 5 courses of treatment. | 52 | NA |
| Stevo Julius, 2006^87^ | America | Adults | SBP:130-139 DBP:85-89 | 772 (391,381) | 460,312 | 48.5 | Candesartan Placebo | Every day, intake candesartan tablets (16mg) or placebo. | 104 | Electronic, 3 |
| Dazhi Ding, 2012^88^ | China | With dizziness and headache | SBP:120-139 DBP:80-89 | 86 (43,43) | 44,42 | 43.55 | Candesartan + LSM LSM | Every day, intake candesartan tablets (2-8mg)+LSM or LSM. | 4 | NA |
| Nan Zheng, 2013^89^ | China | Adults | SBP:130-139 DBP:85-89 | 104 (26,26,52) | 53,51 | 62.57 | Temisartan + LSM Indapamide + LSM LSM | Every day,  intake temisartan (40 mg)  or indapamide (1.5 mg)  or placebo. | 52 | Electronic, 3 |
| Weiji Zhong, 2013^90^ | China | Adults | SBP:120-139 DBP:80-89 | 238 (124,114) | 126,112 | 40.8 | Irbesartan Placebo | NR | 78 | NA |
| Yaping Lv, 2013^91^ | China | Adults | SBP:130-139 DBP:85-89 | 165 (85,80) | 80,85 | 58.79 | Temisartan Placebo | Every day,  intake temisartan (40mg)  or placebo. | 52 | NA |
| Jie Peng, 2015^92^ | China | Abdominal obesity | SBP:130-139 DBP:85-89 | 664 (221,213,230) | 208,456 | 64.31 | Telmisartan Indapamide Placebo | Everyday intake telmisartan (40 mg)  or every day or every other day intake indapamide (1.5mg)  or placebo. | 156 | Electronic, 3 |
| Yingjun Chen, 2016^93^ | China | Adults | SBP:120-139 DBP:80-89 | 1015 (509,506) | 599,416 | 38.46 | Temisartan + LSM LSM | Every day,  intake temisartan (40mg) + LSM or placebo. | 312 | NA |
| Stephan Lu¨ders, 2008^94^ | German | Age 50 - 85 years | SBP:120-139 DBP:80-89 | 1008 (505,503) | 488,520 | 62.2 | Ramipril Placebo | Every day, intake ramipril from 1.5mg to 5mg gradually or placebo. | 156 | Electronic, 3 |
| Jason T Davis, 2013^95^ | America | Adults | SBP:120-139 DBP:80-89 | 50 (25,25) | 30,20 | 33.7 | Nebivolol Placebo | Everyday, intake nebivolol (5 mg)  or placebo. | 8 | Monitor, 3 |
| Ramprasad Kandavar, 2015^96^ | America | Adults | SBP:120-139 DBP:80-89 | 34 (33,32) | 17,15 16,16 | 49 | Nebivolol  Placebo | Everyday, intake nebivolol (5 mg)  or placebo. | 8 | Electronic, 3 |
| Dazhi Ding, 2013^97^ | China | With dizziness and headache | SBP:120-139 DBP:80-89 | 68(34,34) | 38,68 | 42.95 | Amlodipine besylate LSM | Everyday, intake amlodipine besylate (2.5-5 mg) and LSM or LSM. | 4 | NA |
| Fuchs SC, 2016^98^ | Brazil | Adults | SBP:120-139 DBP:80-89 | 730 (372,358) | 363,367 | 50 | Chlorthalidone+Amiloride Placebo | Everyday, intake chlorthalidone (12.5 mg) + plus amiloride (2.5 mg)  or placebo. | 78 | Electronic, 6 |
| Wenyan Jiang, 2011^99^ | China | With hyperlipidemia | SBP:120-139 DBP:80-89 | 80 (40,40) | 49,31 | 51.85 | Atorvastatin Placebo | Everyday, intake atorvastatin calcium (20 mg) or placebo. | 12 | Mercury, 3 |
| Hua Yan, 2010^100^ | China | Young men with hyperlipidemia | SBP:120-139 DBP:80-89 | 63 (33,30) | 63,0 | 39.5 | Atorvastatin Placebo | Everyday, intake atorvastatin calcium (20 mg) or placebo. | 12 | NA, 3 |
| Wenyan Jiang, 2012^101^ | China | With hyperlipidemia | SBP:120-139 DBP:80-89 | 120 (30,30,30) | 69,51 | 59.8 | Regular dose atorvastatin Low dose atorvastatin Placebo | Everyday, intake atorvastatin calcium (20 mg or 10mg)  or placebo. | 12 | Mercury, 3 |
| Magdalena Madero, 2015 b^24^ | Mexico | Overweight or obese Adults with a history of high fructose consumption | SBP:120-139 DBP:80-89 | 72 (36,36) | 44,38 | 46 | Allopurinol Placebo | Everyday, intake allopurinol (300 mg/d)  or placebo. | 4 | Mercury, 3 |

AE , aerobic exercise ; RE , resistance exercise ; HIIT , high-intensity interval training ; CMT , continuous moderate-intensity training ; IRT , isometric resistance exercise ; LSM , lifestyle modification ; DASH , dietary approaches to stop hypertension ; GSE , grapefruit seed extract ; GTE , green tea extract ; CL , codonopsis lanceolata ; TCM , traditional Chinese medicine ; EMG , electromyographic ; TEAS , transcutaneous electroacupuncture stimulation ; MBSR , mindfulness-based stress reduction ; HR , heart rate ; NR , not reported.

References：

1. 肖刘, 锋张, 亚星, 叶晓琴, 李彬. 有氧运动加弹力带对正常高值血压人群血压及心血管病危险因素的干预. *中国疗养医学* 2014;**23**:869-871. doi: 10.13517/j.cnki.ccm.2014.10.003

2. Prasertsri P, Singsanan S, Chonanant C, Boonla O, Trongtosak P. Effects of arm swing exercise training on cardiac autonomic modulation, cardiovascular risk factors, and electrolytes in persons aged 60-80 years with prehypertension: A randomized controlled trial. *J Exerc Sci Fit* 2019;**17**:47-54. doi: 10.1016/j.jesf.2018.11.002

3. 杨琴. 广场舞对正常高值血压人群健身的影响效果研究 硕士. 赣南师范大学, 2020.

4. John AT, Chowdhury M, Islam MR*, et al.* Effectiveness of High-Intensity Interval Training and Continuous Moderate-Intensity Training on Blood Pressure in Physically Inactive Pre-Hypertensive Young Adults. *J Cardiovasc Dev Dis* 2022;**9**. doi: 10.3390/jcdd9080246

5. Skutnik BC, Smith JR, Johnson AM, Kurti SP, Harms CA. The Effect of Low Volume Interval Training on Resting Blood Pressure in Pre-hypertensive Subjects: A Preliminary Study. *Phys Sportsmed* 2016;**44**:177-183. doi: 10.1080/00913847.2016.1159501

6. Beck DT, Martin JS, Casey DP, Braith RW. Exercise training reduces peripheral arterial stiffness and myocardial oxygen demand in young prehypertensive subjects. *Am J Hypertens* 2013;**26**:1093-1102. doi: 10.1093/ajh/hpt080

7. 刘忠浩. 血流限制抗阻训练对血压正常高值人群血压和心率变异性的影响 硕士. 辽宁师范大学, 2021.

8. O'Driscoll JM, Edwards JJ, Coleman DA*, et al.* One year of isometric exercise training for blood pressure management in men: a prospective randomized controlled study. *J Hypertens* 2022;**40**:2406-2412. doi: 10.1097/hjh.0000000000003269

9. Thiyagarajan R, Pal P, Pal GK*, et al.* Additional benefit of yoga to standard lifestyle modification on blood pressure in prehypertensive subjects: a randomized controlled study. *Hypertens Res* 2015;**38**:48-55. doi: 10.1038/hr.2014.126

10. 郑翠红, 陈楚, 李华萍. DASH饮食联合生活方式改变对社区正常高值血压人群的作用. *海峡预防医学杂志* 2013;**19**:71-72. doi:

11. 王家威, 樊琼玲, 张雪莲*, et al.* 终止高血压膳食疗法(DASH)对乌鲁木齐南山牧区牧民高血压前期的干预研究. *新疆医科大学学报* 2020;**43**:962-966+975. doi:

12. Abolhosseini H, Setayesh M, Namayandeh Sm*, et al.* The effect of improving dietary habits derived from Persian Medicine on blood pressure in adults with pre-hypertension: A randomized controlled clinical trial. *Journal of Complementary Medicine Research* 2021;**12**:91. doi: 10.5455/jcmr.2021.12.02.13

13. The Hypertension Prevention Trial: three-year effects of dietary changes on blood pressure. Hypertension Prevention Trial Research Group. *Arch Intern Med* 1990;**150**:153-162. doi:

14. The effects of nonpharmacologic interventions on blood pressure of persons with high normal levels. Results of the Trials of Hypertension Prevention, Phase I. *Jama* 1992;**267**:1213-1220. doi: 10.1001/jama.1992.03480090061028

15. Allaert FA. Effect of NaCl + Chitosan 3% vs. NaCl on high blood pressure parameters of healthy volunteers with prehypertension. *Minerva Cardioangiol* 2017;**65**:563-576. doi: 10.23736/s0026-4725.17.04451-6

16. Rodríguez-Ramírez M, Rodríguez-Morán M, Reyes-Romero MA, Guerrero-Romero F. Effect of oral magnesium supplementation on the transcription of TRPM6, TRPM7, and SLC41A1 in individuals newly diagnosed of pre-hypertension. A randomized, double-blind, placebo-controlled trial. *Magnes Res* 2017;**30**:80-87. doi: 10.1684/mrh.2017.0426

17. Cadée JA, Chang CY, Chen CW*, et al.* Bovine casein hydrolysate (c12 Peptide) reduces blood pressure in prehypertensive subjects. *Am J Hypertens* 2007;**20**:1-5. doi: 10.1016/j.amjhyper.2006.06.005

18. Ried K, Frank OR, Stocks NP. Dark chocolate or tomato extract for prehypertension: a randomised controlled trial. *BMC Complement Altern Med* 2009;**9**:22. doi: 10.1186/1472-6882-9-22

19. Figueroa A, Sanchez-Gonzalez MA, Perkins-Veazie PM, Arjmandi BH. Effects of watermelon supplementation on aortic blood pressure and wave reflection in individuals with prehypertension: a pilot study. *Am J Hypertens* 2011;**24**:40-44. doi: 10.1038/ajh.2010.142

20. Wichitsranoi J, Weerapreeyakul N, Boonsiri P*, et al.* Antihypertensive and antioxidant effects of dietary black sesame meal in pre-hypertensive humans. *Nutr J* 2011;**10**:82. doi: 10.1186/1475-2891-10-82

21. Biswas OS, Gonzalez VR, Schwarz ER. Effects of an oral nitric oxide supplement on functional capacity and blood pressure in adults with prehypertension. *J Cardiovasc Pharmacol Ther* 2015;**20**:52-58. doi: 10.1177/1074248414539563

22. Lockyer S, Rowland I, Spencer JPE, Yaqoob P, Stonehouse W. Impact of phenolic-rich olive leaf extract on blood pressure, plasma lipids and inflammatory markers: a randomised controlled trial. *Eur J Nutr* 2017;**56**:1421-1432. doi: 10.1007/s00394-016-1188-y

23. Jeong HS, Hong SJ, Cho JY*, et al.* Effects of Rubus occidentalis extract on blood pressure in patients with prehypertension: Randomized, double-blinded, placebo-controlled clinical trial. *Nutrition* 2016;**32**:461-467. doi: 10.1016/j.nut.2015.10.014

24. Madero M, Rodríguez Castellanos FE, Jalal D*, et al.* A pilot study on the impact of a low fructose diet and allopurinol on clinic blood pressure among overweight and prehypertensive subjects: a randomized placebo controlled trial. *J Am Soc Hypertens* 2015;**9**:837-844. doi: 10.1016/j.jash.2015.07.008

25. Park E, Edirisinghe I, Choy YY, Waterhouse A, Burton-Freeman B. Effects of grape seed extract beverage on blood pressure and metabolic indices in individuals with pre-hypertension: a randomised, double-blinded, two-arm, parallel, placebo-controlled trial. *Br J Nutr* 2016;**115**:226-238. doi: 10.1017/s0007114515004328

26. Sun Q, Wang B, Li Y*, et al.* Taurine Supplementation Lowers Blood Pressure and Improves Vascular Function in Prehypertension: Randomized, Double-Blind, Placebo-Controlled Study. *Hypertension* 2016;**67**:541-549. doi: 10.1161/hypertensionaha.115.06624

27. Pelliccia F, Pasceri V, Marazzi G*, et al.* Randomised, double-blind, placebo-controlled, assessment of the efficacy and safety of dietary supplements in prehypertension. *J Hum Hypertens* 2017;**31**:647-653. doi: 10.1038/jhh.2017.35

28. Nogueira LP, Nogueira Neto JF, Klein MR, Sanjuliani AF. Short-term Effects of Green Tea on Blood Pressure, Endothelial Function, and Metabolic Profile in Obese Prehypertensive Women: A Crossover Randomized Clinical Trial. *J Am Coll Nutr* 2017;**36**:108-115. doi: 10.1080/07315724.2016.1194236

29. Park HJ, Yadav D, Jeong DJ*, et al.* Short-Term Consumption of Cuban Policosanol Lowers Aortic and Peripheral Blood Pressure and Ameliorates Serum Lipid Parameters in Healthy Korean Participants: Randomized, Double-Blinded, and Placebo-Controlled Study. *Int J Environ Res Public Health* 2019;**16**. doi: 10.3390/ijerph16050809

30. Odai T, Terauchi M, Kato K, Hirose A, Miyasaka N. Effects of Grape Seed Proanthocyanidin Extract on Vascular Endothelial Function in Participants with Prehypertension: A Randomized, Double-Blind, Placebo-Controlled Study. *Nutrients* 2019;**11**. doi: 10.3390/nu11122844

31. Alatas H, Sja'bani M, Mustofa M*, et al.* The effects of soursop supplementation on blood pressure, serum uric acid, and kidney function in a prehypertensive population in accordance with the 2017 ACC/AHA guideline. *J Hum Hypertens* 2020;**34**:223-232. doi: 10.1038/s41371-019-0235-6

32. van der Avoort CMT, Ten Haaf DSM, Bongers C*, et al.* Increasing Nitrate-Rich Vegetable Intake Lowers Ambulatory Blood Pressure in (pre)Hypertensive Middle-Aged and Older Adults: A 12-Wk Randomized Controlled Trial. *J Nutr* 2021;**151**:2667-2679. doi: 10.1093/jn/nxab157

33. Vors C, Rancourt-Bouchard M, Couillard C*, et al.* Sex May Modulate the Effects of Combined Polyphenol Extract and L-citrulline Supplementation on Ambulatory Blood Pressure in Adults with Prehypertension: A Randomized Controlled Trial. *Nutrients* 2021;**13**. doi: 10.3390/nu13020399

34. 牛凤琳. 西瓜提取物对高血压前期人群血压的影响研究 硕士. 苏州大学, 2012.

35. Cha TW, Kim M, Kim M, Chae JS, Lee JH. Blood pressure-lowering effect of Korean red ginseng associated with decreased circulating Lp-PLA2 activity and lysophosphatidylcholines and increased dihydrobiopterin level in prehypertensive subjects. *Hypertens Res* 2016;**39**:449-456. doi: 10.1038/hr.2016.7

36. Shin YK, Hsieh YS, Han AY, Lee KW, Seol GH. Beneficial effects of Codonopsis lanceolata extract on systolic blood pressure levels in prehypertensive adults: A double-blind, randomized controlled trial. *Phytother Res* 2020;**34**:340-348. doi: 10.1002/ptr.6520

37. Shen L, Gwak SR, Joo JC*, et al.* Effectiveness and Safety of Hwangchil-Unripe Bokbunja Extract Mixture on Blood Pressure: A Randomized Double-Blind Placebo-Controlled Clinical Trial. *J Med Food* 2021;**24**:258-266. doi: 10.1089/jmf.2020.4820

38. Siddiqui MA, Itrat M, Mobeen A, Khan MI. Efficacy of Khār-i-khasak (Tribulus terrestris Linn.) in prehypertension: a randomized, double-blind, placebo-controlled trial. *J Complement Integr Med* 2021;**18**:783-789. doi: 10.1515/jcim-2020-0322

39. Beyranvand H, Tasharrofi N, Nooryazdan A, Sheyni M, Nazari A. The Effect of Zingiber Officinale Rhizome (ginger) Herbal Formulation on Blood

Pressure and Lipid Profiles in Patients with Pre-Hypertension. *International Journal of Early Childhood Special Education (INT-JECSE)* 2022;**14**. doi: 10.9756/INT-JECSE/V14I2.317

40. 武双平, 李彦霞, 肖红, 房会文. 复方珍珠降压胶囊治疗高血压前期疗效观察. *上海中医药杂志* 2014;**48**:44-46. doi: 10.16305/j.1007-1334.2014.09.014

41. 吴辉, 丁有钦. 调平康联合生活方式干预对高血压前期患者的短期疗效观察. *湖北中医杂志* 2011;**33**:6-8. doi:

42. 汤峥丽, 张晓一, 张晓天. 自拟平肝益肾健脾方联合生活方式干预对高血压前期人群的短期疗效观察. *中西医结合心脑血管病杂志* 2014;**12**:1180-1182. doi:

43. 严华. 通心络胶囊对临界高血压患者血压和血脂的影响. *医学研究杂志* 2010;**39**:101-103. doi:

44. 刘绛云, 周经钲, 王永生*, et al.* 药食干预痰湿体质高血压前期疗效评价. *实用中医内科杂志* 2019;**33**:66-69. doi: 10.13729/j.issn.1671-7813.z20190327

45. 李洪波, 岳桂华, 罗莎, 梁焕英, 丁家明. 益气健脾方对高血压前期人群血压影响因素的干预作用. *新中医* 2011;**43**:25-26. doi: 10.13457/j.cnki.jncm.2011.02.028

46. 杜修文, 郝正燕, 代君*, et al.* 中医辨证论治联合生活方式干预对正常高值血压人群的血压水平分析. *中西医结合心血管病电子杂志* 2017;**5**:82-83. doi: 10.16282/j.cnki.cn11-9336/r.2017.04.055

47. 谢更钟. 大柴胡汤合桂枝茯苓丸在社区高血压前期中的疗效观察 硕士. 广州中医药大学, 2017.

48. 张蕊. 祛痰活络方对228例正常高值血压（痰瘀互结证）的影响 硕士. 北京中医药大学, 2014.

49. 孙海平. 香天麻汤干预阴虚阳亢型正常高值血压人群的疗效观察 硕士. 新疆医科大学, 2019.

50. 赵李琳. 天麻双膝颗粒对高血压前期肝肾阴虚型人群的干预研究 硕士. 北京中医药大学, 2014.

51. 王金秀. 中医辨证治疗正常高值血压的临床研究 硕士. 中国中医科学院, 2015.

52. 黄春英. 中药泡足干预正常高值血压50例临床观察. *中国民族民间医药* 2017;**26**:91-93. doi:

53. 曾家丽. 中药泡足在阳虚质正常高值血压人群中的应用. *齐鲁护理杂志* 2016;**22**:66-67. doi:

54. Xu XY, Gao J, Ling D, Wang TH. Biofeedback treatment of prehypertension: analyses of efficacy, heart rate variability and EEG approximate entropy. *J Hum Hypertens* 2007;**21**:973-975. doi: 10.1038/sj.jhh.1002237

55. Wang SZ, Li S, Xu XY*, et al.* Effect of slow abdominal breathing combined with biofeedback on blood pressure and heart rate variability in prehypertension. *J Altern Complement Med* 2010;**16**:1039-1045. doi: 10.1089/acm.2009.0577

56. Wang Y, Yang JW, Liu JH*, et al.* Home-based transcutaneous electrical acupoint stimulation for high-normal blood pressure: A randomized controlled trial. *J Clin Hypertens (Greenwich)* 2022;**24**:984-992. doi: 10.1111/jch.14496

57. 徐素娥, 姜九. 艾灸疗法治疗高血压前期患者的临床观察. *实用临床医药杂志* 2020;**24**:115-118. doi:

58. 赵鑫, 刘振岳. 耳穴贴压配合生活方式干预治疗痰瘀互结型高血压前期的临床研究. *中国处方药* 2018;**16**:102-103. doi:

59. 李婷, 嵇冰. 四关穴按压对成年高血压前期人群血压的影响. *浙江中医杂志* 2020;**55**:256-257. doi: 10.13633/j.cnki.zjtcm.2020.04.011

60. 常晓宾, 郭珺, 杨迪*, et al.* 穴位按摩对大学生高血压前期血压的影响. *现代医药卫生* 2016;**32**:2320-2321+2324. doi:

61. 孙美漩. 耳穴压豆对大学生正常高值血压的疗效观察 硕士. 山东中医药大学, 2021.

62. 姚家慧. 降压按摩操干预正常高值血压人群的临床研究 硕士. 上海中医药大学, 2019.

63. Dutta S, Ganguly S, Mukherjee SK*, et al.* Efficacy of individualized homeopathic medicines in intervening with the progression of pre-hypertension to hypertension: A double-blind, randomized, placebo-controlled trial. *Explore (NY)* 2022;**18**:279-286. doi: 10.1016/j.explore.2021.05.007

64. Mir IA, Chowdhury M, Islam RM*, et al.* Relaxing music reduces blood pressure and heart rate among pre-hypertensive young adults: A randomized control trial. *J Clin Hypertens (Greenwich)* 2021;**23**:317-322. doi: 10.1111/jch.14126

65. Márquez-Celedonio FG, Téxon-Fernández O, Chávez-Negrete A*, et al.* [Clinical effect of lifestyle modification on cardiovascular risk in prehypertensives: PREHIPER I study]. *Rev Esp Cardiol* 2009;**62**:86-90. doi:

66. 黄祖坚. 改善生活方式对高血压前期人群血压的影响. *中国当代医药* 2015;**22**:144-146. doi:

67. 何玉华. 高血压前期不良行为的护理干预. *当代护士(学术版)* 2005:35-37. doi:

68. 刘小颜, 谭鄂, 李虹. 高血压前期人群社区护理干预调查研究. *华西医学* 2014;**29**:1332-1335. doi:

69. 陈燕, 黄辉斌, 赵茜. 门诊评价性健康教育干预对正常高值血压人群的影响. *中医药导报* 2008:73-74. doi:

70. 李洋, 胡洁, 郭海健*, et al.* 社区高血压前期生活方式群组干预研究. *中华疾病控制杂志* 2016;**20**:971-974. doi: 10.16462/j.cnki.zhjbkz.2016.10.001

71. 郑至方. 社区护理对正常高值血压老年人群的干预效果. *中国城乡企业卫生* 2016;**31**:161-162. doi: 10.16286/j.1003-5052.2016.11.068

72. 陈广秀, 万青, 张清群, 曾露. 社区护理干预对高血压前期人群的影响. *中华现代护理杂志* 2011:2645-2647. doi:

73. 杨烨, 程磊, 黄小明, 陈秀萍, 陈刚. 社区综合干预对高血压前期人群血压控制及血管功能的影响. *中外医学研究* 2013;**11**:16-19. doi: 10.14033/j.cnki.cfmr.2013.13.026

74. 彭霞. 基于中医体质的健康教育对正常高值血压人群干预效果研究; 2018.

75. 邱少娟, 李华, 肖灿, 黄凤珍, 黄玉梅. 中医护理对社区高血压前期人群血压干预的效果; 2014.

76. 王晓筱, 吴琰, 冯剑, 陈国华. 中医体质辨识对正常高值血压者踝臂脉搏波传导速度和踝臂指数的影响. *中国现代药物应用* 2022;**16**:153-156. doi: 10.14164/j.cnki.cn11-5581/r.2022.12.050

77. 宋润娣, 陈峻鹏, 单莉, 贵琳. 中医体质辨识及干预在高血压前期治未病健康管理中的效果研究. *四川中医* 2018;**36**:195-197. doi:

78. Rubinstein A, Miranda JJ, Beratarrechea A*, et al.* Effectiveness of an mHealth intervention to improve the cardiometabolic profile of people with prehypertension in low-resource urban settings in Latin America: a randomised controlled trial. *Lancet Diabetes Endocrinol* 2016;**4**:52-63. doi: 10.1016/s2213-8587(15)00381-2

79. Hageman PA, Pullen CH, Hertzog M, Boeckner LS. Effectiveness of tailored lifestyle interventions, using web-based and print-mail, for reducing blood pressure among rural women with prehypertension: main results of the Wellness for Women: DASHing towards Health clinical trial. *Int J Behav Nutr Phys Act* 2014;**11**:148. doi: 10.1186/s12966-014-0148-2

80. 彭霞, 彭锦绣, 刘倩*, et al.* 正常高值血压人群的互联网+中医健康管理. *护理学杂志* 2021;**36**:43-45. doi:

81. Sadeghi-Gandomani H, Habibi Z, Eghbali-Babadi M, Khosravi A. Impact of Telenursing on Blood Pressure and Body Mass Index of People with Prehypertension: A Randomized Controlled Clinical Trial. *Iran J Nurs Midwifery Res* 2021;**26**:544-549. doi: 10.4103/ijnmr.IJNMR_113_19

82. Phoemsapthawee J, Sriton B. Combined exercise training improves blood pressure at rest and during exercise in young obese prehypertensive men. *J Sports Med Phys Fitness* 2021;**61**:468-479. doi: 10.23736/s0022-4707.20.11222-2

83. Zhao XS, Wang R, Bin LR, Wa SQ. Intervention for prehypertension and its cardiovascular risk factors in Inner Mongolia. *Genet Mol Res* 2014;**13**:4867-4882. doi: 10.4238/2014.July.4.1

84. 张志. 综合方式干预对高血压前期患者周围动脉硬化的影响. *中外医学研究* 2014;**12**:157-159. doi: 10.14033/j.cnki.cfmr.2014.15.047

85. 顾君, 邝海东, 张晓琼, 王朝昕, 吕奕鹏. 耳穴贴压结合八段锦练习对上海宜川社区血压正常高值居民的影响. *上海医药* 2021;**42**:46-50. doi:

86. 胡蒙蒙, 尹莲花, 许艺惠, 黄守清. 快步走配合穴位贴敷对高血压前期疗效的影响. *湖南中医杂志* 2016;**32**:15-17. doi: 10.16808/j.cnki.issn1003-7705.2016.07.006

87. Julius S, Nesbitt SD, Egan BM*, et al.* Feasibility of treating prehypertension with an angiotensin-receptor blocker. *N Engl J Med* 2006;**354**:1685-1697. doi: 10.1056/NEJMoa060838

88. 丁大植, 尤昕, 方恩男, 朴美娜, 关立克. 坎地沙坦酯片对正常高值血压患者的疗效观察. *吉林医学* 2012;**33**:5393-5394. doi:

89. 郑楠. 正常高值血压伴心血管危险因素者的干预性研究 硕士. 河北医科大学, 2013.

90. 钟伟济. 降压药物干预下高血压前期的血尿酸、C-反应蛋白变化. *中国医药指南* 2013;**11**:55-56. doi: 10.15912/j.cnki.gocm.2013.16.258

91. 吕雅萍, 袁琛, 马晓丽*, et al.* 替米沙坦对高血压前期伴危险因素患者脉搏波传导速度和踝臂指数的影响. *中国医药导刊* 2013;**15**:466-467. doi:

92. Peng J, Zhao Y, Zhang H*, et al.* Prevention of metabolic disorders with telmisartan and indapamide in a Chinese population with high-normal blood pressure. *Hypertens Res* 2015;**38**:123-131. doi: 10.1038/hr.2014.148

93. 陈应军, 王倩, 周新平, 王益品. 替米沙坦干预血压正常高值的效果评估. *医药论坛杂志* 2016;**37**:124-125. doi:

94. Lüders S, Schrader J, Berger J*, et al.* The PHARAO study: prevention of hypertension with the angiotensin-converting enzyme inhibitor ramipril in patients with high-normal blood pressure: a prospective, randomized, controlled prevention trial of the German Hypertension League. *J Hypertens* 2008;**26**:1487-1496. doi: 10.1097/HJH.0b013e3282ff8864

95. Davis JT, Pasha DN, Khandrika S*, et al.* Central hemodynamics in prehypertension: effect of the β-adrenergic antagonist nebivolol. *J Clin Hypertens (Greenwich)* 2013;**15**:69-74. doi: 10.1111/jch.12031

96. Kandavar R, Fernandez C, Sander GE*, et al.* Digital plethysmography and arginine metabolism in prehypertension: effect of nebivolol therapy. *J Clin Hypertens (Greenwich)* 2015;**17**:14-19. doi: 10.1111/jch.12451

97. 丁大植, 元奎昌, 尤欣*, et al.* 苯磺酸氨氯地平片对正常高值血压的疗效观察. *延边大学医学学报* 2013;**36**:204-206. doi: 10.16068/j.1000-1824.2013.03.016

98. Fuchs SC, Poli-de-Figueiredo CE, Figueiredo Neto JA*, et al.* Effectiveness of Chlorthalidone Plus Amiloride for the Prevention of Hypertension: The PREVER-Prevention Randomized Clinical Trial. *J Am Heart Assoc* 2016;**5**. doi: 10.1161/jaha.116.004248

99. 江文艳, 宋巧凤, 王希柱, 郭永辉. 阿托伐他汀对高血压前期合并高脂血症患者血压及妊娠相关血浆蛋白A的影响. *中国全科医学* 2011;**14**:3741-3742. doi:

100. 严华, 符春晖. 阿托伐他汀对高血脂合并高血压前期血压的影响. *中国医学创新* 2010;**7**:8-9. doi:

101. 江文艳, 宋巧凤. 不同剂量阿托伐他汀对高血压前期合并高脂血症患者血脂及血压的影响. *中国药房* 2012;**23**:1519-1520. doi:

Supplementary Table 4. Model DIC values (SBP, DBP)

| Outcome | Model | Dbar | pD | DIC | I^2^ |
| --- | --- | --- | --- | --- | --- |
| SBP | Fixed effects model | 218.3048 | 212.387 | 430.6918 | 0% |
|  | Random effects model | 2843.7115 | 136.7192 | 2980.4307 | 92% |
| DBP | Fixed effects model | 215.6124 | 204.9267 | 420.5391 | 0.30% |
|  | Random effects model | 1661.5607 | 133.4679 | 1795.0285 | 87% |

Supplementary Table 5. Risk of bias assessment for inclusion in studies

| First author,  year | Sequence generation of the allocation | Allocation concealment | Blinding of participants, personnel | Blinding of outcome assessors | Incomplete outcome data | Selective outcome reporting | Other sources of bias | Risk of bias for study |
| --- | --- | --- | --- | --- | --- | --- | --- | --- |
| Xuan Xiao, 2014 | Unclear | Low | Unclear | Low | Low | Unclear | Low | Low |
| Piyapong Prasertsri, 2018 | Low | Low | Unclear | Low | Low | Unclear | Low | Low |
| Qin Yang, 2020 | Unclear | Low | Unclear | Low | Low | Unclear | Low | Low |
| Anil T John, 2022 | Unclear | Low | Unclear | Low | Low | Unclear | Low | Low |
| Benjamin C, 2016 | Unclear | Low | Unclear | Low | Low | Unclear | Low | Low |
| Darren T Beck, 2013 | Low | Low | Low | Low | Low | Unclear | Low | Low |
| Zhonghao Liu, 2021 | Low | Low | Unclear | Low | Low | Unclear | Low | Low |
| Jamie M. O’Driscoll, 2022 | Unclear | Low | Unclear | Low | Low | Unclear | Low | Low |
| Thiyagarajan,  2015 | Low | Low | High | Low | Low | Low | Unclear | Low |
| Cuihong Zheng, 2013 | Unclear | Low | Unclear | Low | Low | Unclear | Low | Low |
| Jiawei Wang, 2020 | Low | Low | Unclear | Low | Low | Unclear | Low | Low |
| Hossein abolhosseini, 2021 | Low | Low | Low | Low | Low | Low | Low | Low |
| HPT Research Group, 1990 | Unclear | Low | Unclear | Low | High | Unclear | Low | High |
| The TOHP Research Group,1992a | Low | Low | Unclear | Low | Low | Low | Low | Low |
| François-André Allaert, 2017 | Unclear | Low | Low | Low | Low | Unclear | Low | Low |
| The TOHP Research Group, 1992 d | Low | Low | Low | Low | Low | Low | Low | Low |
| Mariana Rodríguez-Ramírez, 2017 | Low | Low | Low | Low | Low | Unclear | Low | Low |
| The TOHP Research Group, 1992 e | Low | Low | Low | Low | Low | Low | Low | Low |
| Jenny A Cadée, 2007 | Unclear | Low | Low | Low | Unclear | Unclear | Low | Moderate |
| Karin Ried, 2009 | Low | Low | High | Low | Low | Low | Low | Low |
| Arturo Figueroa, 2011 | Unclear | Low | Low | Low | Low | Unclear | Low | Low |
| Jatuporn Wichitsranoi, 2011 | Unclear | Low | Low | Low | Low | Unclear | Unclear | Low |
| Biswas OS, 2015 | Unclear | Low | Low | Low | Low | Low | Low | Low |
| Stacey Lockyer, 2015 | Low | Low | Low | Low | Low | Low | Low | Low |
| Han Saem Jeong, 2016 | Low | Low | Low | Low | Low | Low | Unclear | Low |
| Magdalena Madero, 2015 a | Low | Low | Unclear | Low | High | Low | High | High |
| Eunyoung Park, 2016 | Low | Low | Low | Low | Low | Low | Low | Low |
| Qianqian Sun, 2016 | Low | Low | Low | Low | Low | Low | Low | Low |
| F Pelliccia, 2017 | Unclear | Low | Low | Low | Low | Low | Low | Low |
| Lívia de P. Nogueira MD, 2017 | Unclear | Low | Low | Low | Unclear | Low | Low | Low |
| Hye-Jeong Park, 2019 | Low | Low | Low | Low | High | Unclear | Low | Low |
| Tamami Odai, 2019 | Unclear | Low | Low | Low | Low | Unclear | Unclear | Low |
| Haidar Alatas, 2020 | Low | Low | Unclear | Low | Low | Low | Low | Low |
| Cindy MT van der Avoort, 2021 | Low | Low | Low | Low | Low | Low | Low | Low |
| Cécile Vors, 2021 | Low | Low | Low | Low | Unclear | Low | Low | Low |
| Fenlin Niu, 2012 | Low | Low | Unclear | Low | Low | Unclear | Low | Low |
| Tae Woong Cha, 2016 | Low | Low | Low | Low | Unclear | Low | Low | Low |
| You Kyoung Shin, 2019 | Unclear | Low | Low | Low | Unclear | Unclear | Low | Moderate |
| Lei Shen, 2021 | Low | Low | Low | Low | Low | Low | Low | Low |
| Mansoor Ahmad Siddiqui, 2021 | Low | Low | Low | Low | Unclear | Low | Low | Low |
| Hossein Beyranvand, 2022 | Unclear | Low | Low | Low | Low | Unclear | Low | Low |
| Shuangping Wu, 2014 | Low | Low | Unclear | Low | Low | Unclear | Low | Low |
| Hui Wu, 2011 | Unclear | Low | Unclear | Low | Low | Low | Low | Low |
| Zhengli Tang, 2014 | Unclear | Low | Unclear | Low | Low | Unclear | Low | Low |
| Hua Yan, 2010 | Unclear | Low | Unclear | Low | Low | Unclear | Low | Low |
| Jiangyun Liu, 2019 | Low | Low | Unclear | Low | High | Unclear | Low | High |
| Hongbo Li, 2011 | Unclear | Low | Unclear | Low | Low | Unclear | Low | Low |
| Xiuwen Xie, 2017 | Unclear | Low | Unclear | Low | Low | Unclear | Low | Low |
| Gengzhong Xie, 2017 | Low | Low | Unclear | Low | Low | Unclear | Low | Low |
| Rui Zhang, 2014 | Low | Low | Unclear | Low | Low | Unclear | Low | Low |
| Haiping Sun, 2019 | Low | Low | Unclear | Low | Low | Unclear | Low | Low |
| Lilin Zhao, 2014 | Low | Low | Unclear | Low | Low | Unclear | Low | Low |
| Jinxiu Wang, 2015 | Unclear | Low | Unclear | Low | Low | Unclear | Low | Low |
| Chunying Huang, 2017 | Unclear | Low | Unclear | Low | Low | Unclear | Low | Low |
| Jiali Zeng, 2016 | Unclear | Low | Unclear | Low | Low | Unclear | Low | Low |
| X Y Xu, 2007 | Low | Low | Unclear | Low | Unclear | Unclear | Low | Low |
| ShuZhen Wang, 2010 | Unclear | Low | Unclear | Low | Unclear | Unclear | Low | Moderate |
| YuWang, 2022 | Low | Low | Unclear | Low | Unclear | Low | Low | Low |
| Sue Xv, 2020 | Unclear | Low | Unclear | Low | Low | Unclear | Low | Low |
| Xin Zhao, 2020 | Low | Low | Unclear | Low | Low | Unclear | Low | Low |
| Ting Li, 2020 | Low | Low | Unclear | Low | Low | Unclear | Low | Low |
| Xiaobin Chang, 2016 | Unclear | Low | Unclear | Low | Low | Unclear | Low | Low |
| Meixuna Sun, 2017 | Low | Low | Unclear | Low | High | Unclear | High | High |
| Jiahui Yao, 2019 | Low | Low | Unclear | Low | High | Unclear | Low | High |
| Souvik Dutta, 2022 | Low | Low | Low | Low | Low | Low | Low | Low |
| The TOHP Research Group, 1992 c | Low | Low | Unclear | Low | Low | Low | Low | Low |
| The TOHP Research Group, 1992 b | Low | Low | Unclear | Low | Low | Low | Low | Low |
| Imtiyaz Ali Mir, 2021 | Unclear | Low | High | Low | Low | Unclear | Unclear | Low |
| Marquez-Celedonio, 2009 | Low | Low | High | Low | Low | Low | Low | Low |
| Zujian Huang, 2015 | Unclear | Low | High | Low | High | Unclear | Low | High |
| Yuhua He, 2005 | Unclear | Low | High | Low | Low | Unclear | Unclear | Low |
| Xiaoyan Liu, 2014 | Unclear | Low | High | Low | Low | Unclear | Low | Low |
| Yan Chen, 2008 | Unclear | Low | High | Low | Low | Unclear | Low | Low |
| Yang Li, 2016 | Unclear | Low | High | Low | Low | Unclear | Low | Low |
| Zhifang Zheng, 2016 | Unclear | Low | High | Low | Low | Unclear | Low | Low |
| Guangxiu Chen, 2011 | Low | Low | High | Low | Low | Unclear | Low | Low |
| Ye Yang, 2013 | Low | Low | High | Low | Low | Unclear | Low | Low |
| Xia Peng, 2018 | Unclear | Low | Unclear | Low | Low | Unclear | Low | Low |
| Shaojuan Wiu, 2014 | Unclear | Low | High | Low | Low | Unclear | Low | Low |
| Xiaowen Wang, 2022 | Unclear | Low | High | Low | Low | Unclear | Low | Low |
| Rundi Song, 2018 | Unclear | Low | High | Low | High | Unclear | High | High |
| Adolfo Rubinstein, 2016 | Low | Low | Unclear | Low | High | Unclear | Low | High |
| Patricia A Hageman, 2014 | Low | Low | Low | Low | Low | Low | Low | Low |
| Xia Peng, 2021 | Low | Low | Unclear | Low | Low | Low | Unclear | Low |
| Habib Sadeghi-Gandomani, 2021 | Low | Low | Unclear | Low | Low | Low | Low | Low |
| Jatuporn Phoemsapthawee, 2021 | Low | Low | Unclear | Low | Low | Unclear | Low | Low |
| X.S. Zhao, 2014 | Unclear | Low | Unclear | Low | High | Unclear | Low | High |
| Zhi Zhang, 2014 | Unclear | Low | Unclear | Low | Low | Unclear | Low | Low |
| Jun Gu, 2021 | Low | Low | Unclear | Low | High | Unclear | Low | High |
| Mengmeng Hu, 2016 | Unclear | Low | Unclear | Low | Low | Unclear | Low | Low |
| Stevo Julius, 2006 | Low | Low | Low | Low | Low | Low | Low | Low |
| Dazhi Ding, 2012 | Unclear | Low | Unclear | Low | Low | Unclear | Low | Low |
| Nan Zheng, 2013 | Unclear | Low | Unclear | Low | Low | Unclear | Low | Low |
| Weiji Zhong, 2013 | Unclear | Low | Unclear | Low | High | Unclear | High | High |
| Yaping Lv, 2013 | High | Low | Unclear | Low | Low | Unclear | Low | High |
| Jie Peng, 2015 | Low | Low | Low | Low | Low | Unclear | Low | Low |
| Yingjun Chen, 2016 | Unclear | Low | Unclear | Low | Low | Unclear | Low | Low |
| Stephan Lu¨ders, 2008 | Low | Low | Low | Low | Low | Low | Low | Low |
| Jason T Davis, 2013 | Unclear | Low | Low | Low | Low | Unclear | High | High |
| Ramprasad Kandavar, 2015 | Unclear | Low | Low | Low | Unclear | Unclear | Low | Moderate |
| Dazhi Ding, 2013 | Unclear | Low | Unclear | Low | High | Unclear | Low | High |
| Fuchs SC, 2016 | Low | Low | Low | Low | High | Low | Low | High |
| Wenyan Jiang, 2011 | Low | Low | Unclear | Low | Low | Unclear | Low | Low |
| Hua Yan, 2010 | Unclear | Low | Unclear | Low | Low | Unclear | Low | Low |
| Wenyan Jiang, 2012 | Low | Low | Unclear | Low | Low | Unclear | Low | Low |
| Magdalena Madero, 2015 b | Low | Low | Unclear | Low | High | Low | High | High |

Supplementary Table 6. Egger test for BP

|  | Std_Eff | Coef. | Std. Err. | t | P>\|t\| | [95% Conf. Interval] | |
| --- | --- | --- | --- | --- | --- | --- | --- |
| SBP | slope | 340.0644 | 11.19794 | 30.37 | 0 | 317.8913 | 362.2374 |
|  | bias | 13.14134 | 21.18579 | 0.62 | 0.536 | -28.80865 | 55.09132 |
| DBP | slope | 234.9842 | 12.63759 | 18.59 | 0 | 209.9583 | 260.0101 |
|  | bias | 7.797961 | 40.70017 | 0.19 | 0.848 | -72.79947 | 88.39539 |

Supplementary Table 7. Network meta-analysis of the antihypertensive effect of SBP and DBP

| Control | **-8.03** | **-7.46** | **-6** | -4.6 | **-5.81** | -3.9 | -2.18 | 0.46 | **-2.06** | **-5.24** | **-5.87** | -2.98 | -2.69 | -1.58 |
| --- | --- | --- | --- | --- | --- | --- | --- | --- | --- | --- | --- | --- | --- | --- |
|  | **(-12.25, -3.69)** | **(-14.89, -0.13)** | **(-11.24, -0.59)** | (-12.63, 3.52) | **(-10.8, -0.81)** | (-12.01, 4.29) | (-6.91, 2.46) | (-3.41, 4.37) | **(-4.08, -0.06)** | **(-7.42, -3.09)** | **(-11.7, -0.08)** | (-8.93, 3) | (-5.86, 0.51) | (-9.68, 6.49) |
| **-10.65** | AE | 0.6 | 2 | 3.43 | 2.23 | 4.13 | 5.83 | 8.47 | 5.98 | 2.79 | 2.16 | 5.05 | 5.34 | 6.46 |
| **(-17.12, -4.32)** |  | (-6.01, 7.18) | (-4.74, 8.87) | (-5.71, 12.58) | (-3.38, 7.8) | (-5.22, 13.38) | (-0.59, 12.22) | (2.71, 14.32) | (1.16, 10.6) | (-2.1, 7.57) | (-5.13, 9.4) | (-2.33, 12.27) | (-0.04, 10.64) | (-2.58, 15.35) |
| **-12.46** | -1.75 | HIIT | 1.43 | 2.91 | 1.65 | 3.58 | 5.26 | 7.92 | 5.38 | 2.2 | 1.56 | 4.47 | 4.72 | 5.86 |
| **(-22.42, -2.39)** | (-10.66, 6.89) |  | (-7.53, 10.72) | (-8.09, 13.87) | (-5.89, 9.16) | (-7.34, 14.49) | (-3.52, 13.99) | (-0.42, 16.22) | (-2.26, 13.05) | (-5.47, 9.95) | (-7.78, 10.99) | (-5, 13.74) | (-3.33, 12.77) | (-4.99, 16.7) |
| -6.54 | 4.11 | 5.9 | RE | 1.46 | 0.24 | 2.19 | 3.87 | 6.46 | 3.97 | 0.77 | 0.13 | 3.07 | 3.35 | 4.42 |
| (-13.87, 0.71) | (-5.48, 13.93) | (-6.44, 18.44) |  | (-8.29, 11.12) | (-7.13, 7.42) | (-7.7, 11.83) | (-3.37, 10.82) | (-0.2, 13.05) | (-1.8, 9.62) | (-5.01, 6.46) | (-7.79, 8.03) | (-4.97, 11) | (-3, 9.54) | (-5.39, 14.08) |
| -7.73 | 2.86 | 4.66 | -1.21 | Yoga | -1.22 | 0.72 | 2.38 | 5.05 | 2.54 | -0.64 | -1.33 | 1.61 | 1.89 | 2.96 |
| (-20.29, 4.44) | (-11.12, 16.66) | (-11.19, 20.42) | (-15.92, 13.24) |  | (-10.73, 8.24) | (-10.75, 12.22) | (-6.95, 11.57) | (-3.96, 14.03) | (-5.87, 10.76) | (-8.82, 7.46) | (-10.99, 8.38) | (-8.51, 11.65) | (-6.74, 10.48) | (-8.43, 14.29) |
| **-9.21** | 1.46 | 3.23 | -2.69 | -1.48 | DASH | 1.93 | 3.61 | 6.24 | 3.76 | 0.56 | -0.08 | 2.87 | 3.14 | 4.22 |
| **(-16.47, -1.67)** | (-6.72, 9.63) | (-6.83, 13.52) | (-13.13, 7.81) | (-15.73, 13) |  | (-7.56, 11.5) | (-3.26, 10.44) | (-0.04, 12.68) | (-1.68, 9.11) | (-4.88, 6.12) | (-7.65, 7.58) | (-5, 10.52) | (-2.79, 9.03) | (-5.32, 13.78) |
| -7.13 | 3.54 | 5.27 | -0.55 | 0.67 | 1.99 | TPM dietary habit | 1.71 | 4.36 | 1.83 | -1.38 | -2.04 | 0.88 | 1.12 | 2.29 |
| (-19.32, 5.19) | (-10.2, 17.43) | (-10.6, 21.25) | (-14.73, 13.62) | (-16.68, 18.28) | (-12.27, 16.51) |  | (-7.75, 11.09) | (-4.77, 13.3) | (-6.64, 10.13) | (-9.86, 7.03) | (-11.9, 8.12) | (-9.34, 10.94) | (-7.57, 9.98) | (-9.21, 13.49) |
| -3.86 | 6.79 | 8.56 | 2.67 | 3.86 | 5.34 | 3.21 | Salt restriction | 2.64 | 0.11 | -3.06 | -3.72 | -0.85 | -0.48 | 0.62 |
| (-10.95, 3.15) | (-2.74, 16.22) | (-3.76, 20.86) | (-7.5, 12.83) | (-10.37, 18.37) | (-4.94, 15.38) | (-10.89, 17.28) |  | (-3.42, 8.85) | (-4.89, 5.25) | (-8.2, 2.14) | (-11.15, 3.93) | (-8.16, 6.85) | (-6.14, 5.25) | (-8.77, 9.89) |
| -3.27 | 7.38 | 9.19 | 3.29 | 4.53 | 5.94 | 3.9 | 0.63 | Micronutrients | -2.52 | **-5.7** | -6.37 | -3.44 | -3.15 | -2.04 |
| (-8.99, 2.61) | (-1.31, 16.04) | (-2.46, 20.84) | (-6.01, 12.58) | (-9.11, 18.29) | (-3.43, 15.4) | (-9.72, 17.41) | (-8.54, 9.84) |  | (-6.76, 1.7) | **(-10.17, -1.24)** | (-13.25, 0.58) | (-10.57, 3.72) | (-8.21, 1.88) | (-11.13, 6.87) |
| **-4.27** | 6.37 | 8.18 | 2.27 | 3.47 | 4.93 | 2.83 | -0.4 | -1.03 | Food extract | **-3.18** | -3.81 | -0.91 | -0.64 | 0.45 |
| **(-7.24, -1.31)** | (-0.69, 13.43) | (-2.26, 18.61) | (-5.59, 10.06) | (-9.18, 16.32) | (-3.12, 12.91) | (-9.74, 15.42) | (-8.06, 7.31) | (-7.3, 5.18) |  | **(-6.16, -0.22)** | (-9.99, 2.4) | (-7.22, 5.36) | (-4.35, 3.25) | (-7.86, 8.83) |
| **-8.51** | 2.15 | 3.92 | -2 | -0.79 | 0.71 | -1.39 | -4.62 | -5.26 | -4.25 | Medicinal herbs Taking | -0.64 | 2.29 | 2.56 | 3.67 |
| **(-11.71, -5.33)** | (-5.16, 9.39) | (-6.59, 14.5) | (-9.94, 6.01) | (-13.28, 11.96) | (-7.48, 8.68) | (-14.13, 11.25) | (-12.47, 3.07) | (-11.95, 1.29) | (-8.61, 0.1) |  | (-6.66, 5.45) | (-4.1, 8.67) | (-1.1, 6.26) | (-4.76, 11.95) |
| **-13.44** | -2.74 | -0.96 | -6.86 | -5.59 | -4.19 | -6.28 | -9.57 | -10.19 | -9.15 | -4.89 | TCD bubble | 2.91 | 3.18 | 4.31 |
| **(-22, -4.64)** | (-13.57, 8.06) | (-14.17, 12.23) | (-18.06, 4.42) | (-20.61, 9.43) | (-15.41, 7.18) | (-21.26, 8.62) | (-20.78, 1.8) | (-20.65, 0.38) | (-18.23, 0.02) | (-13.93, 4.25) |  | (-5.35, 11.16) | (-3.25, 9.7) | (-5.67, 14.21) |
| -5.6 | 5.09 | 6.84 | 0.96 | 2.17 | 3.66 | 1.6 | -1.75 | -2.34 | -1.3 | 2.94 | 7.85 | EMG | 0.28 | 1.41 |
| (-14.46, 3.32) | (-5.75, 15.87) | (-6.59, 20.11) | (-10.41, 12.4) | (-13, 17.52) | (-8.07, 15.04) | (-13.63, 16.66) | (-12.91, 9.55) | (-12.85, 8.12) | (-10.55, 8.01) | (-6.44, 12.4) | (-4.66, 20.16) |  | (-6.47, 7.1) | (-8.68, 11.35) |
| **-7.3** | 3.36 | 5.17 | -0.74 | 0.49 | 1.92 | -0.16 | -3.41 | -4.01 | -3.04 | 1.23 | 6.13 | -1.73 | Acupoint therapy | 1.1 |
| **(-12.1, -2.57)** | (-4.7, 11.41) | (-6.06, 16.21) | (-9.53, 8.03) | (-12.52, 13.5) | (-6.97, 10.63) | (-13.49, 12.98) | (-11.91, 5.1) | (-11.64, 3.42) | (-8.69, 2.54) | (-4.25, 6.68) | (-3.77, 15.85) | (-11.74, 8.47) |  | (-7.71, 9.75) |
| -2.72 | 7.89 | 9.66 | 3.8 | 5.01 | 6.45 | 4.33 | 1.05 | 0.49 | 1.53 | 5.75 | 10.57 | 2.78 | 4.53 | Homeopathy |
| (-14.95, 9.27) | (-5.83, 21.55) | (-6.38, 25.3) | (-10.58, 18.02) | (-12.51, 22.37) | (-7.82, 20.61) | (-13.11, 21.66) | (-13.07, 15.05) | (-13.21, 14.03) | (-11.17, 13.85) | (-6.92, 18.23) | (-4.23, 25.66) | (-12.54, 17.76) | (-8.59, 17.55) |  |
| -2.82 | 7.77 | 9.55 | 3.59 | 4.82 | 6.33 | 4.2 | 0.97 | 0.4 | 1.4 | 5.64 | 10.57 | 2.71 | 4.42 | -0.07 |
| (-14.79, 9.11) | (-5.82, 21.31) | (-6.09, 25.2) | (-10.25, 17.67) | (-12.21, 22.21) | (-7.7, 20.26) | (-13.18, 21.33) | (-12.92, 14.76) | (-12.76, 13.53) | (-10.85, 13.6) | (-6.69, 17.85) | (-4.35, 25.24) | (-12.28, 17.51) | (-8.55, 17.24) | (-17.47, 16.77) |
| -4.23 | 6.42 | 8.24 | 2.33 | 3.57 | 4.98 | 2.89 | -0.33 | -0.95 | 0.01 | 4.29 | 9.23 | 1.38 | 3.04 | -1.49 |
| (-12.9, 4.44) | (-4.39, 17.24) | (-5.08, 21.43) | (-9.03, 13.78) | (-11.59, 18.6) | (-6.57, 16.49) | (-12.17, 17.8) | (-11.59, 10.83) | (-11.34, 9.49) | (-9.07, 9.32) | (-4.93, 13.53) | (-3.24, 21.44) | (-11.24, 13.77) | (-6.87, 13.2) | (-16.25, 13.65) |
| **-5.77** | 4.87 | 6.68 | 0.75 | 1.99 | 3.45 | 1.39 | -1.9 | -2.5 | -1.49 | 2.75 | 7.64 | -0.19 | 1.5 | -3 |
| **(-8.44, -3.12)** | (-2.08, 11.84) | (-3.69, 17.06) | (-7.07, 8.44) | (-9.91, 14.29) | (-4.57, 11.24) | (-11.3, 13.9) | (-9.48, 5.6) | (-9.01, 3.81) | (-5.54, 2.47) | (-0.56, 6) | (-1.15, 16.34) | (-9.37, 9.01) | (-3.36, 6.38) | (-15.34, 9.47) |
| -4.27 | 6.39 | 8.16 | 2.3 | 3.46 | 4.92 | 2.86 | -0.4 | -1 | 0.02 | 4.21 | 9.15 | 1.31 | 3.03 | -1.55 |
| (-10.44, 1.8) | (-2.54, 15.24) | (-3.72, 19.82) | (-7.35, 11.75) | (-10.17, 17.59) | (-4.64, 14.49) | (-10.94, 16.53) | (-9.77, 9.04) | (-9.45, 7.43) | (-6.73, 6.75) | (-2.66, 11.15) | (-1.62, 19.65) | (-9.37, 11.98) | (-4.64, 10.71) | (-15.14, 12.28) |
| -4.1 | 6.52 | 8.34 | 2.41 | 3.59 | 5.1 | 3.03 | -0.21 | -0.89 | 0.18 | 4.42 | 9.26 | 1.43 | 3.16 | -1.36 |
| (-9.67, 1.35) | (-1.94, 14.93) | (-3.13, 19.8) | (-6.83, 11.48) | (-9.66, 17.16) | (-4.13, 14.15) | (-10.54, 16.6) | (-9.24, 8.64) | (-8.92, 7.13) | (-6.19, 6.34) | (-1.94, 10.67) | (-1.08, 19.56) | (-8.81, 11.9) | (-4.06, 10.51) | (-14.81, 12.04) |
| -8.23 | 2.43 | 4.16 | -1.71 | -0.46 | 1.03 | -1.09 | -4.38 | -5 | -3.93 | 0.28 | 5.13 | -2.58 | -0.93 | -5.46 |
| (-16.72, 0.38) | (-6.97, 11.91) | (-8.09, 16.88) | (-12.89, 9.61) | (-15.19, 14.79) | (-10, 11.96) | (-16.15, 13.89) | (-15.53, 7) | (-15.18, 5.51) | (-12.84, 5.12) | (-8.72, 9.45) | (-6.93, 17.43) | (-14.93, 9.69) | (-10.69, 8.97) | (-20.46, 9.55) |
| **-11.07** | -0.4 | 1.33 | -4.54 | -3.22 | -1.86 | -3.9 | -7.16 | -7.76 | -6.78 | -2.53 | 2.35 | -5.47 | -3.76 | -8.3 |
| **(-18.5, -3.49)** | (-10.19, 9.5) | (-11.09, 13.8) | (-15.16, 5.86) | (-17.35, 10.96) | (-12.34, 8.63) | (-18.17, 10.38) | (-17.48, 3.25) | (-17.32, 1.62) | (-14.77, 1.28) | (-10.41, 5.47) | (-9.11, 13.82) | (-16.78, 6.14) | (-12.44, 4.96) | (-22.27, 6.07) |
| -10.48 | 0.22 | 2.04 | -3.89 | -2.75 | -1.2 | -3.31 | -6.56 | -7.18 | -6.19 | -1.91 | 2.93 | -4.87 | -3.18 | -7.61 |
| (-22.33, 1.6) | (-13.31, 13.88) | (-13.66, 17.76) | (-17.93, 10.22) | (-19.84, 14.76) | (-15.25, 12.85) | (-20.44, 13.79) | (-20.3, 7.47) | (-20.48, 6.13) | (-18.34, 6.24) | (-14.35, 10.54) | (-11.81, 17.74) | (-19.43, 10.26) | (-15.89, 9.91) | (-24.81, 9.54) |
| -1.42 | 9.14 | 11.03 | 5.08 | 6.36 | 7.81 | 5.72 | 2.44 | 1.85 | 2.86 | 7.06 | 11.97 | 4.14 | 5.89 | 1.29 |
| (-13.45, 10.76) | (-4.38, 22.93) | (-4.69, 26.65) | (-8.87, 19.26) | (-10.91, 24) | (-6.37, 22.03) | (-11.47, 22.94) | (-11.38, 16.71) | (-11.42, 15.11) | (-9.56, 15.42) | (-5.33, 19.75) | (-2.82, 26.69) | (-10.66, 19.15) | (-6.92, 18.64) | (-15.75, 18.62) |
| **-12.5** | -1.86 | -0.1 | -5.96 | -4.79 | -3.33 | -5.38 | **-8.64** | **-9.23** | **-8.23** | -4 | 0.87 | -6.93 | -5.21 | -9.85 |
| **(-17.59, -7.54)** | (-10.09, 6.29) | (-11.24, 11.12) | (-14.92, 2.72) | (-17.77, 8.52) | (-12.32, 5.61) | (-18.76, 7.78) | **(-17.21, -0.02)** | **(-16.9, -1.72)** | **(-14.06, -2.52)** | (-9.71, 1.63) | (-9.03, 10.55) | (-17.25, 3.09) | (-11.93, 1.49) | (-22.94, 3.58) |
| -2.56 | 8.15 | 9.9 | 4.01 | 5.23 | 6.69 | 4.59 | 1.35 | 0.69 | 1.72 | 5.96 | 10.85 | 3.07 | 4.76 | 0.15 |
| (-11.32, 6.31) | (-2.7, 19.1) | (-3.4, 23.27) | (-7.5, 15.36) | (-9.97, 20.68) | (-4.94, 18.15) | (-10.44, 19.63) | (-9.84, 12.65) | (-10.01, 11.31) | (-7.49, 11.14) | (-3.25, 15.45) | (-1.51, 23.29) | (-9.4, 15.65) | (-5.27, 14.78) | (-14.81, 15.38) |
| **-23.67** | -12.95 | -11.22 | **-17.09** | -15.91 | -14.39 | -16.55 | **-19.81** | **-20.44** | **-19.41** | **-15.2** | -10.23 | **-18.02** | **-16.37** | **-20.95** |
| **(-36.34, -11)** | (-27.32, 1.16) | (-27.55, 4.7) | **(-31.77, -2.46)** | (-33.57, 1.6) | (-29.04, 0.39) | (-34.21, 1.18) | **(-34.38, -5.36)** | **(-34.53, -6.36)** | **(-32.48, -6.33)** | **(-28.06, -2.3)** | (-25.69, 4.72) | **(-33.54, -2.58)** | **(-29.84, -2.97)** | **(-38.34, -3.16)** |
| **-7.92** | 2.79 | 4.57 | -1.37 | -0.18 | 1.28 | -0.74 | -4.01 | -4.62 | -3.62 | 0.61 | 5.52 | -2.3 | -0.6 | -5.12 |
| **(-14.44, -1.24)** | (-6.41, 11.92) | (-7.48, 16.64) | (-10.99, 8.39) | (-13.75, 14.03) | (-8.53, 11.11) | (-14.85, 13.24) | (-13.72, 5.53) | (-13.43, 4.16) | (-10.75, 3.58) | (-6.46, 7.75) | (-5.28, 16.28) | (-13.24, 8.68) | (-8.56, 7.45) | (-18.88, 8.8) |
| -5.32 | 5.34 | 7.08 | 1.23 | 2.41 | 3.92 | 1.87 | -1.48 | -2.04 | -1.04 | 3.2 | 8.07 | 0.24 | 1.95 | -2.6 |
| (-13.06, 2.41) | (-4.81, 15.43) | (-5.75, 20) | (-9.47, 11.93) | (-12.11, 17.2) | (-6.8, 14.56) | (-12.74, 16.3) | (-12.01, 9.08) | (-11.75, 7.64) | (-9.38, 7.25) | (-5.18, 11.56) | (-3.45, 19.71) | (-11.41, 11.93) | (-7.1, 11.3) | (-16.95, 11.83) |
| -5.95 | 4.7 | 6.51 | 0.57 | 1.75 | 3.28 | 1.14 | -2.08 | -2.73 | -1.67 | 2.56 | 7.43 | -0.37 | 1.36 | -3.13 |
| (-19.53, 7.46) | (-10.29, 19.72) | (-10.54, 23.53) | (-14.97, 15.87) | (-16.45, 20.11) | (-12.32, 18.63) | (-17.3, 19.5) | (-17.41, 13.3) | (-17.47, 11.91) | (-15.61, 12.16) | (-11.36, 16.33) | (-8.71, 23.52) | (-16.62, 15.68) | (-12.99, 15.64) | (-21.74, 15.03) |

| Continued supplementary Table 7 | | | | | | | | | | | | | | |
| --- | --- | --- | --- | --- | --- | --- | --- | --- | --- | --- | --- | --- | --- | --- |
| -2.2 | -0.71 | **-3.56** | -1.54 | -0.99 | **-11.02** | **-10.08** | 0.02 | -0.47 | **-6.42** | -2.42 | **-12.44** | -3.95 | -2.85 | -5.03 |
| (-9.9, 5.64) | (-6.49, 5.07) | **(-5.33, -1.79)** | (-5.56, 2.46) | (-4.92, 2.91) | **(-16.73, -5.21)** | **(-14.93, -5.28)** | (-8.28, 8.35) | (-8.58, 7.51) | **(-9.69, -3.26)** | (-8.09, 3.44) | **(-20.81, -4.04)** | (-8.24, 0.38) | (-8.27, 2.53) | (-13.31, 3.32) |
| 5.8 | 7.33 | 4.46 | 6.48 | 7 | -3.01 | -2.04 | 8.06 | 7.59 | 1.58 | 5.6 | -4.41 | 4.08 | 5.16 | 3.01 |
| (-3.06, 14.83) | (0.16, 14.49) | (-0.17, 9.09) | (0.63, 12.33) | (1.22, 12.88) | (-9.24, 3.22) | (-8.53, 4.36) | (-1.28, 17.28) | (-1.75, 16.58) | (-3.83, 7.02) | (-1.53, 12.81) | (-13.68, 4.93) | (-1.99, 10.13) | (-1.66, 12.02) | (-6.47, 12.46) |
| 5.24 | 6.74 | 3.87 | 5.91 | 6.44 | -3.64 | -2.63 | 7.51 | 7.01 | 1.04 | 5.02 | -4.93 | 3.49 | 4.53 | 2.47 |
| (-5.52, 15.94) | (-2.5, 16.14) | (-3.66, 11.53) | (-2.41, 14.28) | (-1.91, 14.78) | (-12.29, 5.38) | (-11.43, 6.18) | (-3.62, 18.46) | (-4.16, 17.68) | (-6.98, 9) | (-4.2, 14.37) | (-16.21, 6.23) | (-5.03, 12.06) | (-4.41, 13.75) | (-8.67, 13.6) |
| 3.82 | 5.31 | 2.45 | 4.47 | 5.05 | -4.99 | -4.06 | 6.06 | 5.55 | -0.37 | 3.59 | -6.44 | 2.06 | 3.17 | 0.97 |
| (-5.62, 13.25) | (-2.55, 13.21) | (-3.19, 8.04) | (-2.21, 11.07) | (-1.67, 11.69) | (-12.87, 2.86) | (-11.21, 3.03) | (-3.86, 16.03) | (-4.29, 15.17) | (-6.77, 5.71) | (-4.3, 11.47) | (-16.35, 3.56) | (-4.87, 8.95) | (-4.53, 10.67) | (-8.9, 10.91) |
| 2.32 | 3.87 | 1.02 | 3.04 | 3.6 | -6.45 | -5.47 | 4.61 | 4.06 | -1.83 | 2.19 | -7.89 | 0.59 | 1.71 | -0.43 |
| (-8.89, 13.57) | (-6.2, 13.67) | (-7.02, 8.91) | (-5.96, 12.05) | (-5.37, 12.58) | (-16.31, 3.41) | (-14.88, 3.74) | (-6.83, 15.88) | (-7.24, 15.37) | (-10.53, 6.64) | (-7.69, 12.09) | (-19.06, 3.48) | (-8.36, 9.68) | (-8.09, 11.57) | (-12.02, 11.14) |
| 3.57 | 5.09 | 2.25 | 4.25 | 4.82 | -5.25 | -4.24 | 5.85 | 5.34 | -0.63 | 3.37 | -6.67 | 1.85 | 2.96 | 0.78 |
| (-5.5, 12.81) | (-2.49, 12.64) | (-3.01, 7.54) | (-2.18, 10.69) | (-1.48, 11.13) | (-12.54, 2.2) | (-11.12, 2.68) | (-3.79, 15.32) | (-4.25, 14.79) | (-6.59, 5.33) | (-4.17, 11.06) | (-16.35, 3.09) | (-4.68, 8.48) | (-4.36, 10.4) | (-8.94, 10.45) |
| 1.67 | 3.15 | 0.32 | 2.32 | 2.9 | -7.17 | -6.19 | 3.87 | 3.43 | -2.57 | 1.43 | -8.56 | -0.08 | 0.99 | -1.15 |
| (-9.55, 12.91) | (-6.86, 13.11) | (-8.08, 8.64) | (-6.78, 11.34) | (-6.27, 11.79) | (-17.2, 2.83) | (-15.8, 3.37) | (-7.73, 15.57) | (-8.16, 14.89) | (-11.35, 6.2) | (-8.55, 11.43) | (-20.32, 2.85) | (-9.23, 9.08) | (-8.75, 10.87) | (-12.84, 10.69) |
| -0.04 | 1.49 | -1.38 | 0.64 | 1.2 | **-8.83** | **-7.87** | 2.23 | 1.73 | -4.26 | -0.23 | **-10.27** | -1.73 | -0.69 | -2.83 |
| (-8.96, 9.09) | (-5.94, 9.01) | (-6.34, 3.67) | (-5.6, 6.94) | (-4.96, 7.33) | **(-16.34, -1.33)** | **(-14.67, -1.11)** | (-7.29, 11.75) | (-7.6, 11.08) | (-9.89, 1.6) | (-7.65, 7.33) | **(-19.85, -0.61)** | (-8.09, 4.63) | (-7.76, 6.62) | (-12.28, 6.86) |
| -2.69 | -1.18 | -4.01 | -2.03 | -1.44 | **-11.49** | **-10.52** | -0.39 | -0.94 | **-6.87** | -2.88 | **-12.92** | -4.43 | -3.33 | -5.51 |
| (-11.33, 6.08) | (-8.18, 5.83) | (-8.36, 0.23) | (-7.62, 3.59) | (-7.08, 4.06) | **(-18.34, -4.49)** | **(-16.76, -4.34)** | (-9.74, 8.56) | (-10, 7.95) | **(-12.02, -1.89)** | (-9.74, 4.05) | **(-22.16, -3.6)** | (-10.24, 1.32) | (-9.92, 3.37) | (-14.68, 3.77) |
| -0.15 | 1.35 | -1.49 | 0.52 | 1.07 | **-8.97** | **-8.02** | 2.1 | 1.58 | **-4.35** | -0.36 | **-10.4** | -1.9 | -0.77 | -2.95 |
| (-8.12, 7.83) | (-4.79, 7.53) | (-4.16, 1.21) | (-3.92, 4.98) | (-3.34, 5.51) | **(-15.02, -2.79)** | **(-13.23, -2.81)** | (-6.45, 10.53) | (-6.79, 9.83) | **(-8.18, -0.59)** | (-6.41, 5.86) | **(-19, -1.75)** | (-6.61, 2.85) | (-6.54, 5) | (-11.53, 5.68) |
| 3.04 | 4.56 | 1.68 | 3.71 | 4.28 | -5.75 | -4.8 | 5.31 | 4.77 | -1.19 | 2.8 | -7.2 | 1.3 | 2.4 | 0.2 |
| (-4.89, 11.07) | (-1.54, 10.66) | (-0.43, 3.86) | (-0.94, 8.25) | (-0.18, 8.71) | (-11.89, 0.43) | (-10.08, 0.31) | (-3.24, 13.8) | (-3.63, 12.98) | (-4.89, 2.49) | (-3.27, 9.07) | (-15.73, 1.25) | (-3.32, 5.99) | (-3.45, 8.25) | (-8.4, 8.88) |
| 3.7 | 5.22 | 2.3 | 4.31 | 4.87 | -5.14 | -4.19 | 5.93 | 5.41 | -0.56 | 3.45 | -6.56 | 1.95 | 3.01 | 0.86 |
| (-5.97, 13.34) | (-3.05, 13.27) | (-3.47, 8.13) | (-2.69, 11.47) | (-2.14, 11.93) | (-13.36, 3) | (-11.77, 3.37) | (-4.11, 15.92) | (-4.59, 15.24) | (-7.15, 6.05) | (-4.58, 11.74) | (-16.57, 3.53) | (-5.18, 9.07) | (-4.93, 10.96) | (-9.44, 11.25) |
| 0.76 | 2.29 | -0.59 | 1.43 | 1.99 | -8.06 | -7.09 | 3.03 | 2.52 | -3.42 | 0.54 | -9.49 | -0.97 | 0.14 | -2.04 |
| (-8.99, 10.45) | (-6.17, 10.59) | (-6.9, 5.63) | (-5.69, 8.53) | (-5.12, 9.15) | (-16.36, 0.3) | (-14.85, 0.49) | (-7.13, 13.25) | (-7.45, 12.47) | (-10.3, 3.25) | (-7.65, 8.98) | (-19.7, 0.79) | (-8.27, 6.28) | (-7.91, 8.12) | (-12.24, 8.13) |
| 0.46 | 1.99 | -0.89 | 1.14 | 1.69 | **-8.34** | **-7.39** | 2.71 | 2.19 | -3.72 | 0.23 | **-9.76** | -1.26 | -0.17 | -2.34 |
| (-7.84, 8.87) | (-4.54, 8.54) | (-4.11, 2.35) | (-4, 6.28) | (-3.37, 6.74) | **(-14.83, -1.79)** | **(-13.18, -1.74)** | (-6.08, 11.58) | (-6.52, 10.78) | (-8.2, 0.65) | (-6.3, 6.94) | **(-18.58, -1)** | (-6.54, 4.01) | (-6.47, 6.12) | (-11.29, 6.59) |
| -0.6 | 0.89 | -1.98 | 0.02 | 0.59 | -9.41 | -8.42 | 1.63 | 1.16 | -4.79 | -0.84 | -10.86 | -2.37 | -1.23 | -3.41 |
| (-11.85, 10.47) | (-9.07, 10.96) | (-10.25, 6.31) | (-8.87, 9.21) | (-8.35, 9.53) | (-19.29, 0.54) | (-17.88, 0.92) | (-10.07, 13.16) | (-10.35, 12.49) | (-13.53, 3.89) | (-10.71, 9.14) | (-22.35, 0.84) | (-11.55, 6.88) | (-10.94, 8.44) | (-15.09, 8.2) |
| Lose weight | 1.48 | -1.34 | 0.64 | 1.23 | -8.82 | -7.85 | 2.26 | 1.76 | -4.2 | -0.25 | -10.29 | -1.76 | -0.64 | -2.82 |
|  | (-8.28, 11.19) | (-9.34, 6.55) | (-8.12, 9.36) | (-7.52, 9.85) | (-18.55, 0.79) | (-16.98, 1.32) | (-9.24, 13.45) | (-9.45, 12.94) | (-12.59, 4.03) | (-9.82, 9.68) | (-21.72, 1.1) | (-10.75, 7.15) | (-10.09, 8.65) | (-14.13, 8.63) |
| -1.34 | MBSR | -2.84 | -0.88 | -0.33 | **-10.32** | **-9.36** | 0.75 | 0.26 | -5.69 | -1.73 | **-11.71** | -3.25 | -2.17 | -4.29 |
| (-16.11, 13.48) |  | (-8.86, 3.21) | (-7.76, 6.2) | (-7.22, 6.77) | **(-18.42, -2.04)** | **(-16.99, -1.82)** | (-9.36, 11) | (-9.91, 10.01) | (-12.3, 0.91) | (-9.78, 6.56) | **(-21.99, -1.5)** | (-10.47, 4.08) | (-10.02, 5.84) | (-14.58, 5.9) |
| -2.94 | -1.55 | LSM | 2.02 | 2.58 | **-7.45** | **-6.5** | 3.63 | 3.1 | -2.86 | 1.14 | **-8.89** | -0.41 | 0.73 | -1.44 |
| (-15.14, 9.27) | (-10.65, 7.5) |  | (-2.34, 6.39) | (-1.75, 6.82) | **(-13.41, -1.37)** | **(-11.42, -1.7)** | (-4.84, 12) | (-5.17, 11.28) | (-6.15, 0.41) | (-4.87, 7.21) | **(-17.04, -0.7)** | (-4.78, 4.09) | (-5.03, 6.39) | (-9.97, 7.09) |
| -1.38 | -0.05 | 1.5 | TCM constitution intervention | 0.55 | **-9.47** | **-8.52** | 1.58 | 1.07 | -4.87 | -0.89 | **-10.9** | -2.39 | -1.32 | -3.5 |
| (-14.68, 12.03) | (-10.59, 10.42) | (-5.19, 8.12) |  | (-5.02, 6.12) | **(-16.42, -2.46)** | **(-14.77, -2.29)** | (-7.53, 10.67) | (-7.93, 9.96) | (-10.04, 0.22) | (-7.96, 6.32) | **(-20.18, -1.62)** | (-8.18, 3.48) | (-7.97, 5.39) | (-12.74, 5.78) |
| -1.25 | 0.09 | 1.65 | 0.15 | Remote guided LSM | **-10.03** | **-9.08** | 1.07 | 0.52 | **-5.43** | -1.43 | **-11.48** | -2.93 | -1.89 | -4.04 |
| (-14.28, 11.86) | (-10.29, 10.24) | (-4.32, 7.53) | (-8.14, 8.3) |  | **(-17.03, -2.9)** | **(-15.36, -2.83)** | (-8.14, 10.14) | (-8.47, 9.42) | **(-10.57, -0.37)** | (-8.32, 5.66) | **(-20.73, -1.99)** | (-8.72, 2.77) | (-8.44, 4.83) | (-13.27, 5.21) |
| -5.32 | -3.99 | -2.46 | -3.99 | -4.07 | AE+RE | 0.99 | 11.08 | 10.53 | 4.6 | 8.63 | -1.42 | 7.07 | 8.17 | 6.01 |
| (-19.93, 9.34) | (-16.27, 8.12) | (-11.36, 6.58) | (-14.5, 6.48) | (-14.2, 6.05) |  | (-6.67, 8.28) | (0.92, 21) | (0.61, 20.44) | (-2, 11.19) | (0.52, 16.67) | (-11.61, 8.68) | (-0.18, 14.26) | (0.2, 15.99) | (-4.24, 16.16) |
| -8.14 | -6.84 | -5.26 | -6.79 | -6.91 | -2.84 | LSM+drug | 10.1 | 9.58 | 3.66 | 7.65 | -2.33 | 6.16 | 7.2 | 5.08 |
| (-22.36, 6.02) | (-18.49, 4.74) | (-12.7, 2.37) | (-16.3, 2.95) | (-16.1, 2.49) | (-14.16, 8.57) |  | (0.54, 19.66) | (0.19, 19.03) | (-2.07, 9.4) | (0.22, 15.3) | (-11.87, 7.22) | (-0.22, 12.49) | (0, 14.5) | (-4.69, 14.76) |
| -7.49 | -6.35 | -4.65 | -6.23 | -6.33 | -2.23 | 0.6 | Baduanjin+Acupoint | -0.47 | -6.48 | -2.47 | **-12.53** | -3.99 | -2.92 | -5.03 |
| (-24.23, 9.59) | (-20.99, 8.65) | (-16.9, 7.69) | (-19.5, 7.44) | (-19.41, 6.79) | (-16.85, 12.49) | (-13.56, 14.86) |  | (-12.09, 10.95) | (-15.29, 2.44) | (-12.35, 7.57) | **(-24.22, -0.64)** | (-13.31, 5.3) | (-12.82, 7.08) | (-16.91, 6.75) |
| 1.48 | 2.79 | 4.32 | 2.84 | 2.66 | 6.77 | 9.52 | 9.02 | AE+Acupoint | -5.96 | -1.9 | **-12.03** | -3.47 | -2.39 | -4.61 |
| (-15.21, 18.35) | (-12.01, 17.59) | (-8.07, 16.69) | (-10.71, 16.24) | (-10.49, 16.11) | (-7.94, 21.78) | (-4.43, 24.08) | (-7.95, 25.95) |  | (-14.56, 2.84) | (-11.71, 7.85) | **(-23.42, -0.23)** | (-12.65, 5.67) | (-12.16, 7.49) | (-16.02, 7.19) |
| -9.71 | -8.26 | **-6.71** | **-8.24** | **-8.42** | -4.29 | -1.47 | -2.07 | -11.08 | ARB | 3.97 | -6.02 | 2.47 | 3.59 | 1.42 |
| (-22.62, 3.46) | (-18.35, 1.71) | **(-11.85, -1.72)** | **(-16.1, -0.42)** | **(-15.74, -0.92)** | (-14.21, 5.49) | (-10.3, 7.36) | (-15.21, 10.8) | (-24.19, 1.88) |  | (-2.52, 10.72) | (-14.88, 2.85) | (-2.03, 7.08) | (-2.71, 9.9) | (-7.46, 10.59) |
| 0.37 | 1.74 | 3.21 | 1.73 | 1.57 | 5.69 | 8.51 | 7.94 | -1.14 | 9.95 | Beta blocker | -10.05 | -1.53 | -0.42 | -2.62 |
| (-14.55, 15.19) | (-10.55, 14.13) | (-5.92, 12.46) | (-8.94, 12.41) | (-8.7, 12.05) | (-6.86, 17.97) | (-3.17, 20.05) | (-6.92, 22.71) | (-15.96, 13.85) | (0, 20.11) |  | (-20.15, 0.1) | (-8.77, 5.6) | (-8.41, 7.47) | (-12.92, 7.48) |
| **-20.7** | **-19.44** | **-17.91** | **-19.42** | **-19.55** | **-15.4** | -12.62 | -13.25 | **-22.31** | -11.17 | **-21.08** | CCB | 8.49 | 9.61 | 7.45 |
| **(-38.24, -3.21)** | **(-34.9, -4.11)** | **(-30.36, -5.41)** | **(-33.54, -5.2)** | **(-33.27, -5.72)** | **(-30.8, -0.19)** | (-27.1, 1.89) | (-30.85, 4.4) | **(-39.72, -4.82)** | (-24.53, 2.19) | **(-36.61, -5.82)** |  | (-0.71, 17.83) | (-0.3, 19.58) | (-4.54, 19.08) |
| -5.05 | -3.71 | -2.12 | -3.66 | -3.81 | 0.34 | 3.13 | 2.52 | -6.46 | 4.61 | -5.38 | 15.73 | Diuretic | 1.11 | -1.07 |
| (-18.59, 8.7) | (-14.6, 7.14) | (-8.82, 4.62) | (-12.45, 5.33) | (-12.31, 4.92) | (-10.41, 10.97) | (-6.6, 13) | (-11.16, 15.96) | (-20.16, 7.36) | (-2.46, 11.65) | (-16.38, 5.49) | (1.62, 29.81) |  | (-5.8, 8.02) | (-10.39, 8.38) |
| -2.44 | -1.06 | 0.46 | -1.02 | -1.21 | 2.9 | 5.81 | 5.14 | -3.85 | 7.2 | -2.83 | 18.29 | 2.54 | Statins | -2.19 |
| (-16.66, 11.83) | (-12.83, 10.29) | (-7.73, 8.66) | (-10.95, 8.74) | (-10.72, 8.3) | (-8.86, 14.42) | (-5.18, 16.46) | (-9.54, 19.28) | (-18.43, 10.32) | (-1.98, 16.46) | (-14.63, 8.89) | (3.6, 33.33) | (-7.57, 12.7) |  | (-12.28, 7.84) |
| -3.06 | -1.77 | -0.2 | -1.71 | -1.81 | 2.25 | 5.06 | 4.43 | -4.49 | 6.57 | -3.42 | 17.68 | 1.94 | -0.71 | Allopurinol |
| (-21.15, 15.04) | (-17.74, 14.31) | (-14.02, 13.49) | (-16.68, 13.2) | (-16.46, 12.79) | (-13.76, 18.03) | (-10.43, 20.5) | (-13.79, 22.69) | (-22.92, 13.36) | (-7.74, 20.89) | (-19.5, 12.55) | (-0.99, 36.22) | (-13.22, 16.91) | (-16.17, 14.96) |  |

Network meta-analysis of the efficacy of reducing SBP (lower triangle) and DBP (upper triangle)

The superiority estimate (one intervention is superior to the other) for each pairwise comparison lies at the intersection between the rows and columns, with data above 0 and above a 95% CI of 1 in favour of defining the treatment effect, indicating that the intervention in the column is superior to the row. Comparisons with differences are indicated by bolded font and the magnitude of the difference is presented by the shade of colour.

CI: confidence interval; RR: risk ratio; SUCRA: surface under the cumulative ranking.

AE, aerobic exercise; HIIT, high-intensity interval training; RE, resistance exercise; DASH, dietary approaches to stop hypertension; TPM, Traditional Persian Medicine; TCD, traditional Chinese drug; EMG, electromyographic; MBSR, mindfulness-based stress reduction; LSM, lifestyle modification; TCM, Traditional Chinese Medicine; ARB, angiotensin II receptor blockers; ACE, angiotensin-converting enzyme inhibitors; CCB, calcium channel blockers.

Supplementary Table 8. Network meta-analysis of HT progression and cardiac, cerebral, renal and mortality outcomes

| Control |  |  |  |  |  |  |  |  |  | 0.16 |  | 0.23 |  | 0.89 | 0.55 |
| --- | --- | --- | --- | --- | --- | --- | --- | --- | --- | --- | --- | --- | --- | --- | --- |
|  |  |  |  |  |  |  |  |  |  | (0.01, 1.14) |  | (0.01, 3.54) |  | (0.06, 14.34) | (0.04, 8.57) |
| **0.16** | DASH |  |  |  |  |  |  |  |  |  |  |  |  |  |  |
| **(0.03, 0.8)** |  |  |  |  |  |  |  |  |  |  |  |  |  |  |  |
| 0.66 | 4.18 | Salt restriction |  |  |  |  |  |  |  |  |  |  |  |  |  |
| (0.15, 3.06) | (0.47, 45.64) |  |  |  |  |  |  |  |  |  |  |  |  |  |  |
| 0.47 | 2.94 | 0.7 | Potassium supplements |  |  |  |  |  |  |  |  |  |  |  |  |
| (0.12, 1.77) | (0.38, 28.89) | (0.1, 5.34) |  |  |  |  |  |  |  |  |  |  |  |  |  |
| 0.06 | 0.35 | 0.08 | 0.12 | Medicinal herbs Taking |  |  |  |  |  |  |  |  |  |  |  |
| (0, 1.09) | (0.01, 11.32) | (0, 2.28) | (0, 3.07) |  |  |  |  |  |  |  |  |  |  |  |  |
| 0.37 | 2.36 | 0.57 | 0.8 | 6.67 | TCD bubble |  |  |  |  |  |  |  |  |  |  |
| (0.03, 3.82) | (0.13, 44.36) | (0.03, 9.05) | (0.05, 11.85) | (0.14, 319.02) |  |  |  |  |  |  |  |  |  |  |  |
| **0.15** | 0.97 | 0.23 | 0.33 | 2.76 | 0.41 | Acupoint therapy |  |  |  |  |  |  |  |  |  |
| **(0.03, 0.72)** | (0.11, 10.54) | (0.03, 2.06) | (0.04, 2.52) | (0.12, 76.57) | (0.03, 7.62) |  |  |  |  |  |  |  |  |  |  |
| 1.21 | 7.69 | 1.84 | 2.6 | 22.23 | 3.24 | 7.93 | Homeopathy |  |  |  |  |  |  |  |  |
| (0.11, 13.4) | (0.44, 152.66) | (0.11, 30.16) | (0.17, 38.69) | (0.47, 1139.5) | (0.11, 94.12) | (0.44, 138.98) |  |  |  |  |  |  |  |  |  |
| 0.45 | 2.88 | 0.69 | 0.97 | 8.24 | 1.22 | 2.98 | 0.37 | Lose weight |  |  |  |  |  |  |  |
| (0.05, 3.97) | (0.19, 47.77) | (0.05, 9.64) | (0.08, 12.29) | (0.2, 373.93) | (0.05, 30.46) | (0.2, 45.07) | (0.01, 9.67) |  |  |  |  |  |  |  |  |
| 1.08 | 6.78 | 1.64 | 2.3 | 19.25 | 2.89 | 7.04 | 0.88 | 2.34 | MBSR |  |  |  |  |  |  |
| (0.13, 9.05) | (0.46, 116.4) | (0.12, 22.42) | (0.18, 28.93) | (0.48, 841.99) | (0.12, 69.96) | (0.49, 96.3) | (0.04, 22.44) | (0.11, 51.12) |  |  |  |  |  |  |  |
| **0.25** | 1.59 | 0.38 | 0.55 | 4.48 | 0.68 | 1.64 | 0.21 | 0.56 | 0.24 | LSM |  | 1.43 |  | 5.38 | 3.36 |
| **(0.07, 0.87)** | (0.21, 13.99) | (0.05, 2.69) | (0.08, 3.27) | (0.31, 77.59) | (0.05, 10.52) | (0.31, 8.26) | (0.01, 3.21) | (0.04, 6.91) | (0.02, 2.94) |  |  | (0.07, 36.26) |  | (0.25, 342.8) | (0.16, 223.37) |
| 0.44 | 2.81 | 0.67 | 0.94 | 8.01 | 1.19 | 2.87 | 0.36 | 0.96 | 0.41 | 1.74 | Remote guided LSM |  |  |  |  |
| (0.08, 2.39) | (0.27, 31.98) | (0.07, 6.39) | (0.11, 8.06) | (0.26, 262.54) | (0.06, 22.41) | (0.28, 28.86) | (0.02, 6.79) | (0.06, 15.1) | (0.03, 6.46) | (0.21, 14.21) |  |  |  |  |  |
|  |  |  |  |  |  |  |  |  |  |  |  | LSM+drug |  | 3.89 | 2.45 |
|  |  |  |  |  |  |  |  |  |  |  |  |  |  | (0.09, 298.7) | (0.05, 206.65) |
| **0.14** | 0.85 | 0.21 | 0.29 | 2.42 | 0.36 | 0.88 | 0.11 | 0.3 | 0.13 | 0.54 | 0.31 |  | ARB |  |  |
| **(0.04, 0.41)** | (0.11, 6.99) | (0.03, 1.29) | (0.05, 1.62) | (0.09, 63.37) | (0.02, 5.26) | (0.11, 6) | (0.01, 1.55) | (0.02, 3.41) | (0.01, 1.44) | (0.09, 2.92) | (0.04, 2.32) |  |  |  |  |
| 0.59 | 3.74 | 0.89 | 1.26 | 10.57 | 1.58 | 3.87 | 0.48 | 1.3 | 0.55 | 2.34 | 1.34 |  | 4.36 | ACE | 0.62 |
| (0.07, 4.93) | (0.27, 63.45) | (0.06, 12.13) | (0.1, 15.87) | (0.29, 480.38) | (0.07, 39.73) | (0.27, 52.22) | (0.02, 12.03) | (0.06, 27.18) | (0.03, 12.08) | (0.21, 28.1) | (0.09, 20.91) |  | (0.42, 53.27) |  | (0.01, 31.03) |
| 0.54 | 3.41 | 0.82 | 1.16 | 9.59 | 1.44 | 3.54 | 0.44 | 1.2 | 0.51 | 2.14 | 1.23 |  | 3.98 | 0.92 | Diuretic |
| (0.09, 2.99) | (0.32, 41.37) | (0.08, 8.22) | (0.13, 10.08) | (0.32, 332.85) | (0.08, 27.87) | (0.33, 35.97) | (0.02, 8.83) | (0.07, 19.48) | (0.03, 7.99) | (0.25, 18.54) | (0.11, 13.99) |  | (0.52, 34.58) | (0.06, 13.66) |  |

Network meta-analysis of the efficacy of reducing HT progression (lower triangle) and cardiac, cerebral, renal and mortality outcomes (upper triangle)

The superiority estimate (one intervention is superior to the other) for each pairwise comparison lies at the intersection between the rows and columns, with data above 0 and above a 95% CI of 1 in favour of defining the treatment effect, indicating that the intervention in the column is superior to the row. Comparisons with differences are indicated by bolded font and the magnitude of the difference is presented by the shade of colour.

CI: confidence interval; RR: risk ratio; SUCRA: surface under the cumulative ranking.

AE, aerobic exercise; HIIT, high-intensity interval training; RE, resistance exercise; DASH, dietary approaches to stop hypertension; TPM, Traditional Persian Medicine; TCD, traditional Chinese drug; EMG, electromyographic; MBSR, mindfulness-based stress reduction; LSM, lifestyle modification; TCM, Traditional Chinese Medicine; ARB, angiotensin II receptor blockers; ACE, angiotensin-converting enzyme inhibitors; CCB, calcium channel blockers.

Supplementary Table 9. SCURA scores for interventions

| Interventions | SBP | DBP | HT  progression | Outcomes |
| --- | --- | --- | --- | --- |
| Control | 9.62% | 15.64% | 18.82% | 21.82% |
| AE | 74.71% | 82.95% |  |  |
| HIIT | 77.17% | 74.06% |  |  |
| RE | 51.40% | 70.92% |  |  |
| Yoga | 28.60% | 31.78% |  |  |
| DASH | 69.78% | 68.46% | 74.87% |  |
| TPM dietary habit | 56.65% | 59.98% |  |  |
| Salt restriction | 34.38% | 38.91% |  |  |
| Micronutrients | 42.82% | 26.52% | 34.03% |  |
| Food extract | 35.76% | 37.55% | 44.89% |  |
| Medicinal herbs Taking | 47.86% | 52.38% | 87.44% |  |
| TCD bubble | 73.74% | 54.76% | 51.46% |  |
| EMG | 46.10% | 46.30% |  |  |
| Acupoint therapy | 39.41% | 27.51% | 76.21% |  |
| Homeopathy | 32.90% | 36.38% | 22.59% |  |
| Lose weight | 32.67% | 41.17% | 46.39% |  |
| MBSR | 37.18% | 28.02% | 23.44% |  |
| LSM | 66.06% | 68.25% | 63.52% | 81.20% |
| TCM constitution intervention | 36.50% | 32.31% |  |  |
| Remote guided LSM | 28.86% | 27.45% | 46.60% |  |
| AE+RE | 61.04% | 93.33% |  |  |
| LSM+drug | 83.36% | 93.94% |  | 69.49% |
| Baduanjin+Acupoint | 70.97% | 25.89% |  |  |
| AE+Acupoint | 26.14% | 28.46% |  |  |
| ACE |  |  | 38.21% | 30.77% |
| ARB | 74.12% | 64.04% | 80.78% |  |
| Beta blocker | 28.39% | 41.37% |  |  |
| CCB | 92.84% | 83.18% |  |  |
| Diuretic | 47.12% | 41.86% | 40.74% | 46.73% |
| Statins | 45.43% | 46.09% |  |  |
| Allopurinol | 48.42% | 60.56% |  |  |

SUCRA, surface under the cumulative ranking; SBP, systolic blood pressure; DBP, diastolic blood pressure; HT, hypertension ;AE, aerobic exercise; HIIT, high-intensity interval training; RE, resistance exercise; DASH, dietary approaches to stop hypertension; TPM, Traditional Persian Medicine; TCD, traditional Chinese drug; EMG, electromyographic; MBSR, mindfulness-based stress reduction; LSM, lifestyle modification; TCM, Traditional Chinese Medicine; ARB, angiotensin II receptor blockers; ACE, angiotensin-converting enzyme inhibitors; CCB, calcium channel blockers.

Supplementary Table 10. CINeMA results for SBP

| Comparison | Number of studies | Within-study bias | Reporting bias | Indirectness | Imprecision | Heterogeneity | Incoherence | Confidence rating | Reason(s) for downgrading |
| --- | --- | --- | --- | --- | --- | --- | --- | --- | --- |
|  |  |  |  |  |  |  |  |  |  |
| Control: | 3 | No concerns | Low risk | No concerns | No concerns | Some concerns | No concerns | ⊕⊕⊕O | ["Heterogeneity"] |
| AE |  |  |  |  |  |  |  |  |  |
| Control: | 3 | No concerns | Low risk | No concerns | Major concerns | No concerns | Some concerns | ⊕OOO | ["Imprecision","Incoherence"] |
| RE |  |  |  |  |  |  |  |  |  |
| Control: | 2 | No concerns | Low risk | No concerns | No concerns | Some concerns | No concerns | ⊕⊕⊕O | ["Heterogeneity"] |
| DASH |  |  |  |  |  |  |  |  |  |
| Control: | 1 | Major concerns | Low risk | No concerns | Major concerns | No concerns | Some concerns | ⊕OOO | ["Within-study bias","Imprecision","Incoherence"] |
| TPM dietary habit |  |  |  |  |  |  |  |  |  |
| Control: | 3 | No concerns | Low risk | No concerns | Major concerns | No concerns | Some concerns | ⊕OOO | ["Imprecision","Incoherence"] |
| Salt restriction |  |  |  |  |  |  |  |  |  |
| Control: | 4 | No concerns | Low risk | No concerns | Major concerns | No concerns | No concerns | ⊕⊕OO | ["Imprecision"] |
| Micronutrients |  |  |  |  |  |  |  |  |  |
| Control: | 19 | No concerns | Low risk | No concerns | No concerns | Some concerns | No concerns | ⊕⊕⊕O | ["Heterogeneity"] |
| Food extract |  |  |  |  |  |  |  |  |  |
| Control: | 9 | No concerns | Low risk | No concerns | No concerns | Major concerns | No concerns | ⊕⊕OO | ["Heterogeneity"] |
| Medicinal herbs Taking |  |  |  |  |  |  |  |  |  |
| Control: | 1 | No concerns | Low risk | No concerns | No concerns | Some concerns | No concerns | ⊕⊕⊕O | ["Heterogeneity"] |
| TCD bubble |  |  |  |  |  |  |  |  |  |
| Control: | 2 | Some concerns | Low risk | No concerns | Major concerns | No concerns | Some concerns | ⊕OOO | ["Within-study bias","Imprecision","Incoherence"] |
| EMG |  |  |  |  |  |  |  |  |  |
| Control: | 4 | Some concerns | Low risk | No concerns | No concerns | Some concerns | Some concerns | ⊕OOO | ["Within-study bias","Heterogeneity","Incoherence"] |
| Acupoint therapy |  |  |  |  |  |  |  |  |  |
| Control: | 1 | No concerns | Low risk | No concerns | Major concerns | No concerns | Some concerns | ⊕OOO | ["Imprecision","Incoherence"] |
| Homeopathy |  |  |  |  |  |  |  |  |  |
| Control: | 1 | No concerns | Low risk | No concerns | Major concerns | No concerns | Some concerns | ⊕OOO | ["Imprecision","Incoherence"] |
| Lose weight |  |  |  |  |  |  |  |  |  |
| Control: | 2 | No concerns | Low risk | No concerns | Major concerns | No concerns | Some concerns | ⊕OOO | ["Imprecision","Incoherence"] |
| MBSR |  |  |  |  |  |  |  |  |  |
| Control: | 12 | No concerns | Low risk | No concerns | No concerns | Major concerns | Major concerns | ⊕OOO | ["Heterogeneity","Incoherence"] |
| LSM |  |  |  |  |  |  |  |  |  |
| Control: | 4 | Some concerns | Low risk | No concerns | Major concerns | No concerns | Some concerns | ⊕OOO | ["Within-study bias","Imprecision","Incoherence"] |
| TCM constitution intervention |  |  |  |  |  |  |  |  |  |
| Control: | 4 | No concerns | Low risk | No concerns | Major concerns | Some concerns | Some concerns | ⊕OOO | ["Imprecision","Heterogeneity","Incoherence"] |
| Remote guided LSM |  |  |  |  |  |  |  |  |  |
| Control: | 2 | No concerns | Low risk | No concerns | No concerns | Some concerns | No concerns | ⊕⊕⊕O | ["Heterogeneity"] |
| AE+RE |  |  |  |  |  |  |  |  |  |
| Control: | 2 | Some concerns | Low risk | No concerns | No concerns | Some concerns | No concerns | ⊕⊕OO | ["Within-study bias","Heterogeneity"] |
| LSM+drug |  |  |  |  |  |  |  |  |  |
| Control: | 1 | Major concerns | Low risk | No concerns | Major concerns | No concerns | Some concerns | ⊕OOO | ["Within-study bias","Imprecision","Incoherence"] |
| Baduanjin+Acupoint |  |  |  |  |  |  |  |  |  |
| Control: | 1 | No concerns | Low risk | No concerns | Major concerns | No concerns | Some concerns | ⊕OOO | ["Imprecision","Incoherence"] |
| AE+Acupoint |  |  |  |  |  |  |  |  |  |
| Control: | 3 | Some concerns | Low risk | No concerns | No concerns | No concerns | No concerns | ⊕⊕⊕O | ["Within-study bias"] |
| ARB |  |  |  |  |  |  |  |  |  |
| Control: | 2 | Major concerns | Low risk | No concerns | Major concerns | No concerns | Some concerns | ⊕OOO | ["Within-study bias","Imprecision","Incoherence"] |
| Beta blocker |  |  |  |  |  |  |  |  |  |
| Control: | 2 | Some concerns | Low risk | No concerns | No concerns | Some concerns | No concerns | ⊕⊕OO | ["Within-study bias","Heterogeneity"] |
| Diuretic |  |  |  |  |  |  |  |  |  |
| Control: | 3 | No concerns | Low risk | No concerns | Major concerns | No concerns | Some concerns | ⊕OOO | ["Imprecision","Incoherence"] |
| Statins |  |  |  |  |  |  |  |  |  |
| Control: | 1 | Major concerns | Low risk | No concerns | Major concerns | No concerns | Some concerns | ⊕OOO | ["Within-study bias","Imprecision","Incoherence"] |
| Allopurinol |  |  |  |  |  |  |  |  |  |
| Control: | 0 | No concerns | Low risk | No concerns | No concerns | Some concerns | Some concerns | ⊕⊕OO | ["Heterogeneity","Incoherence"] |
| HIIT |  |  |  |  |  |  |  |  |  |
| Control: | 0 | No concerns | Low risk | No concerns | Major concerns | No concerns | Some concerns | ⊕OOO | ["Imprecision","Incoherence"] |
| Yoga |  |  |  |  |  |  |  |  |  |
| Control: CCB | 0 | Some concerns | Low risk | No concerns | No concerns | No concerns | Some concerns | ⊕⊕OO | ["Within-study bias","Incoherence"] |

AE, aerobic exercise; HIIT, high-intensity interval training; RE, resistance exercise; DASH, dietary approaches to stop hypertension; TPM, Traditional Persian Medicine; TCD, traditional Chinese drug; EMG, electromyographic; MBSR, mindfulness-based stress reduction; LSM, lifestyle modification; TCM, Traditional Chinese Medicine; ARB, angiotensin II receptor blockers; ACE, angiotensin-converting enzyme inhibitors; CCB, calcium channel blockers.

⊕⊕⊕⊕, high quality; ⊕⊕⊕O, moderate quality; ⊕⊕OO, low quality; ⊕OOO, very low quality.

Supplementary Table 11. CINeMA results for DBP

| Comparison | Number of studies | Within-study bias | Reporting bias | Indirectness | Imprecision | Heterogeneity | Incoherence | Confidence rating | Reason(s) for downgrading |
| --- | --- | --- | --- | --- | --- | --- | --- | --- | --- |
|  |  |  |  |  |  |  |  |  |  |
| Control: | 3 | No concerns | Low risk | No concerns | No concerns | No concerns | No concerns | ⊕⊕⊕⊕ |  |
| AE |  |  |  |  |  |  |  |  |  |
| Control: | 3 | No concerns | Low risk | No concerns | No concerns | Some concerns | No concerns | ⊕⊕⊕O | ["Heterogeneity"] |
| RE |  |  |  |  |  |  |  |  |  |
| Control: | 2 | No concerns | Low risk | No concerns | No concerns | Some concerns | No concerns | ⊕⊕⊕O | ["Heterogeneity"] |
| DASH |  |  |  |  |  |  |  |  |  |
| Control: | 1 | Major concerns | Low risk | No concerns | Major concerns | No concerns | No concerns | ⊕OOO | ["Within-study bias","Imprecision"] |
| TPM dietary habit |  |  |  |  |  |  |  |  |  |
| Control: | 3 | No concerns | Low risk | No concerns | Major concerns | No concerns | No concerns | ⊕⊕OO | ["Imprecision"] |
| Salt restriction |  |  |  |  |  |  |  |  |  |
| Control: | 4 | No concerns | Low risk | No concerns | Major concerns | No concerns | No concerns | ⊕⊕OO | ["Imprecision"] |
| Micronutrients |  |  |  |  |  |  |  |  |  |
| Control: | 18 | No concerns | Low risk | No concerns | No concerns | Some concerns | No concerns | ⊕⊕⊕O | ["Heterogeneity"] |
| Food extract |  |  |  |  |  |  |  |  |  |
| Control: | 9 | No concerns | Low risk | No concerns | No concerns | Major concerns | No concerns | ⊕⊕OO | ["Heterogeneity"] |
| Medicinal herbs Taking |  |  |  |  |  |  |  |  |  |
| Control: | 1 | No concerns | Low risk | No concerns | No concerns | Some concerns | No concerns | ⊕⊕⊕O | ["Heterogeneity"] |
| TCD bubble |  |  |  |  |  |  |  |  |  |
| Control: | 2 | Some concerns | Low risk | No concerns | Major concerns | No concerns | No concerns | ⊕OOO | ["Within-study bias","Imprecision"] |
| EMG |  |  |  |  |  |  |  |  |  |
| Control: | 4 | Some concerns | Low risk | No concerns | Major concerns | No concerns | No concerns | ⊕OOO | ["Within-study bias","Imprecision"] |
| Acupoint therapy |  |  |  |  |  |  |  |  |  |
| Control: | 1 | No concerns | Low risk | No concerns | Major concerns | No concerns | No concerns | ⊕⊕OO | ["Imprecision"] |
| Homeopathy |  |  |  |  |  |  |  |  |  |
| Control: | 1 | No concerns | Low risk | No concerns | Major concerns | No concerns | No concerns | ⊕⊕OO | ["Imprecision"] |
| Lose weight |  |  |  |  |  |  |  |  |  |
| Control: | 2 | No concerns | Low risk | No concerns | Major concerns | No concerns | No concerns | ⊕⊕OO | ["Imprecision"] |
| MBSR |  |  |  |  |  |  |  |  |  |
| Control: | 12 | No concerns | Low risk | No concerns | No concerns | Major concerns | Major concerns | ⊕OOO | ["Heterogeneity","Incoherence"] |
| LSM |  |  |  |  |  |  |  |  |  |
| Control: | 4 | Some concerns | Low risk | No concerns | Major concerns | No concerns | No concerns | ⊕OOO | ["Within-study bias","Imprecision"] |
| TCM constitution intervention |  |  |  |  |  |  |  |  |  |
| Control: | 4 | No concerns | Low risk | No concerns | Major concerns | No concerns | No concerns | ⊕⊕OO | ["Imprecision"] |
| Remote guided LSM |  |  |  |  |  |  |  |  |  |
| Control: | 2 | No concerns | Low risk | No concerns | No concerns | No concerns | No concerns | ⊕⊕⊕⊕ |  |
| AE+RE |  |  |  |  |  |  |  |  |  |
| Control: | 2 | Some concerns | Low risk | No concerns | No concerns | No concerns | No concerns | ⊕⊕⊕O | ["Within-study bias"] |
| LSM+drug |  |  |  |  |  |  |  |  |  |
| Control: | 1 | Major concerns | Low risk | No concerns | Major concerns | No concerns | No concerns | ⊕OOO | ["Within-study bias","Imprecision"] |
| Baduanjin+Acupoint |  |  |  |  |  |  |  |  |  |
| Control: | 1 | No concerns | Low risk | No concerns | Major concerns | No concerns | No concerns | ⊕⊕OO | ["Imprecision"] |
| AE+Acupoint |  |  |  |  |  |  |  |  |  |
| Control: | 3 | Some concerns | Low risk | No concerns | No concerns | Some concerns | No concerns | ⊕⊕OO | ["Within-study bias","Heterogeneity"] |
| ARB |  |  |  |  |  |  |  |  |  |
| Control: | 2 | Major concerns | Low risk | No concerns | Major concerns | Some concerns | No concerns | ⊕OOO | ["Within-study bias","Imprecision","Heterogeneity"] |
| Beta blocker |  |  |  |  |  |  |  |  |  |
| Control: | 2 | Some concerns | Low risk | No concerns | No concerns | Some concerns | No concerns | ⊕⊕OO | ["Within-study bias","Heterogeneity"] |
| Diuretic |  |  |  |  |  |  |  |  |  |
| Control: | 3 | No concerns | Low risk | No concerns | Major concerns | No concerns | No concerns | ⊕⊕OO | ["Imprecision"] |
| Statins |  |  |  |  |  |  |  |  |  |
| Control: | 1 | Major concerns | Low risk | No concerns | Major concerns | No concerns | No concerns | ⊕OOO | ["Within-study bias","Imprecision"] |
| Allopurinol |  |  |  |  |  |  |  |  |  |
| Control: | 0 | No concerns | Low risk | No concerns | No concerns | Some concerns | No concerns | ⊕⊕⊕O | ["Heterogeneity"] |
| HIIT |  |  |  |  |  |  |  |  |  |
| Control: | 0 | No concerns | Low risk | No concerns | Major concerns | No concerns | No concerns | ⊕⊕OO | ["Imprecision"] |
| Yoga |  |  |  |  |  |  |  |  |  |
| Control: CCB | 0 | Some concerns | Low risk | No concerns | No concerns | No concerns | No concerns | ⊕⊕⊕O | ["Within-study bias"] |

AE, aerobic exercise; HIIT, high-intensity interval training; RE, resistance exercise; DASH, dietary approaches to stop hypertension; TPM, Traditional Persian Medicine; TCD, traditional Chinese drug; EMG, electromyographic; MBSR, mindfulness-based stress reduction; LSM, lifestyle modification; TCM, Traditional Chinese Medicine; ARB, angiotensin II receptor blockers; ACE, angiotensin-converting enzyme inhibitors; CCB, calcium channel blockers.

⊕⊕⊕O, moderate quality; ⊕⊕OO, low quality; ⊕OOO, very low quality.

Supplementary Table 12. CINeMA results for HT progression

| Comparison | Number of studies | Within-study bias | Reporting bias | Indirectness | Imprecision | Heterogeneity | Incoherence | Confidence rating | Reason(s) for downgrading |
| --- | --- | --- | --- | --- | --- | --- | --- | --- | --- |
|  |  |  |  |  |  |  |  |  |  |
| Control: | 2 | No concerns | Low risk | Some concerns | No concerns | Some concerns | No concerns | ⊕⊕OO | ["Indirectness","Heterogeneity"] |
| DASH |  |  |  |  |  |  |  |  |  |
| Control: | 2 | Some concerns | Low risk | Some concerns | Major concerns | No concerns | No concerns | ⊕OOO | ["Within-study bias", "Indirectness","Imprecision"] |
| Salt restriction |  |  |  |  |  |  |  |  |  |
| Control: | 3 | No concerns | Low risk | Some concerns | Major concerns | No concerns | No concerns | ⊕OOO | ["Indirectness","Imprecision"] |
| Micronutrients |  |  |  |  |  |  |  |  |  |
| Control: | 0 | No concerns | Low risk | No concerns | No concerns | Major concerns | No concerns | ⊕⊕OO | ["Heterogeneity"] |
| Medicinal herbs Taking |  |  |  |  |  |  |  |  |  |
| Control: | 1 | No concerns | Low risk | No concerns | Major concerns | No concerns | No concerns | ⊕⊕OO | ["Imprecision"] |
| TCD bubble |  |  |  |  |  |  |  |  |  |
| Acupoint therapy: | 2 | Some concerns | Low risk | No concerns | No concerns | Some concerns | No concerns | ⊕⊕OO | ["Within-study bias","Heterogeneity"] |
| Control |  |  |  |  |  |  |  |  |  |
| Control: | 1 | No concerns | Low risk | No concerns | Major concerns | No concerns | No concerns | ⊕⊕OO | ["Imprecision"] |
| Homeopathy |  |  |  |  |  |  |  |  |  |
| Control: | 1 | No concerns | Low risk | Some concerns | Major concerns | No concerns | No concerns | ⊕OOO | ["Indirectness","Imprecision"] |
| Lose weight |  |  |  |  |  |  |  |  |  |
| Control: | 1 | No concerns | Low risk | Some concerns | Major concerns | No concerns | No concerns | ⊕OOO | ["Indirectness","Imprecision"] |
| MBSR |  |  |  |  |  |  |  |  |  |
| Control: | 3 | Some concerns | Low risk | No concerns | No concerns | Some concerns | No concerns | ⊕⊕OO | ["Within-study bias","Heterogeneity"] |
| LSM |  |  |  |  |  |  |  |  |  |
| Control: | 2 | No concerns | Low risk | Some concerns | Major concerns | No concerns | No concerns | ⊕OOO | ["Indirectness","Imprecision"] |
| Remote guided LSM |  |  |  |  |  |  |  |  |  |
| Control: | 4 | Some concerns | Low risk | No concerns | No concerns | Some concerns | No concerns | ⊕⊕OO | ["Within-study bias","Heterogeneity"] |
| ARB |  |  |  |  |  |  |  |  |  |
| Control: | 1 | No concerns | Low risk | Some concerns | Major concerns | No concerns | No concerns | ⊕OOO | ["Indirectness","Imprecision"] |
| ACE |  |  |  |  |  |  |  |  |  |
| Control: | 2 | Some concerns | Low risk | No concerns | Major concerns | No concerns | No concerns | ⊕OOO | ["Within-study bias","Imprecision"] |
| Diuretic |  |  |  |  |  |  |  |  |  |

AE, aerobic exercise; HIIT, high-intensity interval training; RE, resistance exercise; DASH, dietary approaches to stop hypertension; TPM, Traditional Persian Medicine; TCD, traditional Chinese drug; EMG, electromyographic; MBSR, mindfulness-based stress reduction; LSM, lifestyle modification; TCM, Traditional Chinese Medicine; ARB, angiotensin II receptor blockers; ACE, angiotensin-converting enzyme inhibitors; CCB, calcium channel blockers.

⊕⊕OO, low quality; ⊕OOO, very low quality.

Supplementary Table 13. CINeMA results for cardiac, cerebral, renal and mortality outcomes

| Comparison | Number of studies | Within-study bias | Reporting bias | Indirectness | Imprecision | Heterogeneity | Incoherence | Confidence rating | Reason(s) for downgrading |
| --- | --- | --- | --- | --- | --- | --- | --- | --- | --- |
|  |  |  |  |  |  |  |  |  |  |
| Control: | 3 | Some concerns | Low risk | No concerns | Major concerns | No concerns | No concerns | ⊕OOO | ["Within-study bias","Imprecision"] |
| LSM |  |  |  |  |  |  |  |  |  |
| Control: | 1 | Major concerns | Low risk | No concerns | Major concerns | No concerns | No concerns | ⊕OOO | ["Within-study bias","Imprecision"] |
| LSM+drug |  |  |  |  |  |  |  |  |  |
| Control: | 1 | No concerns | Low risk | No concerns | Major concerns | No concerns | No concerns | ⊕⊕OO | ["Imprecision"] |
| ACE |  |  |  |  |  |  |  |  |  |
| Control: | 1 | Major concerns | Low risk | No concerns | No concerns | Major concerns | No concerns | ⊕OOO | ["Within-study bias","Heterogeneity"] |
| Diuretic |  |  |  |  |  |  |  |  |  |

AE, aerobic exercise; HIIT, high-intensity interval training; RE, resistance exercise; DASH, dietary approaches to stop hypertension; TPM, Traditional Persian Medicine; TCD, traditional Chinese drug; EMG, electromyographic; MBSR, mindfulness-based stress reduction; LSM, lifestyle modification; TCM, Traditional Chinese Medicine; ARB, angiotensin II receptor blockers; ACE, angiotensin-converting enzyme inhibitors; CCB, calcium channel blockers.

⊕⊕OO, low quality; ⊕OOO, very low quality.

Supplementary Table 14. Summary of findings on the efficacy of various interventions for the progression rate of HT

| Intervention | Direct comparisons/ Participant | Relative effect (95% CI) | Certainty of evidence (CINEMA) | SUCRA |
| --- | --- | --- | --- | --- |
| Medicinal herbs Taking | 2 RCTs;  160 participants | 0.06  (0, 1.02) | ⊕⊕OO | 87.44% |
| ARB | 4 RCTs;  2129 participants | 0.14  (0.04, 0.42) | ⊕⊕OO | 80.78% |
| Acupoint therapy | 3 RCTs;  256 participants | 0.15  (0.03, 0.73) | ⊕⊕OO | 76.21% |
| DASH | 2 RCTs;  332 participants | 0.16  (0.03, 0.82) | ⊕⊕OO | 74.87% |
| LSM | 2 RCTs;  520 participants | 0.25  (0.07, 0.88) | ⊕⊕OO | 63.52% |
| TCD bubble | 1 RCTs;  100 participants | 0.37  (0.03, 4) | ⊕⊕OO | 51.46% |
| Remote guided LSM | 3 RCTs;  1367 participants | 0.44  (0.08, 2.47) | ⊕OOO | 46.60% |
| Lose weight | 1 RCTs;  564 participants | 0.45  (0.05, 3.84) | ⊕OOO | 46.39% |
| Micronutrients | 3 RCTs;  1015 participants | 0.47  (0.12, 1.78) | ⊕OOO | 44.89% |
| Diuretic | 1 RCTs;  730 participants | 0.54  (0.09, 3.07) | ⊕OOO | 40.74% |
| ACE | 1 RCTs;  1008 participants | 0.6  (0.07, 5.02) | ⊕OOO | 38.21% |
| Salt restriction | 2 RCTs;  1136 participants | 0.66  (0.14, 3.01) | ⊕OOO | 34.03% |
| MBSR | 1 RCTs;  564 participants | 1.08  (0.12, 9.66) | ⊕OOO | 23.44% |
| Homeopathy | 1 RCTs;  84 participants | 1.22  (0.1, 13.78) | ⊕⊕OO | 22.59% |
| Control |  |  |  | 18.82% |

CI: confidence interval; RR: risk ratio; SUCRA: surface under the cumulative ranking.

DASH, dietary approaches to stop hypertension; TCD, traditional Chinese drug; MBSR, mindfulness-based stress reduction; LSM, lifestyle modification; ARB, angiotensin II receptor blockers; ACE, angiotensin-converting enzyme inhibitors.

⊕⊕OO, low quality; ⊕OOO, very low quality.

Supplementary Table 15. Summary of findings on the efficacy of various interventions for the progression rate of cardiac, cerebral and renal outcomes

| Intervention | Direct comparisons/ Participant | Relative effect (95% CI) | Certainty of evidence (CINEMA) | SUCRA |
| --- | --- | --- | --- | --- |
| LSM | 2 RCTs;  373 participants | 0.16 (0.01, 1.14) | ⊕OOO | 81.20% |
| LSM+drug | 1 RCTs;  834 participants | 0.23 (0.01, 3.54) | ⊕OOO | 69.49% |
| Diuretic | 1 RCTs;  730 participants | 0.55 (0.04, 8.57) | ⊕OOO | 46.73% |
| ACE | 1 RCTs;  1008 participants | 0.89 (0.06, 14.34) | ⊕⊕OO | 30.77% |
| Control |  | 1 |  | 21.82% |

CI: confidence interval; RR: risk ratio; SUCRA: surface under the cumulative ranking.
LSM, lifestyle modification; ACE, angiotensin-converting enzyme inhibitors.

⊕⊕OO, low quality; ⊕OOO, very low quality.

Supplementary Table 16. Sensitivity analysis based on omitted medium and low quality studies

|  | SBP | | | | DBP | | | |
| --- | --- | --- | --- | --- | --- | --- | --- | --- |
| Comparison | No. of study | Weighted mean difference (95% CrI) | I^2^ (Direct comparison) | P value | No. of study | Weighted mean difference  (95% CrI) | I^2^ (Direct comparison) | P value |
| AE | | | | | | | | |
| Overall analysis | 4 | -10.65(-17.12, -4.32) | 93.97 | 0.74 | 4 | -8.03 (-12.25, -3.69) | 93.96 | 0.53 |
| Omit middle and low quality researchs | 0 | -10.63 (-17.03, -4.08) | 93.99 | 0.77 | 0 | -8 (-12.43, -3.51) | 93.95 | 0.59 |
| RE | | | | | | | | |
| Overall analysis | 3 | -6.54(-13.87, 0.71) | 75.23 | NA | 3 | -6 (-11.24, -0.59) | 77.64 | NA |
| Omit middle and low quality researchs | 0 | -6.46 (-13.76, 0.86) | 75.49 | NA | 0 | -5.97 (-11.4, -0.51) | 78.11 | NA |
| DASH | | | | | | | | |
| Overall analysis | 2 | -9.21(-16.47, -1.67) | 97.58 | 0.80 | 2 | -5.81 (-10.8, -0.81) | 92.83 | 0.96 |
| Omit middle and low quality researchs | 0 | -9.29 (-16.6, -1.64) | 97.59 | 0.82 | 0 | -5.82 (-10.91, -0.67) | 92.84 | 0.96 |
| TPM dietary habit | | | | | | | | |
| Overall analysis | 1 | -7.13(-19.32, 5.19) | NA | NA | 1 | -3.9 (-12.01, 4.29) | NA | NA |
| Omit middle and low quality researchs | 0 | -7.09 (-19.3, 5.11) | NA | NA | 0 | -3.81 (-12.12, 4.72) | NA | NA |
| Salt restriction | | | | | | | | |
| Overall analysis | 2 | -3.86(-10.95, 3.15) | 99.90 | NA | 2 | -2.18 (-6.91, 2.46) | 99.75 | NA |
| Omit middle and low quality researchs | 1 | -6.17 (-15.05, 2.74) | 98.33 | NA | 1 | -3.57 (-9.86, 2.45) | 97.70 | NA |
| Micronutrients | | | | | | | | |
| Overall analysis | 2 | -3.27(-8.99, 2.61) | 94.26 | NA | 2 | 0.46 (-3.41, 4.37) | 67.12 | NA |
| Omit middle and low quality researchs | 0 | -3.23 (-9.09, 2.55) | 94.28 | NA | 0 | 0.43 (-3.6, 4.46) | 67.33 | NA |
| Food extract | | | | | | | | |
| Overall analysis | 19 | -4.27(-7.24, -1.31) | 87.54 | NA | 18 | -2.06 (-4.08, -0.06) | 80.72 | NA |
| Omit middle and low quality researchs | 1 | -4.33 (-7.47, -1.1) | 86.69 | NA | 1 | -2.16 (-4.41, 0.06) | 74.89 | NA |
| Medicinal herbs Taking | | | | | | | | |
| Overall analysis | 9 | -8.51(-11.71, -5.33) | 94.40 | 0.01 | 9 | -5.24 (-7.42, -3.09) | 92.99 | 0.01 |
| Omit middle and low quality researchs | 1 | -8.91 (-12.56, -5.31) | 95.68 | 0.02 | 1 | -5.84 (-8.27, -3.44) | 93.87 | 0.05 |
| TCD bubble | | | | | | | | |
| Overall analysis | 1 | -13.44(-22, -4.64) | NA | 0.36 | 1 | -5.87 (-11.7, -0.08) | NA | 0.24 |
| Omit middle and low quality researchs | 1 | -13.14 (-22.06, -4.45) | NA | 0.40 | 1 | -5.65 (-11.67, 0.45) | NA | 0.28 |
| EMG | | | | | | | | |
| Overall analysis | 2 | -5.6(-14.46, 3.32) | 13.76 | NA | 2 | -2.98 (-8.93, 3) | 0.00 | NA |
| Omit middle and low quality researchs | 1 | -6.92 (-19.28, 5.63) | NA | NA | 1 | -2.61 (-11.07, 5.9) | NA | NA |
| Acupoint therapy | | | | | | | | |
| Overall analysis | 4 | -7.3(-12.1, -2.57) | 93.67 | 0.60 | 4 | -2.69 (-5.86, 0.51) | 88.92 | 0.71 |
| Omit middle and low quality researchs | 2 | -5.39 (-11.29, 0.46) | 68.09 | 0.20 | 2 | -1.6 (-5.5, 2.35) | 83.46 | 0.29 |
| Homeopathy | | | | | | | | |
| Overall analysis | 1 | -2.72(-14.95, 9.27) | NA | NA | 1 | -1.58 (-9.68, 6.49) | NA | NA |
| Omit middle and low quality researchs | 0 | -2.84 (-15.12, 9.47) | NA | NA | 0 | -1.61 (-9.95, 6.79) | NA | NA |
| Lose weight | | | | | | | | |
| Overall analysis | 1 | -2.82(-14.79, 9.11) | NA | NA | 1 | -2.2 (-9.9, 5.64) | NA | NA |
| Omit middle and low quality researchs | 0 | -2.93 (-14.97, 9.16) | NA | NA | 0 | -2.24 (-10.12, 5.8) | NA | NA |
| MBSR | | | | | | | | |
| Overall analysis | 2 | -4.23(-12.9, 4.44) | 96.29 | NA | 2 | -0.71 (-6.49, 5.07) | 0.00 | NA |
| Omit middle and low quality researchs | 0 | -4.25 (-13.04, 4.5) | 96.31 | NA | 0 | -0.76 (-6.81, 5.17) | 0.00 | NA |
| LSM | | | | | | | | |
| Overall analysis | 9 | -5.77(-8.44, -3.12) | 97.87 | 0.01 | 9 | -3.56 (-5.33, -1.79) | 95.29 | 0.01 |
| Omit middle and low quality researchs | 1 | -5.23 (-8.41, -2.08) | 98.41 | 0.00 | 1 | -3.3 (-5.42, -1.2) | 96.34 | 0.01 |
| TCM constitution intervention | | | | | | | | |
| Overall analysis | 4 | -4.27(-10.44, 1.8) | 98.48 | NA | 4 | -1.54 (-5.56, 2.46) | 95.26 | NA |
| Omit middle and low quality researchs | 1 | -6.25 (-13.28, 0.66) | 96.45 | NA | 1 | -2.73 (-7.44, 1.94) | 36.78 | NA |
| Remote guided LSM | | | | | | | | |
| Overall analysis | 4 | -4.1(-9.67, 1.35) | 93.73 | 0.00 | 4 | -0.99 (-4.92, 2.91) | 94.97 | NA |
| Omit middle and low quality researchs | 2 | -2.22 (-9.25, 4.91) | 95.42 | NA | 2 | -1.24 (-5.98, 3.54) | 96.75 | NA |
| AE+RE | | | | | | | | |
| Overall analysis | 2 | -8.23(-16.72, 0.38) | 17.03 | NA | 2 | -11.02 (-16.73, -5.21) | 87.27 | NA |
| Omit middle and low quality researchs | 0 | -8.2 (-16.68, 0.58) | 18.52 | NA | 0 | -10.93 (-16.85, -5) | 87.64 | NA |
| LSM + drug | | | | | | | | |
| Overall analysis | 2 | -11.07(-18.5, -3.49) | 92.22 | NA | 2 | -10.08 (-14.93, -5.28) | 97.89 | NA |
| Omit middle and low quality researchs | 1 | -12.5 (-23.04, -1.81) | NA | NA | 1 | -13.33 (-20.41, -6.18) | NA | NA |
| Baduanjin + Acupoint | | | | | | | | |
| Overall analysis | 1 | -10.48(-22.33, 1.6) | NA | NA | 1 | 0.02 (-8.28, 8.35) | NA | NA |
| Omit middle and low quality researchs | 1 |  |  |  | 1 |  |  |  |
| AE + Acupoint | | | | | | | | |
| Overall analysis | 1 | -1.42(-13.45, 10.76) | NA | NA | 1 | -0.47 (-8.58, 7.51) | NA | NA |
| Omit middle and low quality researchs | 0 | -1.34 (-13.53, 10.73) | NA | NA | 0 | -0.44 (-8.67, 7.75) | NA | NA |
| ARB | | | | | | | | |
| Overall analysis | 3 | -12.5(-17.59, -7.54) | 99.42 | 0.08 | 3 | -6.42 (-9.69, -3.26) | 94.95 | 0.30 |
| Omit middle and low quality researchs | 2 | -8.67 (-15.27, -2.16) | NA | 0.91 | 2 | -5.49 (-9.79, -1.13) | NA | 0.79 |
| Beta blocker | | | | | | | | |
| Overall analysis | 2 | -2.56(-11.32, 6.31) | 94.71 | NA | 2 | -2.42 (-8.09, 3.44) | 97.36 | NA |
| Omit middle and low quality researchs | 2 |  |  |  | 2 |  |  |  |
| Diuretic | | | | | | | | |
| Overall analysis | 1 | -7.92(-14.44, -1.24) | 97.76 | 0.24 | 1 | -3.95 (-8.24, 0.38) | 97.60 | 0.44 |
| Omit middle and low quality researchs | 1 | -7.48 (-15.79, 0.73) | NA | 0.85 | 1 | -4.68 (-10.2, 0.8) | NA | 0.98 |
| Statins | | | | | | | | |
| Overall analysis | 3 | -5.32(-13.06, 2.41) | 0.00 | NA | 3 | -2.85 (-8.27, 2.53) | 0.00 | NA |
| Omit middle and low quality researchs | 0 | -5.29 (-13.16, 2.55) | 0.00 | NA | 0 | -2.86 (-8.39, 2.73) | 0.00 | NA |
| Allopurinol | | | | | | | | |
| Overall analysis | 1 | -5.95(-19.53, 7.46) | NA | NA | 1 | -5.03 (-13.31, 3.32) | NA | NA |
| Omit middle and low quality researchs | 1 |  |  |  | 1 |  |  |  |

CI: confidence interval; RR: risk ratio; SUCRA: surface under the cumulative ranking.

AE, aerobic exercise; RE, resistance exercise; DASH, dietary approaches to stop hypertension; TPM, Traditional Persian Medicine; TCD, traditional Chinese drug; EMG, electromyographic; MBSR, mindfulness-based stress reduction; LSM, lifestyle modification; TCM, Traditional Chinese Medicine; ARB, angiotensin II receptor blockers; ACE, angiotensin-converting enzyme inhibitors.

Supplementary Table 17. Subgroups and sensitivity analysis of SBP and DBP based on prolonged intervention and different ethnic and cultural backgrounds (Asian, Caucasian)

|  | SBP | | | | DBP | | | |
| --- | --- | --- | --- | --- | --- | --- | --- | --- |
| Comparison | No. of study | Weighted mean difference (95% CrI) | I^2^(direct comparison) | P value | No. of study | Weighted mean difference  (95% CrI) | I^2^ (direct comparison) | P value |
| **AE** | | | | | | | | |
| Overall analysis | 3 | -10.65 (-17.12, -4.32) | 93.97 | 0.74 | 3 | -8.03 (-12.25, -3.69) | 93.96 | 0.53 |
| 12 months and above | 0 |  |  |  | 0 |  |  |  |
| Asians | 3 | -10.62 (-17.39, -3.85) | 93.98 | 0.78 | 3 | -7.84 (-12.53, -3.1) | 93.96 | 0.43 |
| CaucAsianss | 0 |  |  |  | 0 |  |  |  |
| **HIIT** | | | | | | | | |
| Overall analysis | 0 | -12.46 (-22.42, -2.39) |  |  | 0 | -7.46 (-14.89, -0.13) |  |  |
| 12 months and above | 0 |  |  |  | 0 |  |  |  |
| Asians | 0 | -13.19 (-26.05, -0.39) |  |  | 0 | -9.03 (-18.16, 0.33) |  |  |
| CaucAsianss | 0 |  |  |  | 0 |  |  |  |
| **RE** | | | | | | | | |
| Overall analysis | 3 | -6.54 (-13.87, 0.71) | 75.23 | NA | 3 | -6 (-11.24, -0.59) | 77.64 | NA |
| 12 months and above | 1 | -8.58 (-26.54, 9.56) | NA | NA | 1 | -7.84 (-19.66, 3.92) | NA | NA |
| Asians | 1 | -1.49 (-15.31, 12.57) | NA | NA | 1 | 0.73 (-10.94, 12.52) | NA | NA |
| CaucAsianss | 2 | -8.74 (-14.66, -2.83) | 0 | NA | 2 | -7.92 (-12.43, -3.47) | 0 | NA |
| **Yoga** | | | | | | | | |
| Overall analysis | 1 | -7.73 (-20.29, 4.44) |  |  | 1 | -4.6 (-12.63, 3.52) |  |  |
| 12 months and above | 0 |  |  |  | 0 |  |  |  |
| Asians | 0 | -7.73 (-20.92, 5.64) |  |  | 0 | -4.21 (-13.13, 4.58) |  |  |
| CaucAsianss | 0 |  |  |  | 0 |  |  |  |
| **DASH** | | | | | | | | |
| Overall analysis | 2 | -9.21 (-16.47, -1.67) | 97.58 | 0.80 | 2 | -5.81 (-10.8, -0.81) | 92.83 | 0.96 |
| 12 months and above | 1 | -17.38 (-35.14, 0.23) | NA | NA | 1 | -8.8 (-19.6, 1.98) | NA | NA |
| Asians | 2 | -9.36 (-17.28, -1.32) | 97.58 | 0.86 | 2 | -6.06 (-11.51, -0.6) | 92.94 | 0.83 |
| CaucAsianss | 0 |  |  |  | 0 |  |  |  |
| **TPM dietary habit** | | | | | | | | |
| Overall analysis | 1 | -7.13(-19.32, 5.19) | NA | NA | 1 | -3.9 (-12.01, 4.29) | NA | NA |
| 12 months and above | 0 |  |  |  | 0 |  |  |  |
| Asians | 0 |  |  |  | 0 |  |  |  |
| CaucAsianss | 1 | -7.06 (-15.18, 1.05) | NA | NA | 1 | -3.85 (-9.8, 2.07) | NA | NA |
| **Salt restriction** | | | | | | | | |
| Overall analysis | 3 | -3.86 (-10.95, 3.15) | 99.90 | NA | 3 | -2.18 (-6.91, 2.46) | 99.75 | NA |
| 12 months and above | 1 | 0.3 (-17.18, 17.57) | NA | NA | 1 | 0.07 (-10.11, 10.43) | NA | NA |
| Asians | 0 |  |  |  | 0 |  |  |  |
| CaucAsianss | 3 | -3.16 (-8.01, 1.15) | 99.86 | NA | 3 | -1.66 (-5.33, 1.52) | 99.60 | NA |
| **Micronutrients** | | | | | | | | |
| Overall analysis | 3 | -3.27 (-8.99, 2.61) | 94.26 | NA | 3 | 0.46 (-3.41, 4.37) | 67.12 | NA |
| 12 months and above | 2 | -0.32 (-12.67, 12.14) | 0.00 | NA | 2 | 0.01 (-7.15, 7.36) | 0.00 | NA |
| Asians | 0 |  |  |  | 0 |  |  |  |
| CaucAsianss | 1 | -0.31 (-5.68, 5.03) | 0.00 | NA | 1 | 0.07 (-3.82, 3.95) | 0.00 | NA |
| **Food extract** | | | | | | | | |
| Overall analysis | 19 | -4.27 (-7.24, -1.31) | 87.54 | NA | 18 | -2.06 (-4.08, -0.06) | 80.72 | NA |
| 12 months and above | 0 |  |  |  | 0 |  |  |  |
| Asians | 7 | -5.12 (-10.58, 0.22) | 0.00 | NA | 7 | -3.31 (-6.99, 0.39) | 73.66 | NA |
| CaucAsianss | 10 | -3.96 (-6.47, -1.51) | 93.49 | NA | 9 | -1.88 (-3.78, 0.05) | 87.98 | NA |
| **Medicinal herbs Taking** | | | | | | | | |
| Overall analysis | 17 | -8.51 (-11.71, -5.33) | 94.40 | 0.01 | 17 | -5.24 (-7.42, -3.09) | 92.99 | 0.01 |
| 12 months and above | 3 | -6.62 (-16.75, 3.45) | 83.23 | NA | 3 | -3.35 (-9.18, 2.8) | 72.24 | NA |
| Asians | 8 | -7.74 (-11.37, -4.12) | 78.54 | 0.00 | 8 | -4.69 (-7.13, -2.23) | 93.07 | 0.01 |
| CaucAsianss | 1 | -21.71 (-29.82, -13.61) | NA | NA | 1 | -10.68 (-16.55, -4.88) | NA | NA |
| **TCD bubble** | | | | | | | | |
| Overall analysis | 2 | -13.44 (-22, -4.64) | NA | 0.36 | 2 | -5.87 (-11.7, -0.08) | NA | 0.24 |
| 12 months and above | 0 |  |  |  | 0 |  |  |  |
| Asians | 1 | -13.52 (-22.63, -4.11) | NA | 0.38 | 1 | -5.65 (-11.89, 0.81) | NA | 0.31 |
| CaucAsianss | 0 |  |  |  | 0 |  |  |  |
| **EMG** | | | | | | | | |
| Overall analysis | 2 | -5.6 (-14.46, 3.32) | 13.76 | NA | 2 | -2.98 (-8.93, 3) | 0.00 | NA |
| 12 months and above | 0 |  |  |  | 0 |  |  |  |
| Asians | 2 | -5.59 (-14.94, 3.83) | 13.95 | NA | 2 | -2.96 (-9.57, 3.51) | 0.00 | NA |
| CaucAsianss | 0 |  |  |  | 0 |  |  |  |
| **Acupoint therapy** | | | | | | | | |
| Overall analysis | 7 | -7.3 (-12.1, -2.57) | 93.67 | 0.60 | 7 | -2.69 (-5.86, 0.51) | 88.92 | 0.71 |
| 12 months and above | 1 | -13.45 (-32.2, 5.09) | NA | NA | 1 | -6.74 (-17.42, 4.26) | NA | NA |
| Asians | 4 | -7.29 (-12.48, -2.21) | 93.69 | 0.61 | 4 | -2.5 (-5.98, 0.96) | 88.85 | 0.84 |
| CaucAsianss | 0 |  |  |  | 0 |  |  |  |
| **Homeopathy** | | | | | | | | |
| Overall analysis | 1 | -2.72 (-14.95, 9.27) | NA | NA | 1 | -1.58 (-9.68, 6.49) | NA | NA |
| 12 months and above | 0 |  |  |  | 0 |  |  |  |
| Asians | 1 | -2.73 (-16.08, 10.5) | NA | NA | 1 | -1.58 (-10.46, 7.31) | NA | NA |
| CaucAsianss | 0 |  |  |  | 0 |  |  |  |
| **Lose weight** | | | | | | | | |
| Overall analysis | 1 | -2.82 (-14.79, 9.11) | NA | NA | 1 | -2.2 (-9.9, 5.64) | NA | NA |
| 12 months and above | 1 | -2.76 (-20.19, 14.68) | 98.12 | 0.68 | 1 | -2.27 (-12.65, 8.08) | 95.50 | 0.40 |
| Asians | 0 |  |  |  | 0 |  |  |  |
| CaucAsianss | 1 | -2.96 (-10.46, 4.59) | NA | NA | 1 | -2.24 (-7.7, 3.22) | NA | NA |
| **MBSR** | | | | | | | | |
| Overall analysis | 2 | -4.23 (-12.9, 4.44) | 96.29 | NA | 2 | -0.71 (-6.49, 5.07) | 0.00 | NA |
| 12 months and above | 0 |  |  |  | 0 |  |  |  |
| Asians | 1 | -8.46 (-22.21, 5.27) | NA | NA | 1 | -0.63 (-10.25, 8.96) | NA | NA |
| CaucAsianss | 1 | -0.51 (-8.1, 6.95) | NA | NA | 1 | -0.82 (-6.33, 4.72) | NA | NA |
| **LSM** | | | | | | | | |
| Overall analysis | 9 | -5.77 (-8.44, -3.12) | 97.87 | 0.01 | 9 | -3.56 (-5.33, -1.79) | 95.29 | 0.01 |
| 12 months and above | 7 | -7.36 (-12.59, -2.12) | 99.00 | NA | 7 | -4.18 (-7.27, -1.07) | 97.05 | NA |
| Asians | 8 | -5.79 (-8.81, -2.75) | 98.01 | 0.02 | 8 | -3.18 (-5.2, -1.14) | 95.27 | 0.03 |
| CaucAsianss | 0 | 7.45 (-2.98, 17.93) |  |  | 0 | -2.7 (-12.1, 6.83) |  |  |
| **TCM constitution intervention** | | | | | | | | |
| Overall analysis | 4 | -4.27 (-10.44, 1.8) | 98.48 | NA | 4 | -1.54 (-5.56, 2.46) | 95.26 | NA |
| 12 months and above | 3 | -3.88 (-13.91, 5.98) | 59.25 | NA | 3 | -0.8 (-6.71, 5.01) | 89.62 | NA |
| Asians | 4 | -4.33 (-10.77, 2.23) | 98.48 | NA | 4 | -1.54 (-5.91, 2.76) | 95.27 | NA |
| CaucAsianss | 0 |  |  |  | 0 |  |  |  |
| **Remote guided LSM** | | | | | | | | |
| Overall analysis | 5 | -4.1 (-9.67, 1.35) | 93.73 | 0.00 | 4 | -0.99 (-4.92, 2.91) | 94.97 | NA |
| 12 months and above | 3 | -0.32 (-10.25, 10.03) | 92.32 | NA | 3 | 0.46 (-6.81, 7.76) | 97.88 | NA |
| Asians | 1 | 1.37 (-11.69, 14.3) | NA | NA | 1 | 1.73 (-6.7, 10.45) | NA | NA |
| CaucAsianss | 3 | -2.72 (-7.17, 1.81) | 92.70 | NA | 3 | -2.86 (-6.76, 1.23) | 88.91 | NA |
| **AE + RE** | | | | | | | | |
| Overall analysis | 2 | -8.23 (-16.72, 0.38) | 17.03 | NA | 2 | -11.02 (-16.73, -5.21) | 87.27 | NA |
| 12 months and above | 0 |  |  |  | 0 |  |  |  |
| Asians | 2 | -8.22 (-17.43, 0.88) | 18.99 | NA | 2 | -10.81 (-17.11, -4.58) | 87.97 | NA |
| CaucAsianss | 0 |  |  |  | 0 |  |  |  |
| **LSM + drug** | | | | | | | | |
| Overall analysis | 2 | -11.07 (-18.5, -3.49) | 92.22 | NA | 2 | -10.08 (-14.93, -5.28) | 97.89 | NA |
| 12 months and above | 2 | -11.92 (-22.79, -0.81) | NA | NA | 2 | -10.44 (-16.77, -3.92) | NA | NA |
| Asians | 2 | -11.09 (-19.17, -2.9) | 92.23 | NA | 2 | -9.95 (-15.32, -4.68) | 97.87 | NA |
| CaucAsianss | 0 |  |  |  | 0 |  |  |  |
| **Baduanjin + Acupoint** | | | | | | | | |
| Overall analysis | 1 | -10.48 (-22.33, 1.6) | NA | NA | 1 | 0.02 (-8.28, 8.35) | NA | NA |
| 12 months and above | 0 | -10.55 (-28.24, 6.87) |  |  | 0 | -0.02 (-10.56, 10.67) |  |  |
| Asians | 1 | -10.58 (-23.65, 2.35) | NA | NA | 1 | -0.02 (-8.94, 8.96) | NA | NA |
| CaucAsianss | 0 |  |  |  | 0 |  |  |  |
| **AE + Acupoint** | | | | | | | | |
| Overall analysis | 1 | -1.42 (-13.45, 10.76) | NA | NA | 1 | -0.47 (-8.58, 7.51) | NA | NA |
| 12 months and above | 1 | -1.29 (-18.71, 15.65) | NA | NA | 1 | -0.44 (-11.14, 10.12) | NA | NA |
| Asians | 1 | -1.37 (-14.46, 11.64) | NA | NA | 1 | -0.47 (-9.41, 8.3) | NA | NA |
| CaucAsianss | 0 |  |  |  | 0 |  |  |  |
| **ARB** | | | | | | | | |
| Overall analysis | 6 | -12.5 (-17.59, -7.54) | 99.42 | 0.08 | 6 | -6.42 (-9.69, -3.26) | 94.95 | 0.30 |
| 12 months and above | 5 | -10.13 (-17.65, -2.47) | 99.91 | 0.93 | 5 | -4.45 (-8.95, 0.05) | 99.67 | 0.84 |
| Asians | 3 | -12.86 (-18.45, -7.48) | 99.42 | 0.14 | 3 | -6.56 (-10.24, -2.92) | 94.93 | 0.36 |
| CaucAsianss | 0 |  |  |  | 0 |  |  |  |
| **Beta blocker** | | | | | | | | |
| Overall analysis | 2 | -2.56 (-11.32, 6.31) | 94.71 | NA | 2 | -2.42 (-8.09, 3.44) | 97.36 | NA |
| 12 months and above | 0 |  |  |  | 0 |  |  |  |
| Asians | 0 |  |  |  | 0 |  |  |  |
| CaucAsianss | 2 | -3.01 (-8.66, 3.07) | 93.11 | NA | 2 | -2.89 (-6.86, 1.48) | 96.77 | NA |
| **CCB** | | | | | | | | |
| Overall analysis | 1 | -23.67 (-36.34, -11) |  |  | 1 | -12.44 (-20.81, -4.04) |  |  |
| 12 months and above | 0 |  |  |  | 0 |  |  |  |
| Asians | 0 | -23.63 (-37.4, -10.02) |  |  | 0 | -12.06 (-21.5, -2.95) |  |  |
| CaucAsianss | 0 |  |  |  | 0 |  |  |  |
| **Diuretic** | | | | | | | | |
| Overall analysis | 2 | -7.92 (-14.44, -1.24) | 97.76 | 0.24 | 2 | -3.95 (-8.24, 0.38) | 97.60 | 0.44 |
| 12 months and above | 1 | -7.02 (-18.91, 4.82) | NA | 0.58 | 1 | -3.11 (-10.22, 3.84) | NA | 0.52 |
| Asians | 1 | -9.81 (-18.31, -1.41) | NA | 0.50 | 1 | -5.13 (-10.68, 0.44) | NA | 0.88 |
| CaucAsianss | 1 | -3.4 (-11.12, 4.28) | NA | NA | 1 | -0.59 (-6.01, 4.84) | NA | NA |
| **Statins** | | | | | | | | |
| Overall analysis | 3 | -5.32 (-13.06, 2.41) | 0.00 | NA | 3 | -2.85 (-8.27, 2.53) | 0.00 | NA |
| 12 months and above | 0 | -7.02 (-18.91, 4.82) | NA | 0.58 | 0 | -3.11 (-10.22, 3.84) | NA | 0.52 |
| Asians | 3 | -5.35 (-13.64, 2.97) | 0.00 | NA | 3 | -2.87 (-8.66, 2.93) | 0.00 | NA |
| CaucAsianss | 0 |  |  |  | 0 |  |  |  |
| **Allopurinol** | | | | | | | | |
| Overall analysis | 1 | -5.95 (-19.53, 7.46) | NA | NA | 1 | -5.03 (-13.31, 3.32) | NA | NA |
| 12 months and above | 0 |  |  |  | 0 |  |  |  |
| Asians | 0 |  |  |  | 0 |  |  |  |
| CaucAsianss | 0 |  |  |  | 0 |  |  |  |

CI: confidence interval; RR: risk ratio; SUCRA: surface under the cumulative ranking.

AE, aerobic exercise; HIIT, high-intensity interval training; RE, resistance exercise; DASH, dietary approaches to stop hypertension; TPM, Traditional Persian Medicine; TCD, traditional Chinese drug; EMG, electromyographic; MBSR, mindfulness-based stress reduction; LSM, lifestyle modification; TCM, Traditional Chinese Medicine; ARB, angiotensin II receptor blockers; ACE, angiotensin-converting enzyme inhibitors; CCB, calcium channel blockers.


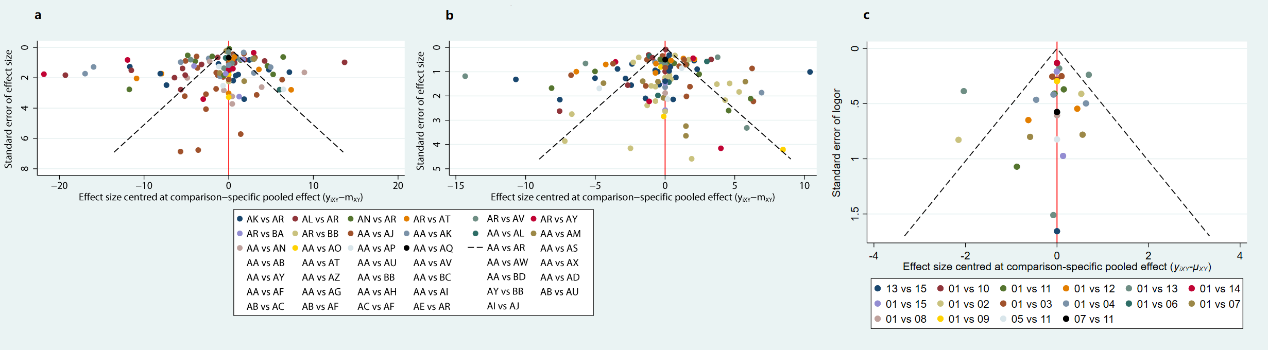


Supplementary Figure 1. Publication bias for (a)SBP, (b)DBP and (c)HT progression rates

AA, control; AB, aerobic exercise; AC, high-intensity interval training; AD, resistance exercise; AE, yoga; AF, dietary approaches to stop hypertension; AG, Traditional Persian Medicine dietary habit; AH, salt restriction; AI, micronutrients; AJ, food extract; AK, medicinal herbs taking ; AL, traditional Chinese drug bubble; AM, electromyographic; AN, acupoint therapy; AO, homeopathy; AP, Lose weight; AQ, mindfulness-based stress reduction; AR, lifestyle modification; AS, Traditional Chinese Medicine constitution intervention; AT, remote guided lifestyle modification; AU, aerobic exercise + resistance exercise; AV, lifestyle modification + drug; AW, baduanjin + acupoint; AX, aerobic exercise + acupoint; AY, angiotensin II receptor blockers; AZ, beta blocker; BA, calcium channel blockers; BB, diuretic; BC, statins; BD, allopurinol.

1, control; 2, dietary approaches to stop hypertension; 3, salt restriction; 4, micronutrients; 5, medicinal herbs taking; 6, traditional Chinese drug bubble; 7, acupoint therapy; 8, homeopathy; 9, Lose weight; 10, mindfulness-based stress reduction; 11, lifestyle modification; 12, remote guided lifestyle modification; 13, aerobic exercise + acupoint; 14, angiotensin II receptor blockers; 15, diuretic.


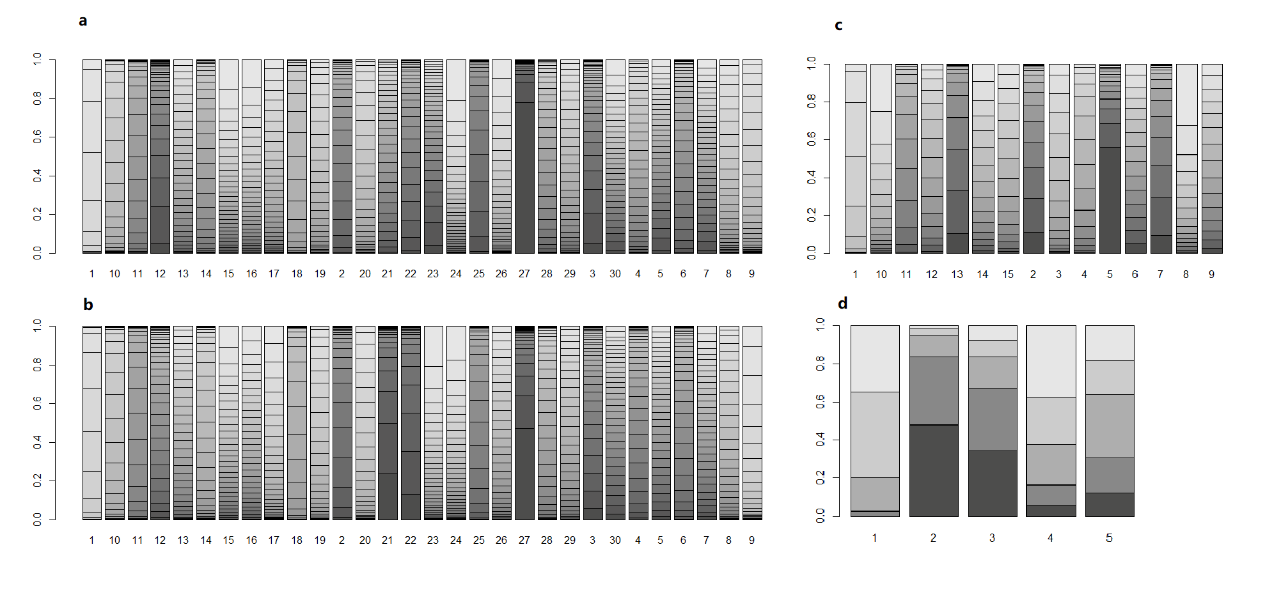


Supplementary Figure 2. SUCRA plots for (a)SBP, (b)DBP, (c)HT progression rates (d)cardiac, renal and mortality outcome incidence


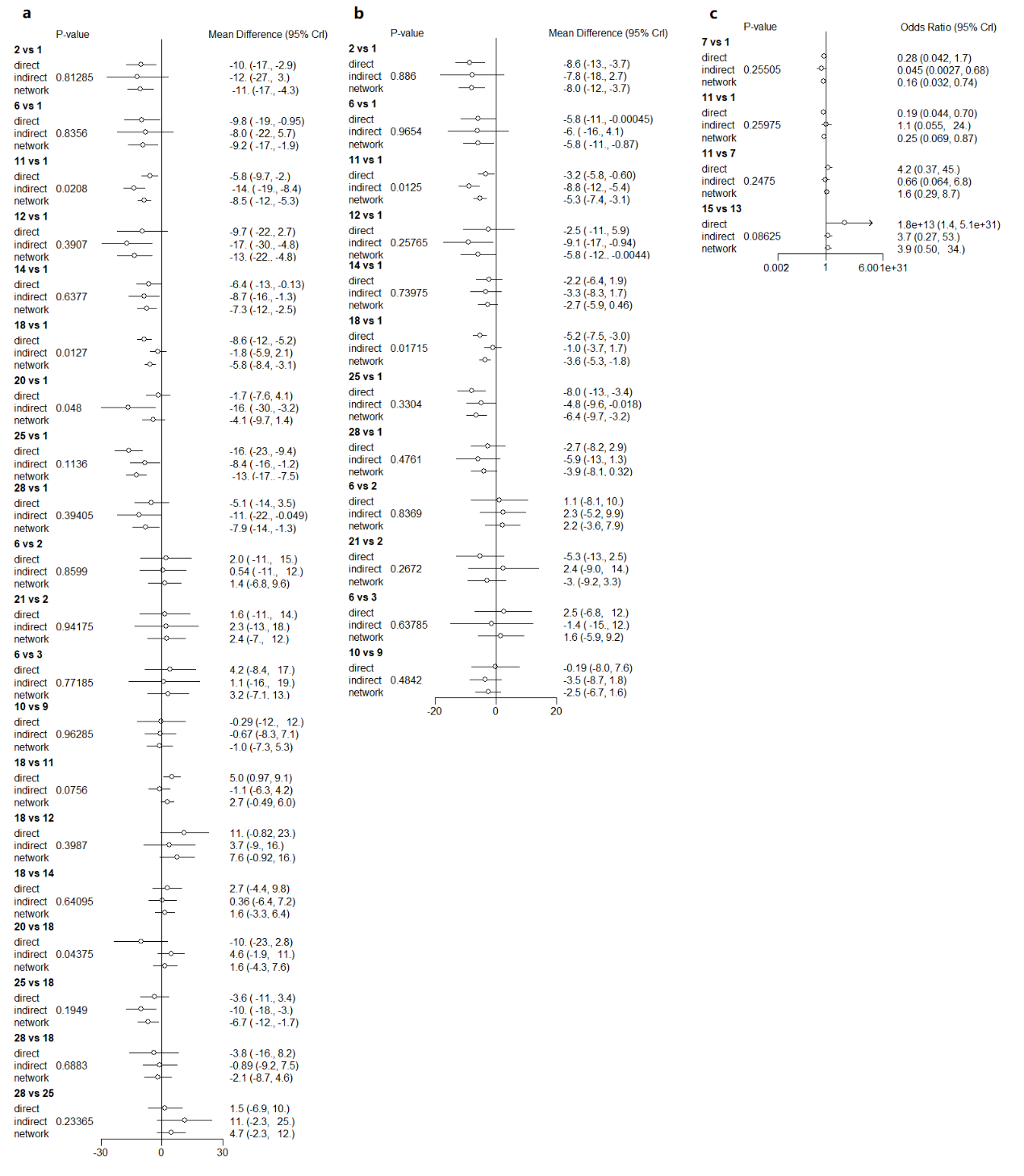


Supplementary Figure 3. Inconsistency plots for (a)SBP, (b)DBP and (c)HT progression rates

(In a, b plots) 1, control; 2, aerobic exercise; 3, high-intensity interval training; 4, resistance exercise; 5, yoga; 6, dietary approaches to stop hypertension; 7, Traditional Persian Medicine dietary habit; 8, salt restriction; 9, micronutrients; 10, food extract; 11, medicinal herbs taking ; 12, traditional Chinese drug bubble; 13, electromyographic; 14, acupoint therapy; 15, homeopathy; 16, Lose weight; 17, mindfulness-based stress reduction; 18, lifestyle modification; 19, Traditional Chinese Medicine constitution intervention; 20, remote guided lifestyle modification; 21, aerobic exercise + resistance exercise; 22, lifestyle modification + drug; 23, baduanjin + acupoint; 24, aerobic exercise + acupoint; 25, angiotensin II receptor blockers; 26, beta blocker; 27, calcium channel blockers; 28, diuretic; 29, statins; 30, allopurinol.

(In c plots) 1, control; 7, acupoint therapy; 11, lifestyle modification; 13, aerobic exercise + acupoint; 15, diuretic.
